# Supplementary material for: Acoustic indices as proxies for bird species richness in an urban green space in Metro Manila
Source: PLoS One. 2023 Jul 28;18(7):e0289001. doi: 10.1371/journal.pone.0289001 (PMC10381043; doi:10.1371/journal.pone.0289001)
Supplement: S1 Fig — (PDF) [file pone.0289001.s001.pdf]

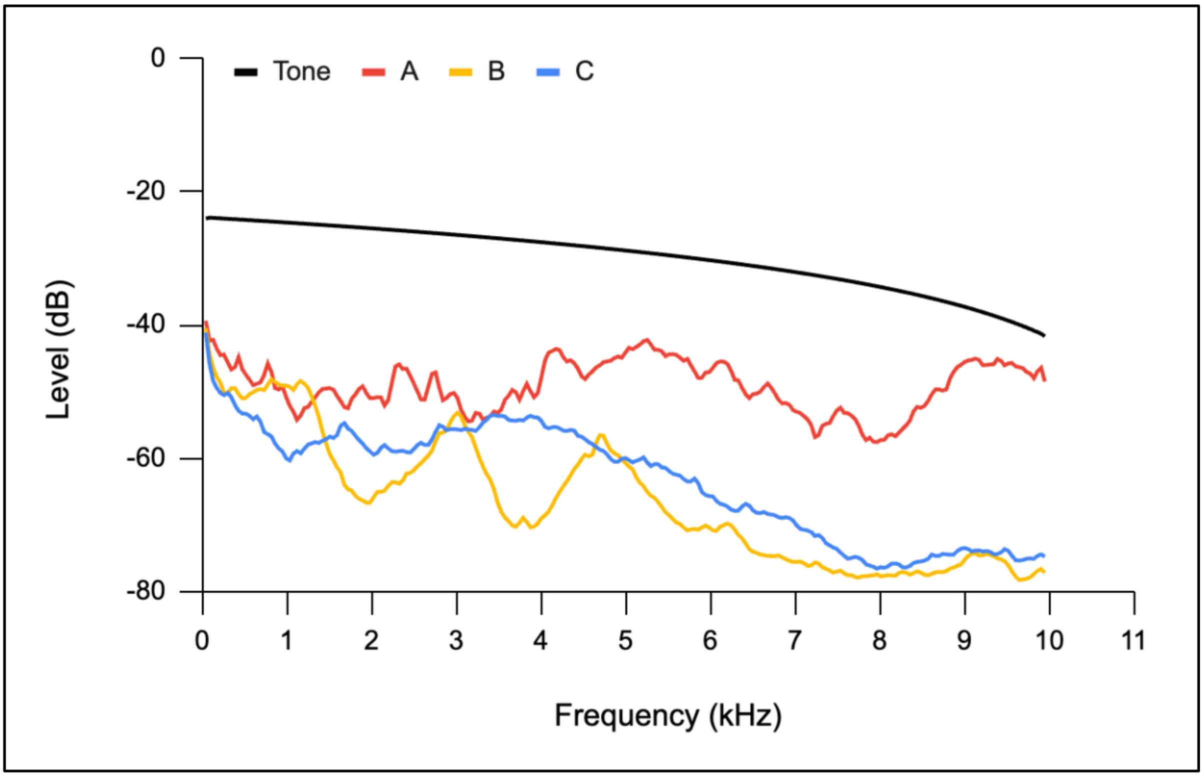

S1 Fig. The frequency response of recorders A, B, and C with respect to the generated tone (black line) in Audacity® [40]. The spectrum was computed using a default 1024-point FFT analysis. The frequency range is 0-10 kHz.
