# Supplementary material for: Acoustic indices as proxies for bird species richness in an urban green space in Metro Manila
Source: PLoS One. 2023 Jul 28;18(7):e0289001. doi: 10.1371/journal.pone.0289001 (PMC10381043; doi:10.1371/journal.pone.0289001)
Supplement: S1 Table — (PDF) [file pone.0289001.s002.pdf]

S1 Table. Summary of parameters used in the calculation of acoustic indices.

Acoustic Index	Parameters	Reference and library [a]
Acoustic Complexity Index (ACI)	min_freq = NA, max_freq = NA, j = 5, fft_w = 512	Pieretti et al. 2011 [1]
Acoustic Diversity Index (ADI)	max_freq = 10000, db_threshold = -50, freq_step = 1000, shannon = TRUE	Villanueva-Rivera et al. 2011 [1]
Acoustic Evenness Index (AEI)	max_freq = 10000, db_threshold = -50, freq_step = 1000	Villanueva-Rivera et al. 2011 [1]
Bioacoustic Index (BI)	min_freq = 2000, max_freq = 8000, fft_w = 512	Boelman et al. 2007 [1]
Acoustic Entropy Index (H)	f = 44100, wl = 512, envt = "hil"	Sueur et al. 2008 [2]
Acoustic Richness Index (AR)	envt = "hil"	Depraetere et al. 2012 [2]

[a] R package used for the index calculation: [1] *soundecology* and [2] *seewave.*
