# Supplementary material for: Acoustic indices as proxies for bird species richness in an urban green space in Metro Manila
Source: PLoS One. 2023 Jul 28;18(7):e0289001. doi: 10.1371/journal.pone.0289001 (PMC10381043; doi:10.1371/journal.pone.0289001)
Supplement: S2 Table — (PDF) [file pone.0289001.s003.pdf]

**S2 Table. The results of GLMM examining how eight acoustic indices reflect bird species richness, season, and time of day, using the dataset of 840 pre-processed 5-min sound samples.**

| | Model | k | $AIC_c$ | $\Delta AIC_c$ | $AIC_cw$ | log(L) |
|---|---|---|---|---|---|---|
| **nACI** | | | | | | |
| 1 | Richness + Season + Hour + Random effect | 6 | -3688.10 | 14.15 | 0.00 | 1850.10 |
| 2 | Richness + Season + Random effect | 5 | -3702.25 | 0.00 | 0.84 | 1856.16 |
| 3 | Richness + Hour + Random effect | 5 | -3698.88 | 3.37 | 0.16 | 1854.48 |
| **ADI** | | | | | | |
| 1 | Richness + Season + Hour + Random effect | 6 | 1160.82 | 0.35 | 0.46 | -574.36 |
| 2 | Richness + Season + Random effect | 5 | 1160.47 | 0.00 | 0.54 | -575.20 |
| 3 | Richness + Hour + Random effect | 5 | 1210.47 | 50.00 | 0.00 | -600.20 |
| *LRT*. 1 *vs* 2: $X^2$=10.763, *d.f.*=1, *p*=0.0010 | | | | | | |
| **1-AEI** | | | | | | |
| 1 | Richness + Season + Hour + Random effect | 6 | -475.08 | 0.00 | 0.87 | 243.59 |
| 2 | Richness + Season + Random effect | 5 | -471.27 | 3.81 | 0.13 | 240.67 |
| 3 | Richness + Hour + Random effect | 5 | -446.98 | 28.10 | 0.00 | 228.53 |
| *LRT*. 1 *vs* 2: $X^2$=5.843, *d.f.*=1, *p* =0.0156 | | | | | | |
| **BI** | | | | | | |
| 1 | Richness + Season + Hour + Random effect | 6 | 4287.74 | 0.00 | 0.57 | -2137.82 |
| 2 | Richness + Season + Random effect | 5 | 4288.33 | 0.59 | 0.43 | -2139.13 |
| 3 | Richness + Hour + Random effect | 5 | 4308.80 | 21.06 | 0.00 | -2149.37 |
| *LRT*. 1 *vs* 2: $X^2$=7.951, *d.f.*=1, *p*=0.0048 | | | | | | |
| **H** | | | | | | |
| 1 | Richness + Season + Hour + Random effect | 6 | -2542.09 | 0.00 | 0.77 | 1277.09 |
| 2 | Richness + Season + Random effect | 5 | -2539.14 | 2.95 | 0.18 | 1274.60 |
| 3 | Richness + Hour + Random effect | 5 | -2536.88 | 5.21 | 0.06 | 1273.47 |
| *LRT*. 1 *vs* 2: $X^2$=4.979, *d.f.*=1, *p*=0.0257 | | | | | | |
| **Ht** | | | | | | |
| 1 | Richness + Season + Hour + Random effect | 6 | -5292.30 | 0.00 | 0.77 | 2652.20 |
| 2 | Richness + Season + Random effect | 5 | -5289.89 | 2.41 | 0.23 | 2649.98 |
| 3 | Richness + Hour + Random effect | 5 | -5280.91 | 11.39 | 0.00 | 2645.49 |
| *LRT*. 1 *vs* 2: $X^2$=4.436, *d.f.*=1, *p* =0.0352 | | | | | | |
| **Hf** | | | | | | |
| 1 | Richness + Season + Hour + Random effect | 6 | -2632.26 | 0.00 | 0.65 | 1322.18 |

| | | | | | | |
|---|---|---|---|---|---|---|
| 2 | Richness + Season + Random effect | 5 | -2629.71 | 2.54 | 0.18 | 1319.89 |
| 3 | Richness + Hour + Random effect | 5 | -2629.46 | 2.79 | 0.16 | 1319.77 |
| *LRT*. 1 *vs* 2: $X^2$=4.574, *d.f.*=1, *p*=0.0325 | | | | | | |
| *LRT*. 1 *vs* 3: $X^2$=4.824, *d.f.* =1, *p*=0.0281 | | | | | | |
| **AR** | | | | | | |
| 1 | Richness + Season + Hour + Random effect | 6 | -59.78 | 0.00 | 1.00 | 35.94 |
| 2 | Richness + Season + Random effect | 5 | -37.18 | 22.60 | 0.00 | 23.63 |
| 3 | Richness + Hour + Random effect | 5 | 21.35 | 81.13 | 0.00 | -5.64 |

We specified the acoustic index as the dependent variable, bird species richness, season, and time of day as fixed effects, and the recording point as the random effect. A common set of three models were fitted: full model (Richness + Season + Hour + Random effect), and fixed effects models (Richness + Season + Random effect; Richness + Hour + Random effect). Normalized Acoustic Complexity index (nACI), Acoustic Diversity index (ADI), Inverse Acoustic Evenness index (1-AEI), Bioacoustic index (BI), Acoustic Entropy index (H), Temporal Entropy Index (Ht), Spectral Entropy Index (Hf), and Acoustic Richness Index (AR). For each model is indicated: k, the number of parameters; $AIC_c$, corrected Akaike Information Criterion; $\Delta AIC_c$, difference in $AIC_c$ values between each model with the lowest $AIC_c$ model; $AIC_cw$, $AIC_c$ weight; and log(L), the log-likelihood. Random effect: (1|Site). LRT, Likelihood-ratio test.
