# Supplementary material for: Acoustic indices as proxies for bird species richness in an urban green space in Metro Manila
Source: PLoS One. 2023 Jul 28;18(7):e0289001. doi: 10.1371/journal.pone.0289001 (PMC10381043; doi:10.1371/journal.pone.0289001)
Supplement: S3 Table — (PDF) [file pone.0289001.s004.pdf]

S3 Table. The results of the acoustic index analysis for 840 raw 5-min sound samples.

WAV	Site	Season	Hour	nACI	ADI	1-AEI	BI	H	Ht	Hf	AR	SH
2021-07-28-12-36-IB100	A	Wet	12	1.07559083	0.38814	0.143843	3.29263888	0.66716821	0.97889301	0.68155376	0.25	1
2021-08-08-14-30-IB100	A	Wet	14	1.04062759	0.047968	0.101831	1.90157493	0.502519	0.94868075	0.52970296	0.109375	1
2021-08-19-18-00-IB100	A	Wet	18	1.006191	2.083415	0.634492	3.30991612	0.74159129	0.99170868	0.74779147	0.30078125	1
2020-09-11-09-54-IB100	A	Wet	09	1.01168017	2.300942	0.967357	4.74863244	0.87663996	0.99134425	0.88429419	0.00286858	0
2020-10-02-10-18-IB100	A	Wet	10	1.0125641	2.29343	0.923184	4.48608279	0.83952714	0.99350019	0.84501961	0.00699292	0
2020-10-04-11-06-IB100	A	Wet	11	1.07001307	1.37266	0.327065	8.68033847	0.5534336	0.99608745	0.55560744	0.89122807	5
2020-10-08-18-00-IB100	A	Wet	18	1.05268483	0.884476	0.198603	4.2779863	0.65760581	0.98644542	0.66664186	0.03567867	0
2020-10-25-06-06-IB100	A	Wet	06	1.20218092	2.289806	0.919309	1.09408915	0.81044176	0.99096941	0.81782723	0.20683287	3
2020-11-01-05-24-IB100	A	Dry	05	1.01564286	0.7046	0.163377	3.98673636	0.69791554	0.98888935	0.70575696	0.00853186	0
2020-11-01-12-12-IB100	A	Dry	12	1.13983514	0.936291	0.271896	1.95848357	0.81064697	0.97216306	0.83385905	0.02880886	2
2020-11-01-16-42-IB100	A	Dry	16	1.07433983	1.054209	0.303133	2.64485279	0.708349	0.96620224	0.73312706	0.03053247	0
2020-11-02-10-18-IB100	A	Dry	10	1.0264045	1.809784	0.466213	1.97701495	0.66704378	0.99129685	0.67290013	0.07074177	5
2020-11-02-11-12-IB100	A	Dry	11	1.02268101	1.914546	0.524646	2.6913031	0.68683036	0.99134418	0.69282735	0.05141274	3
2020-11-05-09-06-IB100	A	Dry	09	1.02220358	1.624741	0.419258	3.72368346	0.63999482	0.99075716	0.64596537	0.49133887	2
2020-11-06-10-48-IB100	A	Dry	10	1.09587544	1.618837	0.412345	1.69220615	0.63032895	0.9911922	0.6359301	0.57772853	1
2020-11-06-12-12-IB100	A	Dry	12	1.02121795	1.447581	0.325586	2.24928898	0.66997364	0.99071401	0.67625332	0.22759003	2
2020-11-06-17-00-IB100	A	Dry	17	1.01603423	0.776582	0.193645	3.88594303	0.6964621	0.9905514	0.70310546	0.24975069	1
2020-11-07-09-30-IB100	A	Dry	09	1.01869872	2.235528	0.801481	2.77324446	0.67470374	0.99160472	0.68041602	0.65152355	1
2020-11-10-12-18-IB100	A	Dry	12	1.01676868	1.207393	0.25836	2.28431105	0.64477042	0.99111991	0.65054734	0.11867036	2
2020-11-14-08-00-IB100	A	Dry	08	1.03353765	1.878224	0.571993	3.86619177	0.65430887	0.9912666	0.66007355	0.51417667	5
2020-11-14-10-00-IB100	A	Dry	10	1.03163498	2.013063	0.61974	1.69908852	0.64743953	0.99129453	0.65312529	0.51732841	3
2020-11-16-08-00-IB100	A	Dry	08	1.02130392	1.855453	0.495067	3.55178114	0.70810006	0.9895839	0.71555333	0.15298246	3
2020-11-16-10-00-IB100	A	Dry	10	1.02328638	1.699674	0.414344	2.82068372	0.66502787	0.99132099	0.67085019	0.17327178	3
2020-11-19-07-00-IB100	A	Dry	07	1.03859458	0.838775	0.193615	4.17245192	0.6760237	0.98499823	0.68631972	0.0128655	4
2020-11-21-11-42-IB100	A	Dry	11	1.01580503	1.317564	0.288618	1.53319561	0.65233074	0.99093327	0.65829937	0.21811019	1

2020-11-21-13-12-IB100	A	Dry	13	1.02212315	0.525025	0.139768	1.88168239	0.60259867	0.9869958	0.61053823	0.14589104	1
2020-11-21-18-36-IB100	A	Dry	18	1.1031774	1.960281	0.564788	9.40587656	0.74889097	0.99066683	0.75594634	0.04678363	0
2020-11-25-15-12-IB100	A	Dry	15	1.02453909	1.609397	0.376946	3.33837437	0.60760292	0.99155802	0.61277596	0.71449677	0
2020-11-25-17-30-IB100	A	Dry	17	1.01700189	2.272408	0.863886	5.27399766	0.75478863	0.99152559	0.76123969	0.2255217	1
2020-11-26-10-48-IB100	A	Dry	10	1.02320399	0.250194	0.117427	2.53700338	0.61820574	0.98758064	0.62598001	0.21540166	2
2020-11-26-16-24-IB100	A	Dry	16	1.01791703	0.324129	0.12215	2.69701847	0.60756733	0.9881116	0.61487723	0.24099723	1
2020-11-27-05-06-IB100	A	Dry	05	1.07671683	2.106503	0.660439	6.4212675	0.74743487	0.98951202	0.75535704	0.03791936	0
2020-11-30-15-24-IB100	A	Dry	15	1.00786862	0.583432	0.167588	1.35402989	0.65340888	0.98874122	0.66084924	0.2128655	1
2020-12-01-08-00-IB100	A	Dry	08	1.02712914	0.861309	0.217618	2.20000533	0.63800902	0.99029993	0.64425836	0.41052632	3
2020-12-02-11-00-IB100	A	Dry	11	1.02610158	1.338249	0.295629	2.29292521	0.60107584	0.99132166	0.60633785	0.5445614	1
2020-12-04-07-48-IB100	A	Dry	07	1.01942588	1.180812	0.256544	4.30690284	0.6260187	0.99144878	0.6314181	0.4806402	5
2020-12-04-13-42-IB100	A	Dry	13	1.00351497	0.195732	0.109959	1.9649742	0.58474003	0.99130553	0.58986862	0.64792859	1
2020-12-07-10-30-IB100	A	Dry	10	1.12155013	1.845539	0.495903	1.86651135	0.684051	0.99123643	0.69009873	0.57982148	0
2020-12-07-12-42-IB100	A	Dry	12	1.04335906	1.811476	0.476324	1.0411925	0.68067274	0.99095076	0.68688856	0.06640813	1
2020-12-09-15-06-IB100	A	Dry	15	1.11717739	1.713782	0.51181	2.46149442	0.79296329	0.98818974	0.80244032	0.25893506	0
2020-12-10-05-42-IB100	A	Dry	05	1.12758207	1.423466	0.335922	2.0146756	0.81961941	0.97941453	0.83684628	0.03854725	2
2020-12-11-08-30-IB100	A	Dry	08	1.00195475	1.413527	0.315676	2.26407102	0.61243378	0.99161773	0.61761075	0.83718067	7
2020-12-18-15-24-IB100	A	Dry	15	1.0191421	0.410118	0.129304	2.4422638	0.65509436	0.9854649	0.66475667	0.06352724	2
2020-12-18-16-54-IB100	A	Dry	16	1.03668912	2.28411	0.901035	2.07947099	0.8193924	0.99094181	0.82688246	0.10649431	1
2020-12-22-07-30-IB100	A	Dry	07	1.04369145	1.63482	0.41505	3.84660107	0.73868507	0.98463874	0.75020923	0.0851462	5
2020-12-25-12-48-IB100	A	Dry	12	1.01879197	0.969009	0.21155	2.21121099	0.71976927	0.98961947	0.72731923	0.04530625	2
2020-12-26-09-42-IB100	A	Dry	09	1.03410712	2.20321	0.756436	2.07207066	0.75503611	0.99121998	0.76172407	0.02708526	3
2020-12-26-18-06-IB100	A	Dry	18	1.05818459	2.272203	0.861449	2.58161689	0.78757524	0.99100672	0.7947224	0.02880886	0
2020-12-28-05-54-IB100	A	Dry	05	1.04305121	1.13549	0.262913	3.84402158	0.72546445	0.98933653	0.7332838	0.04520776	2
2020-12-29-12-00-IB100	A	Dry	12	1.01484392	1.792844	0.458413	2.14901846	0.64691997	0.99127904	0.65261137	0.19769775	2
2020-12-31-16-12-IB100	A	Dry	16	1.023044	0.743241	0.233553	1.23528497	0.70092451	0.96839027	0.72380375	0.01895968	2
2021-01-01-05-12-IB100	A	Dry	05	1.01750972	1.949783	0.564438	6.10695132	0.72876206	0.99133894	0.73512905	0.0141582	1
2021-01-01-08-12-IB100	A	Dry	08	1.01162964	1.161781	0.251224	2.91260408	0.74452947	0.99070971	0.75151123	0.01739612	5

2021-01-05-11-00-IB100	A	Dry	11	1.01555277	1.237772	0.262587	2.11867441	0.64419394	0.99060723	0.65030207	0.12657433	2
2021-01-12-09-12-IB100	A	Dry	09	1.01029981	1.528825	0.429201	4.45448644	0.71668102	0.97379527	0.73596682	0.0102924	4
2021-01-12-17-30-IB100	A	Dry	17	1.01012212	1.963161	0.548855	2.63685182	0.69390271	0.99156916	0.69980264	0.66729455	2
2021-01-14-08-36-IB100	A	Dry	08	1.05464258	1.516084	0.346943	2.94324679	0.71865694	0.98769334	0.72761141	0.23118498	4
2021-01-16-07-54-IB100	A	Dry	07	1.03031348	1.66033	0.407335	2.9562917	0.64027251	0.99145684	0.64578959	0.55953216	4
2021-01-18-14-42-IB100	A	Dry	14	1.02034547	1.212815	0.290399	2.620895	0.67332388	0.98920118	0.68067436	0.28051708	1
2021-01-19-05-42-IB100	A	Dry	05	1.0301437	1.879021	0.535165	7.32865754	0.7485221	0.99117233	0.75518865	0.048261	0
2021-01-27-07-18-IB100	A	Dry	07	1.02286466	1.932141	0.532674	2.55100195	0.78336115	0.99049629	0.79087742	0.11619575	4
2021-01-27-14-54-IB100	A	Dry	14	1.01305722	0.612884	0.168168	1.99240947	0.70153624	0.98865729	0.70958485	0.23955679	1
2021-01-28-06-30-IB100	A	Dry	06	1.01900041	1.739308	0.45152	3.52284083	0.73836882	0.99125097	0.74488585	0.10297322	2
2021-02-04-06-06-IB100	A	Dry	06	1.02086853	1.588741	0.381286	5.68003324	0.70091945	0.98795367	0.70946591	0.04904894	3
2021-02-05-07-00-IB100	A	Dry	07	1.02933336	2.146445	0.691827	3.85076526	0.74475414	0.99114084	0.75141101	0.03564174	6
2021-02-05-08-30-IB100	A	Dry	08	1.11854713	1.112408	0.292743	4.43511117	0.72472156	0.98953713	0.7323844	0.2426839	3
2021-02-05-11-00-IB100	A	Dry	11	1.03567268	0.30273	0.134432	1.76030617	0.59492669	0.98746851	0.60247662	0.24524469	3
2021-02-07-14-00-IB100	A	Dry	14	1.01637179	1.61608	0.40317	1.90763763	0.66186275	0.99133035	0.66765106	0.40316405	2
2021-02-11-08-00-IB100	A	Dry	08	1.07184589	1.966635	0.554759	4.09873597	0.68651399	0.99117759	0.69262461	0.47294552	3
2021-02-12-09-18-IB100	A	Dry	09	1.0213876	0.849863	0.203418	1.95284283	0.61305447	0.9910638	0.61858224	0.08930748	3
2021-02-12-12-30-IB100	A	Dry	12	1.02075506	0.232118	0.119081	2.68803897	0.59000719	0.98640858	0.59813672	0.02427824	7
2021-02-13-09-54-IB100	A	Dry	09	1.13905949	1.973118	0.625019	2.28590103	0.88644269	0.98024792	0.90430458	0.03235457	2
2021-02-15-08-54-IB100	A	Dry	08	1.01589485	1.939952	0.53822	2.10750581	0.63897019	0.99150026	0.64444783	0.45835642	4
2021-02-15-09-00-IB100	A	Dry	09	1.0171767	2.092779	0.645507	2.77759944	0.65041257	0.99150081	0.65598794	0.46330563	4
2021-02-15-14-36-IB100	A	Dry	14	1.00287225	2.300844	0.972693	5.26835463	0.89572837	0.98595112	0.90849166	0.13728532	0
2021-02-16-09-18-IB100	A	Dry	09	1.02097916	0.681339	0.167441	2.38680729	0.70699366	0.98887208	0.71494956	0.06448754	7
2021-02-16-13-24-IB100	A	Dry	13	1.02985305	0.666129	0.170956	5.00897304	0.6898742	0.97743868	0.70579793	0.02677747	3
2021-02-19-12-00-IB100	A	Dry	12	1.0626074	1.204231	0.335139	1.10054383	0.7706842	0.98046653	0.78603825	0.07470606	3
2021-02-20-15-54-IB100	A	Dry	15	1.04624911	0.805092	0.19101	3.65008379	0.72455291	0.98928263	0.73240234	0.0613358	3
2021-02-22-17-12-IB100	A	Dry	17	1.0489714	1.429152	0.325196	2.04024648	0.68294447	0.99042103	0.68954965	0.38675285	2
2021-02-23-10-06-IB100	A	Dry	10	1.0365748	1.7881	0.511463	2.02857553	0.65209694	0.99129057	0.65782623	0.46914127	3

2021-02-26-18-36-IB100	A	Dry	18	1.02634327	0.934611	0.198032	6.87435589	0.74501683	0.98017762	0.7600835	0.02804555	0
2021-03-02-07-54-IB100	A	Dry	07	1.00532015	1.935381	0.534893	1.841231	0.74811522	0.99101754	0.75489605	0.04983687	6
2021-03-03-09-24-IB100	A	Dry	09	1.0297546	2.272362	0.861507	4.12267679	0.76644458	0.99154443	0.77298058	0.67800554	2
2021-03-04-12-00-IB100	A	Dry	12	1.02569801	0.994428	0.222024	2.52240376	0.62378921	0.99116302	0.62935077	0.40603263	3
2021-03-05-18-12-IB100	A	Dry	18	1.02249606	1.123081	0.242494	5.60553833	0.74657474	0.98731826	0.75616422	0.15756233	1
2021-03-06-11-30-IB100	A	Dry	11	1.02493067	1.57307	0.376017	1.71943144	0.63294281	0.99134455	0.63846904	0.44941828	5
2021-03-09-11-06-IB100	A	Dry	11	1.01736768	1.987223	0.559858	2.68802871	0.69647483	0.99143628	0.70249076	0.51245306	2
2021-03-10-06-42-IB100	A	Dry	06	1.01597839	1.911216	0.534492	3.12488044	0.73948955	0.99113467	0.74610401	0.08509695	6
2021-03-10-08-24-IB100	A	Dry	08	1.02611919	2.248944	0.824063	4.98318629	0.75884193	0.99154532	0.7653124	0.5322253	3
2021-03-10-12-30-IB100	A	Dry	12	1.04295344	1.048572	0.242568	4.56496192	0.75723125	0.98532498	0.76850913	0.1235334	0
2021-03-11-14-00-IB100	A	Dry	14	1.03436723	1.775915	0.466651	2.57261875	0.73326157	0.99098301	0.73993355	0.50403201	2
2021-03-12-14-36-IB100	A	Dry	14	1.02519431	0.450122	0.137646	2.61503587	0.70257438	0.98731446	0.71160143	0.19782087	1
2021-03-15-07-24-IB100	A	Dry	07	1.02233953	2.033277	0.59481	6.72150206	0.77650845	0.98861819	0.78544827	0.05489689	4
2021-03-16-15-06-IB100	A	Dry	15	1.04863135	1.316548	0.294218	2.36528304	0.74274091	0.99028631	0.75002643	0.19321637	1
2021-03-16-18-30-IB100	A	Dry	18	1.02220737	0.315024	0.122082	3.94279351	0.67529551	0.98484142	0.68568959	0.04188366	0
2021-03-17-07-54-IB100	A	Dry	07	1.03467657	1.690663	0.431074	2.27725147	0.76653985	0.98886015	0.77517518	0.21392428	4
2021-03-17-16-12-IB100	A	Dry	16	1.0108613	0.017544	0.100572	2.71420704	0.6445243	0.97383646	0.66184039	0.01452755	4
2021-03-17-18-00-IB100	A	Dry	18	1.02983468	1.033002	0.220928	7.55937946	0.73573543	0.9725723	0.75648405	0.02137273	2
2021-03-19-11-24-IB100	A	Dry	11	1.02671695	2.194466	0.742458	3.21683518	0.72070157	0.99156232	0.72683437	0.72701754	4
2021-03-20-16-18-IB100	A	Dry	16	1.02563997	0.676591	0.158598	4.30124048	0.7269101	0.97869378	0.74273498	0.0282056	2
2021-03-21-09-00-IB100	A	Dry	09	1.02568692	1.036471	0.226818	2.82208445	0.69273919	0.98247662	0.70509484	0.00554017	6
2021-03-25-08-06-IB100	A	Dry	08	1.03306794	2.164339	0.713714	2.2883916	0.79912546	0.99078812	0.80655535	0.22001847	5
2021-03-26-05-00-IB100	A	Dry	05	1.0383474	1.959227	0.570717	8.21277701	0.73662669	0.99139438	0.74302084	0.20008618	2
2021-03-27-05-48-IB100	A	Dry	05	1.07277386	2.196623	0.748862	7.06125589	0.76601675	0.99132029	0.77272377	0.12799015	3
2021-03-29-17-36-IB100	A	Dry	17	1.16794667	1.918364	0.543435	2.31637744	0.83660663	0.98152378	0.85235492	0.00837181	2
2021-03-30-05-48-IB100	A	Dry	05	1.06681807	1.771156	0.445473	5.86108674	0.72812709	0.98512501	0.73912151	0.05653432	4
2021-03-30-08-36-IB100	A	Dry	08	1.02254045	2.227886	0.782728	2.734195	0.83486391	0.99099113	0.84245346	0.03526008	3
2021-03-30-12-24-IB100	A	Dry	12	1.03013731	0.303783	0.125139	2.88884991	0.76121913	0.98324573	0.77419012	0.01909511	3

2021-04-01-14-42-IB100	A	Dry	14	1.03322915	0.337686	0.122679	2.37536261	0.74486724	0.9764757	0.76281186	0.00113266	4
2021-04-03-12-42-IB100	A	Dry	12	1.02695817	0.724257	0.17579	3.14218173	0.70044474	0.98562575	0.71065994	0.006008	4
2021-04-03-16-06-IB100	A	Dry	16	1.02646466	0.572631	0.177053	3.26472034	0.76283626	0.98187603	0.77691709	0.00302862	3
2021-04-04-16-00-IB100	A	Dry	16	1.03575412	2.298717	0.95001	2.42869554	0.9157072	0.98919395	0.92571047	0.03072946	2
2021-04-04-18-54-IB100	A	Dry	18	1.0345185	1.223883	0.263364	10.6615446	0.76671586	0.96297755	0.79619287	0.00103416	0
2021-04-07-09-24-IB100	A	Dry	09	1.01465363	1.186542	0.279377	2.10138778	0.6738027	0.99014208	0.68051113	0.20487535	5
2021-04-07-13-18-IB100	A	Dry	13	1.02738369	0.526125	0.140516	2.83839735	0.66365822	0.97702973	0.67926103	0.04520776	4
2021-04-10-10-42-IB100	A	Dry	10	1.01781777	2.202825	0.757218	2.01345894	0.7587605	0.99070482	0.76587948	0.20991074	2
2021-04-11-16-00-IB100	A	Dry	16	1.03288706	0.345093	0.143722	0.92812882	0.69751608	0.95343756	0.73158024	0.00025854	2
2021-04-14-16-48-IB100	A	Dry	16	1.01099743	1.9238	0.531434	2.29737559	0.72716503	0.9912999	0.73354696	0.03826408	3
2021-04-16-05-30-IB100	A	Dry	05	1.0549079	2.093523	0.659214	7.66050638	0.80849858	0.98977684	0.81684936	0.04469067	6
2021-04-16-06-54-IB100	A	Dry	06	1.01951059	1.641761	0.392363	6.92135923	0.790509	0.97328375	0.81220816	0.00819945	4
2021-04-17-07-12-IB100	A	Dry	07	1.0053711	2.057476	0.656237	7.20872971	0.77521646	0.99157415	0.78180382	0.37284087	6
2021-04-18-05-12-IB100	A	Dry	05	1.01568604	2.073659	0.636175	6.72255731	0.79886282	0.99116787	0.80598135	0.00720222	2
2021-04-19-15-48-IB100	A	Dry	15	1.03198568	0.031201	0.1012	1.60067118	0.52771938	0.96413547	0.54734983	0.01891043	2
2021-04-22-10-06-IB100	A	Dry	10	1.01235473	0.992926	0.220573	3.0546848	0.71315186	0.98955063	0.72068254	0.11546938	4
2021-04-22-15-42-IB100	A	Dry	15	1.0207818	0.373028	0.130054	3.24942015	0.58614005	0.97427512	0.60161656	0.06256694	4
2021-04-27-13-48-IB100	A	Dry	13	1.11292826	1.927495	0.5644	2.5079278	0.70759843	0.99120358	0.713878	0.4140474	2
2021-04-29-15-00-IB100	A	Dry	15	1.05662821	1.768568	0.453703	2.16899829	0.7508066	0.97290748	0.77171428	0.04609418	4
2021-05-02-06-06-IB100	A	Wet	06	1.01635927	2.023205	0.589269	2.91185948	0.73768916	0.9921615	0.74351722	0.13146199	5
2021-05-02-08-18-IB100	A	Wet	08	1.02227898	2.016878	0.607878	3.47418879	0.81614565	0.98195211	0.83114609	0.09462604	3
2021-05-04-07-36-IB100	A	Wet	07	1.00729164	1.946254	0.572452	8.80992142	0.80875796	0.99057847	0.81645017	0.47374577	3
2021-05-07-06-12-IB100	A	Wet	06	1.0057909	2.016396	0.626719	8.67671664	0.81433162	0.98113184	0.82999204	0.10179132	5
2021-05-09-13-00-IB100	A	Wet	13	1.01426377	2.119187	0.665401	3.71313478	0.73675304	0.9912697	0.74324176	0.10489381	2
2021-05-13-11-30-IB100	A	Wet	11	1.01923386	1.618304	0.45074	7.27587412	0.78128082	0.97830572	0.79860601	0.06282549	2
2021-05-13-13-36-IB100	A	Wet	13	1.0089031	1.415562	0.33024	5.52469735	0.75479673	0.98457579	0.76662126	0.10485688	4
2021-05-13-15-06-IB100	A	Wet	15	1.01555533	1.835211	0.523228	7.67196083	0.7990416	0.97617485	0.81854352	0.06040012	2
2021-05-15-12-18-IB100	A	Wet	12	1.00098873	2.103049	0.654317	4.62812783	0.76291515	0.99142404	0.76951447	0.3309326	3

2021-05-16-11-54-IB100	A	Wet	11	1.01243368	1.772058	0.497688	7.18245535	0.81865962	0.9801965	0.83519949	0.08574946	0
2021-05-17-13-00-IB100	A	Wet	13	1.01477954	1.616666	0.443607	5.90333579	0.79548784	0.98299684	0.80924761	0.07871961	0
2021-05-18-07-54-IB100	A	Wet	07	1.02957192	2.29821	0.949475	6.08181342	0.83690929	0.98716097	0.84779414	0.25263158	0
2021-05-18-08-00-IB100	A	Wet	08	1.01177519	2.228458	0.795783	8.66373709	0.79601724	0.98101067	0.81142567	0.12304094	0
2021-05-18-14-36-IB100	A	Wet	14	1.00277081	2.161596	0.716939	7.34513046	0.80614372	0.97827632	0.82404501	0.0646599	4
2021-05-21-07-54-IB100	A	Wet	07	1.01718517	2.070271	0.6414	8.13103262	0.81308374	0.99015954	0.82116438	0.44674669	0
2021-05-21-17-30-IB100	A	Wet	17	1.01468579	0.049302	0.10313	4.02156343	0.60507354	0.96019621	0.63015615	0.00669745	3
2021-05-23-15-48-IB100	A	Wet	15	1.0267512	2.134847	0.70136	5.16669577	0.77493501	0.99150024	0.78157824	0.37838104	2
2021-05-23-18-30-IB100	A	Wet	18	1.02412017	1.011211	0.221759	4.15580406	0.71876051	0.98575943	0.72914393	0.06106494	2
2021-05-25-18-54-IB100	A	Wet	18	1.06376653	2.23278	0.795194	6.60530297	0.73004485	0.99167353	0.73617459	0.32840874	0
2021-05-27-06-42-IB100	A	Wet	06	1.01428147	2.219648	0.827416	14.971085	0.78337144	0.99138665	0.79017752	0.82043706	1
2021-05-27-16-12-IB100	A	Wet	16	1.02580755	1.412524	0.323246	4.77595438	0.6609256	0.98964185	0.66784322	0.30419206	3
2021-05-28-10-54-IB100	A	Wet	10	1.00686038	2.116599	0.678376	6.55327725	0.77211172	0.99082823	0.7792589	0.48659895	4
2021-05-30-14-00-IB100	A	Wet	14	1.00763349	1.633871	0.444038	7.63222053	0.80002902	0.98002448	0.81633574	0.10522622	2
2021-06-01-10-24-IB100	A	Wet	10	1.01476858	1.901448	0.513657	3.98677161	0.81100708	0.97195169	0.8344109	0.0344229	2
2021-06-03-10-54-IB100	A	Wet	10	1.01418842	1.776133	0.50955	9.27730348	0.80657151	0.98801148	0.81635844	0.28336103	2
2021-06-06-06-48-IB100	A	Wet	06	1.01355171	2.236816	0.842049	14.5777638	0.7935109	0.99127903	0.80049197	0.74725762	2
2021-06-08-07-24-IB100	A	Wet	07	1.02516422	2.296795	0.946142	6.66539756	0.84433114	0.99015169	0.85272908	0.4449369	1
2021-06-08-14-18-IB100	A	Wet	14	1.03805219	2.302261	0.985537	1.67595512	0.92351884	0.99008499	0.93276723	0.4443952	0
2021-06-09-14-42-IB100	A	Wet	14	1.00519701	1.44254	0.354122	2.2699469	0.68804225	0.98980982	0.69512571	0.32743613	2
2021-06-09-17-18-IB100	A	Wet	17	1.01733237	2.253345	0.826599	2.11917271	0.69488993	0.99160964	0.70076964	0.47377039	3
2021-06-11-14-36-IB100	A	Wet	14	0.99556224	0.630891	0.161027	3.39293779	0.61981012	0.99086877	0.6255219	0.49048938	4
2021-06-11-16-54-IB100	A	Wet	16	1.12114945	2.2971	0.945644	1.86901211	0.82502575	0.99135626	0.83221923	0.22372422	3
2021-06-12-09-12-IB100	A	Wet	09	1.01003409	2.012435	0.623769	8.3648717	0.82807742	0.99069671	0.83585361	0.35075408	4
2021-06-13-06-42-IB100	A	Wet	06	1.00765564	2.226555	0.819078	12.5976618	0.80609322	0.99127169	0.813191	0.6955494	4
2021-06-15-16-18-IB100	A	Wet	16	1.01781282	1.332077	0.290442	4.90822587	0.73127999	0.98452109	0.74277738	0.0947984	2
2021-06-17-17-18-IB100	A	Wet	17	1.02492824	0.64429	0.188851	3.23401539	0.72614592	0.98531046	0.73697169	0.09894737	3
2021-06-18-14-48-IB100	A	Wet	14	1.003008	1.70561	0.443088	7.26321478	0.81393164	0.98335648	0.82770761	0.1417667	5

2021-06-19-16-42-IB100	A	Wet	16	1.00869175	0.118205	0.108376	3.33647215	0.67869258	0.98230184	0.69092061	0.08017236	2
2021-06-20-05-30-IB100	A	Wet	05	1.01207052	2.236747	0.830047	13.6704078	0.78900926	0.98986646	0.79708657	0.41524161	3
2021-06-20-10-24-IB100	A	Wet	10	1.0048853	1.939494	0.571679	7.01624042	0.81465541	0.99061881	0.82237023	0.25300092	4
2021-06-22-08-54-IB100	A	Wet	08	1.00264226	2.164151	0.708955	5.25307341	0.82220067	0.99097239	0.82969079	0.169012	4
2021-06-22-15-48-IB100	A	Wet	15	0.99718115	0.761343	0.179364	4.52833328	0.71525332	0.97313262	0.73500087	0.04269621	4
2021-06-22-16-54-IB100	A	Wet	16	1.00889753	1.682845	0.448887	2.76211377	0.72316815	0.99106771	0.72968592	0.30389658	5
2021-06-23-07-00-IB100	A	Wet	07	1.01709499	2.228366	0.825152	13.319106	0.79300073	0.99123286	0.80001457	0.68390274	4
2021-06-25-09-00-IB100	A	Wet	09	1.01226921	2.055309	0.645515	12.1082329	0.79975393	0.98985612	0.80794967	0.41037858	4
2021-06-26-07-42-IB100	A	Wet	07	1.01529023	2.300074	0.961322	7.60805965	0.82693144	0.99030839	0.83502417	0.45710065	2
2021-06-28-06-18-IB100	A	Wet	06	1.00993027	2.268776	0.864768	7.38388768	0.81078667	0.98933467	0.8195272	0.11065559	4
2021-06-29-11-42-IB100	A	Wet	11	0.99722641	0.401239	0.129215	2.21902308	0.55110772	0.98952076	0.55694407	0.35804248	3
2021-07-03-14-24-IB100	A	Wet	14	1.17615182	2.302432	0.990259	2.29005706	0.86971005	0.99041513	0.87812678	0.3567867	0
2021-07-04-16-48-IB100	A	Wet	16	1.01660613	0.378479	0.144401	1.83660917	0.72298641	0.98195687	0.73627104	0.02276393	0
2021-07-08-09-00-IB100	A	Wet	09	1.00390969	2.143457	0.709312	5.45118576	0.82858157	0.99100933	0.83609866	0.59720529	1
2021-07-08-15-36-IB100	A	Wet	15	1.02510017	1.701687	0.438186	2.91475153	0.72576643	0.99091464	0.73242074	0.27578947	1
2021-07-10-06-18-IB100	A	Wet	06	1.00266892	1.736748	0.492122	8.70569072	0.79395248	0.98852005	0.80317286	0.18736842	4
2021-07-13-06-06-IB100	A	Wet	06	0.99617322	1.767209	0.462235	5.86365363	0.73149667	0.98725114	0.74094285	0.1365097	4
2021-07-13-13-54-IB100	A	Wet	13	1.07821207	1.701453	0.426523	3.2484182	0.68510748	0.98995181	0.69206144	0.37850416	4
2021-07-13-17-00-IB100	A	Wet	17	1.01550119	2.280522	0.892307	14.0526471	0.83404651	0.97864393	0.85224716	0.08480148	5
2021-07-15-17-18-IB100	A	Wet	17	1.01270398	2.116989	0.703784	12.1216719	0.82929647	0.98684261	0.84035332	0.17108033	2
2021-07-15-18-54-IB100	A	Wet	18	1.01738337	1.596297	0.375677	6.7331006	0.75309928	0.99097616	0.759957	0.05012004	0
2021-07-16-15-30-IB100	A	Wet	15	1.0261898	2.032823	0.667741	14.6603056	0.82988989	0.98685	0.84094836	0.24176054	1
2021-07-17-06-48-IB100	A	Wet	06	1.01303762	2.244831	0.820531	7.28237056	0.80962038	0.98992133	0.81786336	0.19698369	3
2021-07-18-13-06-IB100	A	Wet	13	1.02088097	1.759558	0.449818	7.50083807	0.78597392	0.98810514	0.79543552	0.18313327	6
2021-07-19-06-00-IB100	A	Wet	06	1.12201724	2.280965	0.892307	2.14168669	0.79311984	0.99128507	0.80009259	0.44552786	3
2021-07-19-16-18-IB100	A	Wet	16	1.00819697	1.735597	0.434204	3.57634653	0.69733884	0.9913103	0.70345163	0.51523546	5
2021-07-21-12-12-IB100	A	Wet	12	1.1021577	2.302309	0.987492	2.28183611	0.86487311	0.99113281	0.87261071	0.57141274	1
2021-07-23-18-12-IB100	A	Wet	18	1.0598406	2.293179	0.92405	4.07651969	0.88504233	0.99051613	0.89351632	0.48930748	0

2021-07-24-05-00-IB100	A	Wet	05	1.11327545	2.302488	0.992281	2.25801238	0.88303989	0.99081247	0.89122808	0.45553709	0
2021-07-25-06-48-IB100	A	Wet	06	1.11382187	1.875598	0.563325	1.297954	0.8283127	0.96067853	0.86221631	0.01569714	1
2021-07-25-08-12-IB100	A	Wet	08	1.10237297	2.302572	0.997265	1.73618972	0.89695853	0.99049606	0.90556496	0.36363189	2
2021-07-25-09-00-IB100	A	Wet	09	1.12853845	2.302482	0.992454	1.87512637	0.87532754	0.99066699	0.88357394	0.43512465	1
2021-07-25-17-06-IB100	A	Wet	17	1.01763992	2.281489	0.917295	12.256407	0.84413408	0.99108137	0.85173035	0.61335796	1
2021-07-27-10-30-IB100	A	Wet	10	1.07856242	1.16611	0.278344	2.4013194	0.77771952	0.97953395	0.79396893	0.09967375	2
2021-07-27-11-36-IB100	A	Wet	11	1.05858949	0.866023	0.198661	2.96040447	0.66732165	0.97272113	0.68603593	0.04819945	2
2021-08-01-09-30-IB100	A	Wet	09	1.02372618	0.124389	0.113458	2.17199952	0.58742138	0.96429777	0.60917012	0.02437673	5
2021-08-01-16-48-IB100	A	Wet	16	1.02334008	0.031721	0.101134	2.10097487	0.5991959	0.95563814	0.62701129	0.00526931	2
2021-08-01-17-54-IB100	A	Wet	17	1.00905135	0.09242	0.104481	2.52081412	0.64420972	0.9854159	0.65374399	0.16270853	0
2021-08-02-11-18-IB100	A	Wet	11	1.06077977	0.187335	0.110406	2.1830513	0.54740606	0.96192254	0.56907499	0.01964912	2
2021-08-03-05-00-IB100	A	Wet	05	1.1758303	2.300736	0.966312	1.7377823	0.83887284	0.99101335	0.84647986	0.44609418	0
2021-08-04-07-12-IB100	A	Wet	07	1.01632018	1.802045	0.496943	3.71893651	0.79368374	0.97915308	0.81058188	0.09492151	2
2021-08-05-05-00-IB100	A	Wet	05	1.00907102	1.818211	0.47308	5.57121312	0.67513167	0.99149281	0.68092442	0.49950139	2
2021-08-05-13-48-IB100	A	Wet	13	1.02033196	1.714484	0.456568	4.95638867	0.86834255	0.9833367	0.8830572	0.15778393	0
2021-08-06-05-00-IB100	A	Wet	05	1.06165156	2.086367	0.645495	7.77658767	0.73074448	0.99137473	0.73710219	0.43475531	0
2021-08-12-08-18-IB100	A	Wet	08	1.01147355	2.200952	0.757853	4.98061614	0.80084866	0.99053472	0.80850135	0.40982456	4
2021-08-13-07-06-IB100	A	Wet	07	0.99505357	2.134194	0.679259	3.96951673	0.75177537	0.9915501	0.75818193	0.1845614	5
2021-08-13-12-06-IB100	A	Wet	12	1.02657549	1.727048	0.431276	2.85338033	0.66810681	0.99148374	0.67384546	0.27502616	3
2021-08-16-11-54-IB100	A	Wet	11	1.03479867	0.618296	0.183168	4.75905447	0.68749582	0.98478365	0.69811864	0.09461373	6
2021-08-18-06-48-IB100	A	Wet	06	0.99815753	2.08636	0.639306	3.98504397	0.75909471	0.99168153	0.76546219	0.19028624	7
2021-08-21-17-06-IB100	A	Wet	17	1.06067138	2.282648	0.890962	2.25485761	0.87506891	0.98639204	0.88714109	0.21281625	0
2021-08-24-16-12-IB100	A	Wet	16	1.053285	2.302508	0.993067	2.31737393	0.88170574	0.99126002	0.88947977	0.40784241	1
2021-08-24-18-42-IB100	A	Wet	18	1.05223561	2.052564	0.623121	2.83491021	0.76733698	0.99130632	0.77406647	0.2426839	0
2021-08-25-16-12-IB100	A	Wet	16	1.08903159	2.174409	0.730456	2.31377333	0.73015897	0.99143088	0.73646987	0.48363189	2
2021-08-27-12-18-IB100	A	Wet	12	1.01115352	0.573381	0.15432	3.1157641	0.63604029	0.98672916	0.6445946	0.09440443	3
2021-08-28-05-24-IB100	A	Wet	05	1.00472359	2.103824	0.666474	8.04021056	0.7654811	0.99147786	0.77206071	0.36934441	1
2021-08-28-07-18-IB100	A	Wet	07	1.02047724	2.046476	0.607462	7.41667682	0.70860199	0.99112525	0.71494696	0.47017544	4

2021-08-28-11-30-IB100	A	Wet	11	1.0195365	2.054996	0.631179	8.23346369	0.81333049	0.98907267	0.82231621	0.22352724	1
2021-08-28-12-30-IB100	A	Wet	12	1.03080974	2.294258	0.928641	2.2951354	0.88062145	0.98896426	0.8904482	0.3133518	1
2021-08-29-18-30-IB100	A	Wet	18	1.02409119	2.15296	0.72578	4.82372239	0.75424081	0.99175027	0.76051485	0.20802709	1
2021-09-03-13-30-IB100	A	Wet	13	1.03806618	2.078516	0.65533	2.03776065	0.76383451	0.99052657	0.77113985	0.20529394	0
2021-09-06-14-42-IB100	A	Wet	14	1.07091766	2.268453	0.856774	2.26142395	0.7462553	0.99147305	0.7526733	0.39852262	3
2021-09-08-17-42-IB100	A	Wet	17	1.04026796	2.244717	0.816793	2.66046772	0.76305398	0.991555	0.76955285	0.38677747	1
2021-09-13-05-30-IB100	A	Wet	05	1.01513385	2.178608	0.74326	7.69999833	0.76945646	0.99152675	0.77603198	0.33799938	4
2021-09-13-06-54-IB100	A	Wet	06	1.00156186	1.77996	0.461838	3.96188081	0.71086394	0.99127472	0.71712102	0.35469375	5
2021-09-13-13-18-IB100	A	Wet	13	1.01858487	1.216612	0.285668	4.53439159	0.71136159	0.98893492	0.71932093	0.14035088	3
2021-09-16-07-48-IB100	A	Wet	07	1.00146859	0.994727	0.219563	2.22902974	0.71728223	0.98697255	0.72674992	0.09292705	4
2021-09-19-10-42-IB100	A	Wet	10	1.01079799	0.433838	0.142937	2.99274501	0.76267297	0.99075832	0.7697871	0.11819021	4
2021-09-23-13-06-IB100	A	Wet	13	1.02783794	2.292368	0.938567	2.02918208	0.83343951	0.99027456	0.84162468	0.39815328	1
2021-09-25-08-06-IB100	A	Wet	08	0.99845197	2.026577	0.601438	5.71949536	0.77454565	0.99046411	0.78200274	0.13961219	5
2021-09-25-15-24-IB100	A	Wet	15	1.01116121	1.026182	0.220172	3.28398329	0.53607956	0.99259352	0.54007965	0.97207756	0
2021-09-26-13-42-IB100	A	Wet	13	1.01157618	2.249388	0.82458	2.53233856	0.75721765	0.99134574	0.76382802	0.20252385	2
2021-09-27-08-42-IB100	A	Wet	08	1.06889246	1.72783	0.5007	1.97851307	0.80422204	0.98653318	0.8152002	0.12927055	5
2021-09-27-14-12-IB100	A	Wet	14	0.99956476	1.727525	0.441415	3.276997	0.72002287	0.99079076	0.72671537	0.2433241	5
2021-09-27-17-12-IB100	A	Wet	17	0.99956313	1.390571	0.338586	3.38888198	0.76413639	0.9908489	0.77119366	0.27423823	2
2021-09-30-05-18-IB100	A	Wet	05	1.0549834	2.302387	0.988983	2.38635914	0.88049797	0.99087956	0.88860242	0.31231764	0
2021-10-01-13-06-IB100	A	Wet	13	1.01392675	0.675838	0.184298	4.25230221	0.70160531	0.9886251	0.70967783	0.20274546	0
2021-10-01-17-00-IB100	A	Wet	17	1.01747548	2.041219	0.648813	12.2653785	0.82675201	0.98810951	0.83670079	0.27104955	1
2021-10-05-09-54-IB100	A	Wet	09	1.05518585	2.272818	0.869159	2.52461029	0.87518135	0.98887473	0.88502752	0.22682672	0
2021-10-05-10-48-IB100	A	Wet	10	1.06436197	2.231693	0.790912	2.94822595	0.81208449	0.99122516	0.81927348	0.38356417	3
2021-10-12-15-06-IB100	A	Wet	15	1.07448868	0.340834	0.135395	1.48545518	0.71099854	0.98911445	0.71882333	0.28024623	4
2021-10-15-12-00-IB100	A	Wet	12	1.01555072	1.194602	0.253322	2.48842971	0.57964455	0.99145449	0.58464061	0.56405048	2
2021-10-15-18-12-IB100	A	Wet	18	1.09707046	2.230607	0.797012	6.38568848	0.78411142	0.99154139	0.79080049	0.53776547	0
2021-10-18-12-48-IB100	A	Wet	12	1.01610228	0.407204	0.132271	3.78548062	0.61524621	0.98631927	0.62377998	0.16982456	1
2021-10-18-14-06-IB100	A	Wet	14	1.01665249	1.00827	0.21106	3.21168565	0.62697601	0.99133973	0.63245322	0.61144968	2

2021-10-19-18-06-IB100	A	Wet	18	1.0140523	0.914698	0.197346	6.99272066	0.68102249	0.99048388	0.68756544	0.32982456	1
2021-10-22-06-54-IB100	A	Wet	06	1.01593228	1.488616	0.343275	3.28990629	0.69841689	0.99225266	0.70387001	0.43397969	3
2021-10-24-17-42-IB100	A	Wet	17	1.01506343	2.204916	0.759105	7.34738072	0.74906789	0.99176769	0.75528563	0.43124654	1
2021-10-25-10-48-IB100	A	Wet	10	1.03437069	1.577993	0.368594	2.3328021	0.6777673	0.99164835	0.68347545	0.67220683	3
2021-10-29-13-18-IB100	A	Wet	13	1.0194639	1.84838	0.489771	3.18091477	0.63179366	0.99149968	0.63721015	0.74132348	0
2021-10-30-15-48-IB100	A	Wet	15	1.01920999	1.564293	0.371094	3.91618018	0.68764543	0.99063084	0.69414902	0.14128655	1
2021-11-01-14-30-IB100	A	Dry	14	1.02225709	0.970779	0.20574	2.71003949	0.71133974	0.9885355	0.71958947	0.06426593	0
2021-11-03-15-54-IB100	A	Dry	15	1.01049599	0.491852	0.135958	3.22823637	0.68316236	0.99070546	0.68957161	0.39372115	0
2021-11-08-17-48-IB100	A	Dry	17	1.03622448	1.176101	0.2492	3.86904685	0.72370574	0.98976367	0.73119044	0.2245614	0
2021-12-26-10-18-IB100	A	Dry	10	1.01810615	1.87909	0.498895	3.87174765	0.69012126	0.99206953	0.695638	0.09307479	4
2022-01-01-05-24-IB100	A	Dry	05	1.01044089	1.521769	0.355707	6.02649337	0.69754129	0.98605148	0.70740859	0.06697445	0
2022-01-02-17-54-IB100	A	Dry	17	1.00228895	2.138873	0.685289	7.26896538	0.78228475	0.99165917	0.78886454	0.21589412	1
2022-01-04-14-30-IB100	A	Dry	14	1.03409555	1.034775	0.23895	2.27774152	0.75579065	0.99087516	0.76275062	0.21999384	3
2022-01-04-15-00-IB100	A	Dry	15	1.02160461	2.069111	0.640634	2.22429983	0.72150036	0.99147179	0.7277064	0.29921822	1
2022-01-05-18-54-IB100	A	Dry	18	1.03165541	1.947202	0.57284	9.30642754	0.78228161	0.99151109	0.78897918	0.23730379	0
2022-01-06-13-06-IB100	A	Dry	13	1.01407999	0.993605	0.212343	3.44080436	0.63190205	0.99106302	0.63760028	0.11843644	3
2022-01-08-11-18-IB100	A	Dry	11	1.01762502	0.712295	0.172084	4.38320361	0.64029092	0.9897369	0.64693043	0.05127732	7
2022-01-09-17-54-IB100	A	Dry	17	1.05247355	2.284752	0.896704	10.1679709	0.83593709	0.99088502	0.84362674	0.13604186	0
2022-01-10-11-06-IB100	A	Dry	11	1.02014153	0.565052	0.149568	2.99178396	0.61368585	0.98949469	0.62020126	0.13956294	4
2022-01-11-18-30-IB100	A	Dry	18	1.03535816	1.881183	0.518843	4.63242976	0.73029814	0.9910099	0.73692315	0.22182826	0
2022-01-15-08-18-IB100	A	Dry	08	1.03072504	1.567501	0.394903	3.17529662	0.78039598	0.98616301	0.79134583	0.09843029	3
2022-01-15-11-54-IB100	A	Dry	11	1.01327609	1.55389	0.35929	3.03860458	0.64182	0.99159028	0.6472633	0.34773777	3
2022-01-15-18-24-IB100	A	Dry	18	1.04495882	1.806169	0.47889	5.61268676	0.73661724	0.98968318	0.74429601	0.18449985	0
2022-01-16-06-42-IB100	A	Dry	06	1.00584582	2.019171	0.590767	4.91517026	0.69570181	0.99182032	0.70143936	0.39844875	3
2022-01-17-13-24-IB100	A	Dry	13	1.02819751	0.551068	0.150671	1.87346071	0.67392375	0.98863539	0.68167068	0.18204986	4
2022-01-24-10-00-IB100	A	Dry	10	1.00345335	2.082524	0.636497	2.78519833	0.71193073	0.99145557	0.7180662	0.21806094	4
2022-01-24-15-42-IB100	A	Dry	15	1.01442637	1.412638	0.314804	2.6667254	0.67373132	0.99144748	0.67954312	0.16454294	5
2022-01-29-09-12-IB100	A	Dry	09	1.0082733	1.545	0.355174	3.35611134	0.72481612	0.99088205	0.73148576	0.11027393	4

2022-01-29-13-54-IB100	A	Dry	13	1.02027118	1.14629	0.248593	3.09497624	0.66417195	0.99040646	0.67060544	0.07590028	5
2022-01-30-09-48-IB100	A	Dry	09	1.01704217	0.259264	0.123391	4.10540102	0.60482286	0.95599432	0.63266365	0.00369344	5
2022-01-31-11-00-IB100	A	Dry	11	1.01563934	1.763089	0.451149	2.22858086	0.66271405	0.9912512	0.66856317	0.23630348	6
2020-07-17-08-36-ADMIN	B	Wet	08	1.01718564	1.218374	0.2654	4.79352504	0.70753163	0.97523941	0.72549532	0.0349646	6
2020-07-19-07-42-ADMIN	B	Wet	07	1.06376967	1.961051	0.567961	5.12449213	0.75732245	0.98737426	0.76700647	0.39022469	5
2020-08-16-09-42-ADMIN	B	Wet	09	1.02758446	0.763497	0.178345	2.27522571	0.72575137	0.98920561	0.7336709	0.14164358	4
2020-08-25-09-12-ADMIN	B	Wet	09	1.03981403	0.683992	0.171666	3.75435517	0.64679043	0.98665684	0.65553737	0.29577101	1
2020-08-27-17-30-ADMIN	B	Wet	17	1.02800629	0.616088	0.156114	2.66069751	0.6982281	0.98758845	0.7070031	0.22714681	0
2020-08-30-14-12-ADMIN	B	Wet	14	1.02579704	0.633108	0.16006	2.74696612	0.69022581	0.98619851	0.69988527	0.15546938	3
2020-09-01-17-18-ADMIN	B	Wet	17	1.03280994	0.760001	0.174826	3.04438427	0.67796847	0.98689092	0.68697407	0.21037858	2
2020-09-07-07-18-ADMIN	B	Wet	07	1.03192509	0.541199	0.143478	3.22989418	0.7045391	0.98447548	0.71564921	0.07290859	3
2020-09-08-12-30-ADMIN	B	Wet	12	1.03653403	0.597745	0.155787	2.06404866	0.64826675	0.98720341	0.65666989	0.32233918	2
2020-09-12-17-42-ADMIN	B	Wet	17	1.04335814	0.500514	0.137339	3.46706992	0.69279035	0.98153374	0.70582428	0.04155125	0
2020-09-16-16-18-ADMIN	B	Wet	16	1.03004611	1.003811	0.218399	5.35092965	0.65585826	0.98640028	0.66490073	0.28100954	3
2020-09-19-09-30-ADMIN	B	Wet	09	1.02067901	1.254789	0.279029	5.57042502	0.69585879	0.98578512	0.70589297	0.20176054	2
2020-09-20-12-54-ADMIN	B	Wet	12	1.03503832	1.688613	0.410068	5.54045699	0.77853014	0.98890774	0.78726267	0.10518929	1
2020-10-08-07-18-ADMIN	B	Wet	07	1.03542503	0.323276	0.1192	2.58025357	0.68069444	0.98469692	0.69127304	0.08386581	4
2020-10-09-05-12-ADMIN	B	Wet	05	1.04989196	0.792159	0.176315	4.36528375	0.70741481	0.98456626	0.71850401	0.04924592	0
2020-10-11-05-42-ADMIN	B	Wet	05	1.04436051	1.372226	0.301157	4.59133927	0.74493777	0.98775983	0.75416893	0.08322561	1
2020-10-14-17-00-ADMIN	B	Wet	17	1.0316434	1.036056	0.219701	5.93352606	0.71959558	0.98956841	0.72718124	0.50607572	2
2020-10-18-06-54-ADMIN	B	Wet	06	1.02912692	1.072627	0.225893	4.8054096	0.73290462	0.98676802	0.74273245	0.12345953	3
2020-10-20-18-42-ADMIN	B	Wet	18	1.01965697	1.597204	0.38821	6.88239371	0.6915842	0.98784353	0.70009489	0.46357649	0
2020-10-21-07-24-ADMIN	B	Wet	07	1.04661176	0.569198	0.146109	4.146253	0.62712116	0.98705651	0.63534473	0.33573407	3
2020-10-21-09-36-ADMIN	B	Wet	09	1.0665852	1.176717	0.261356	6.85631879	0.72038009	0.97940282	0.73552994	0.06666667	1
2020-11-03-08-48-ADMIN	B	Dry	08	1.032507	0.872811	0.191501	3.27256734	0.6698292	0.98966535	0.67682394	0.68446907	2
2020-11-04-06-36-ADMIN	B	Dry	06	1.05710031	0.407345	0.127634	5.10008508	0.61147441	0.97452794	0.62745703	0.02581718	5
2020-11-05-14-06-ADMIN	B	Dry	14	1.02014076	1.486504	0.33507	6.42408639	0.73419574	0.98937034	0.74208385	0.53495845	1
2020-11-06-14-30-ADMIN	B	Dry	14	1.0297982	0.715778	0.164577	2.74962027	0.67871485	0.98665615	0.68789401	0.18744229	2

2020-11-10-10-42-ADMIN	B	Dry	10	1.0306693	0.699791	0.166265	3.18209009	0.66045379	0.98924463	0.66763444	0.62089258	2
2020-11-10-11-42-ADMIN	B	Dry	11	1.03600648	0.593151	0.148189	3.2583541	0.68203543	0.98463528	0.69267824	0.10360111	1
2020-11-10-12-00-ADMIN	B	Dry	12	1.03786003	0.561277	0.145072	2.45727667	0.65206924	0.98708617	0.66060011	0.26376116	2
2020-11-11-13-00-ADMIN	B	Dry	13	1.02747568	1.617756	0.393244	7.14307305	0.75165576	0.98619296	0.76217919	0.21707602	0
2020-11-18-18-12-ADMIN	B	Dry	18	1.03513907	1.042095	0.217418	2.69748423	0.67929526	0.99058533	0.68575138	0.50293629	0
2020-11-19-11-18-ADMIN	B	Dry	11	1.03638851	0.955408	0.204472	5.60816309	0.64636635	0.99037824	0.65264595	0.87985226	3
2020-11-21-05-24-ADMIN	B	Dry	05	1.03790203	0.8528	0.185112	3.66477577	0.71082111	0.98675249	0.72036415	0.06497999	0
2020-11-23-15-00-ADMIN	B	Dry	15	1.0329524	0.648911	0.15664	2.84949228	0.66935622	0.98892984	0.67684905	0.44813789	2
2020-11-24-16-06-ADMIN	B	Dry	16	1.03161123	0.887965	0.199214	7.33374739	0.71129964	0.98762583	0.72021166	0.29311173	3
2020-11-25-10-18-ADMIN	B	Dry	10	1.02212635	1.371742	0.306601	6.00994846	0.72726415	0.98879557	0.73550507	0.55578947	1
2020-11-25-12-48-ADMIN	B	Dry	12	1.03810347	0.915768	0.198996	3.55555981	0.65659459	0.99035754	0.66298742	0.80683287	4
2020-11-26-07-18-ADMIN	B	Dry	07	1.03219726	0.970207	0.207372	3.62781159	0.71943875	0.98984396	0.72682037	0.28183441	3
2020-11-26-07-30-ADMIN	B	Dry	07	1.03356787	0.555997	0.143155	3.07703489	0.70009036	0.98847565	0.70825251	0.22850108	4
2020-11-27-05-48-ADMIN	B	Dry	05	1.03744702	0.604897	0.14924	1.82738052	0.71588594	0.98905031	0.72381145	0.0833241	1
2020-11-27-15-30-ADMIN	B	Dry	15	1.03792083	0.547915	0.144125	2.33417715	0.65939413	0.98847176	0.66708445	0.37388735	2
2020-11-30-18-12-ADMIN	B	Dry	18	1.03787804	0.551274	0.141828	2.96271719	0.68266932	0.98667203	0.69189082	0.12162512	0
2020-12-01-12-00-ADMIN	B	Dry	12	1.03340378	0.91718	0.200148	6.19790094	0.64999679	0.99015079	0.65646242	0.84333641	0
2020-12-04-18-06-ADMIN	B	Dry	18	1.03866341	0.551932	0.141866	4.33132405	0.68386511	0.98712163	0.69278708	0.18181594	0
2020-12-06-16-30-ADMIN	B	Dry	16	1.04011596	0.646389	0.158324	5.62585512	0.68903784	0.98948745	0.69635834	0.37675593	2
2020-12-06-17-30-ADMIN	B	Dry	17	1.02880113	0.567351	0.144089	5.28842048	0.69220851	0.98885393	0.70001089	0.39652816	0
2020-12-09-09-42-ADMIN	B	Dry	09	1.03487522	0.768586	0.173993	5.00066285	0.66712161	0.98680478	0.67604214	0.29742075	1
2020-12-12-05-48-ADMIN	B	Dry	05	1.03794801	0.928697	0.197534	2.31948028	0.69841682	0.98973399	0.70566114	0.27629424	1
2020-12-14-11-36-ADMIN	B	Dry	11	1.03651354	0.728444	0.164374	4.73577297	0.62968984	0.98258566	0.64084982	0.10190212	1
2020-12-20-18-48-ADMIN	B	Dry	18	1.03590427	0.963432	0.204647	2.35685838	0.6776321	0.99027718	0.68428528	0.48590951	2
2020-12-21-10-48-ADMIN	B	Dry	10	1.04156194	0.677855	0.160479	2.59052352	0.65656918	0.98931167	0.66366262	0.54879655	4
2020-12-21-10-54-ADMIN	B	Dry	10	1.04277979	0.530311	0.141331	2.54875344	0.65905572	0.98742185	0.66745102	0.29307479	5
2020-12-23-11-30-ADMIN	B	Dry	11	1.03959542	0.662099	0.158295	4.29720914	0.64358207	0.98749617	0.6517312	0.38643275	2
2020-12-25-12-00-ADMIN	B	Dry	12	1.03365588	0.678851	0.159927	2.46713161	0.69463528	0.98744368	0.70346825	0.18137273	3

2020-12-30-08-12-ADMIN	B	Dry	08	1.04211125	1.183232	0.255751	5.31473204	0.71314204	0.98946387	0.72073581	0.27535857	2
2020-12-30-13-30-ADMIN	B	Dry	13	1.036485	0.67518	0.163261	2.16932868	0.68482655	0.98892253	0.69249767	0.2842844	3
2021-01-06-12-06-ADMIN	B	Dry	12	1.03354543	0.814805	0.180886	3.29169364	0.63651189	0.98977691	0.64308622	0.74227147	0
2021-01-07-06-30-ADMIN	B	Dry	06	1.03764547	0.701898	0.161722	4.77996856	0.67928027	0.9835732	0.69062503	0.06696214	1
2021-01-12-11-54-ADMIN	B	Dry	11	1.03254332	0.562607	0.145339	2.33616019	0.67227894	0.98907754	0.67970296	0.44444444	4
2021-01-12-13-36-ADMIN	B	Dry	13	1.02989117	0.789297	0.175761	6.05326502	0.65998065	0.97874838	0.67431084	0.04033241	2
2021-01-13-14-42-ADMIN	B	Dry	14	1.02717958	0.661446	0.161	2.2412199	0.68556646	0.98901395	0.69318179	0.39869498	2
2021-01-15-13-12-ADMIN	B	Dry	13	1.02834433	0.903898	0.193565	3.06187074	0.67256622	0.98980168	0.67949593	0.60786704	4
2021-01-18-08-00-ADMIN	B	Dry	08	1.02836498	0.81695	0.178961	2.97580724	0.73089917	0.98916056	0.73890852	0.13068637	4
2021-01-23-15-54-ADMIN	B	Dry	15	1.02540905	0.872278	0.192865	1.61806943	0.72688193	0.99003887	0.73419535	0.17192983	5
2021-01-25-07-06-ADMIN	B	Dry	07	1.02940417	1.155095	0.24463	3.39548659	0.7062694	0.99040014	0.7131152	0.5313635	4
2021-02-15-11-24-ADMIN	B	Dry	11	1.03163552	0.782975	0.17349	4.27718897	0.68686159	0.98954144	0.6941211	0.60615574	5
2021-02-15-13-18-ADMIN	B	Dry	13	1.02905045	0.822291	0.180412	2.77332276	0.69460455	0.98774045	0.70322579	0.21281625	2
2021-02-15-18-12-ADMIN	B	Dry	18	1.0362167	0.56157	0.144661	4.19947825	0.69692355	0.98291018	0.70904093	0.04022161	1
2021-02-16-06-18-ADMIN	B	Dry	06	1.03756252	0.575967	0.145174	4.13759898	0.65722545	0.98220228	0.66913452	0.07069252	4
2021-02-17-07-30-ADMIN	B	Dry	07	1.03681891	1.01062	0.214018	3.15420562	0.69743202	0.98930389	0.70497248	0.40709141	4
2021-02-17-17-00-ADMIN	B	Dry	17	1.03070956	0.883692	0.19021	3.05464261	0.73028828	0.98918895	0.73826974	0.16241305	2
2021-02-18-15-24-ADMIN	B	Dry	15	1.03632361	0.829185	0.182704	4.79063798	0.6595169	0.98113448	0.67219826	0.07032318	1
2021-02-18-16-00-ADMIN	B	Dry	16	1.02842463	0.636227	0.154358	3.83305374	0.71029027	0.98687554	0.71973642	0.15204678	2
2021-02-20-07-24-ADMIN	B	Dry	07	1.02041541	0.752458	0.168367	3.4591609	0.73055695	0.98784715	0.73954452	0.13628809	4
2021-02-20-16-42-ADMIN	B	Dry	16	1.03067136	0.936648	0.203202	2.18482515	0.72689847	0.99065738	0.73375366	0.23021237	1
2021-02-20-17-12-ADMIN	B	Dry	17	1.02982759	0.98489	0.208815	2.85920018	0.71889125	0.98809985	0.7275492	0.13210219	2
2021-02-20-17-18-ADMIN	B	Dry	17	1.03098925	1.358505	0.296995	2.84382103	0.74217707	0.98938413	0.75014047	0.07571561	4
2021-02-20-17-36-ADMIN	B	Dry	17	1.03201492	1.08764	0.23306	2.07999936	0.74274144	0.99042279	0.74992362	0.10967067	3
2021-02-21-10-48-ADMIN	B	Dry	10	1.03257477	0.777941	0.174147	2.72121507	0.73597628	0.98827842	0.74470541	0.03718067	4
2021-02-22-18-42-ADMIN	B	Dry	18	1.03323572	1.11406	0.233113	2.92122617	0.71067044	0.98955102	0.71817463	0.22813173	0
2021-02-23-06-00-ADMIN	B	Dry	06	1.03581745	0.644279	0.15288	2.92909701	0.72341802	0.98135957	0.73715898	0.00523238	2
2021-02-23-13-36-ADMIN	B	Dry	13	1.04170022	0.378809	0.124093	4.72874073	0.62150389	0.97937821	0.63459027	0.0600554	3

2021-02-24-08-00-ADMIN	B	Dry	08	1.03788205	0.983707	0.209136	3.90921917	0.6792816	0.98695512	0.68825987	0.24733764	5
2021-02-25-11-18-ADMIN	B	Dry	11	1.03480956	0.765813	0.176641	2.99933854	0.67618121	0.98972609	0.68320035	0.52819945	5
2021-02-26-15-18-ADMIN	B	Dry	15	1.03014436	0.78231	0.178255	3.11012956	0.67214273	0.98948734	0.67928381	0.60576177	3
2021-02-27-08-36-ADMIN	B	Dry	08	1.03129955	0.598799	0.149324	2.88548366	0.71559129	0.98776745	0.7244532	0.13386273	4
2021-02-27-10-30-ADMIN	B	Dry	10	1.03709582	0.395671	0.126404	2.49033441	0.70242599	0.98637858	0.71212616	0.08576177	5
2021-02-28-08-06-ADMIN	B	Dry	08	1.03399859	0.557821	0.144254	2.17392361	0.70114959	0.98944581	0.70862858	0.26888273	6
2021-03-02-14-24-ADMIN	B	Dry	14	1.02970764	1.035979	0.223782	4.4879462	0.69440994	0.98846385	0.70251425	0.35791936	6
2021-03-02-15-00-ADMIN	B	Dry	15	1.02778362	0.515147	0.138816	2.7411805	0.66361353	0.98351965	0.67473337	0.08686981	5
2021-03-03-11-00-ADMIN	B	Dry	11	1.0303925	0.757633	0.172988	4.85622953	0.66492449	0.98417732	0.67561452	0.13591874	6
2021-03-03-16-24-ADMIN	B	Dry	16	1.03177331	0.58344	0.148289	2.17715096	0.67606163	0.98270493	0.68795994	0.06776239	5
2021-03-03-17-30-ADMIN	B	Dry	17	1.02875031	0.954887	0.209618	2.96792241	0.72855678	0.98901443	0.73664929	0.21181902	2
2021-03-03-18-18-ADMIN	B	Dry	18	1.03834682	0.605646	0.149687	2.70870906	0.68163231	0.98517663	0.69188843	0.09473684	3
2021-03-04-17-36-ADMIN	B	Dry	17	1.02751195	0.879754	0.195286	2.79102234	0.68730778	0.98998703	0.69425938	0.61628809	1
2021-03-05-10-42-ADMIN	B	Dry	10	1.02511774	0.757568	0.17502	3.02406831	0.68775299	0.98959137	0.69498685	0.58762696	4
2021-03-06-07-12-ADMIN	B	Dry	07	1.02357607	1.35887	0.297255	4.17175755	0.76172481	0.98910511	0.77011514	0.11052016	6
2021-03-06-10-48-ADMIN	B	Dry	10	1.02460913	1.028631	0.216846	3.47770276	0.7259798	0.98941601	0.73374576	0.27400431	5
2021-03-06-13-00-ADMIN	B	Dry	13	1.02529347	1.277993	0.275783	2.40474923	0.71905564	0.99068861	0.72581398	0.37979686	7
2021-03-06-17-12-ADMIN	B	Dry	17	1.02311861	0.743864	0.173379	2.14563651	0.69599485	0.98972668	0.70321924	0.48842105	2
2021-03-10-07-30-ADMIN	B	Dry	07	1.01689997	1.493365	0.342472	4.82313627	0.70957674	0.99046958	0.71640437	0.72754694	6
2021-03-10-13-36-ADMIN	B	Dry	13	1.01358777	1.510294	0.3456	5.61253321	0.70671302	0.99046907	0.71351347	0.82555863	3
2021-03-12-17-00-ADMIN	B	Dry	17	1.02606191	1.037853	0.220115	2.22318022	0.73448641	0.99056734	0.74148055	0.19649123	3
2021-03-13-12-06-ADMIN	B	Dry	12	1.02911689	0.856048	0.190381	2.35626533	0.72578382	0.98940912	0.7335528	0.17074792	6
2021-03-15-14-12-ADMIN	B	Dry	14	1.02649944	0.958496	0.206281	4.08544976	0.69489806	0.98379644	0.70634334	0.07623269	4
2021-03-16-08-12-ADMIN	B	Dry	08	1.01753796	1.095546	0.228163	6.02974254	0.7313923	0.98874706	0.73971628	0.4178024	3
2021-03-16-09-30-ADMIN	B	Dry	09	1.03722037	0.872343	0.192599	8.65485472	0.72151517	0.98474739	0.73269061	0.1004863	2
2021-03-16-12-18-ADMIN	B	Dry	12	1.03838514	0.58437	0.149604	3.03052907	0.65571643	0.98782143	0.66380057	0.42245614	4
2021-03-17-05-48-ADMIN	B	Dry	05	1.041481	1.180322	0.249398	5.22971932	0.73943106	0.9872863	0.74895302	0.08938135	4
2021-03-17-11-18-ADMIN	B	Dry	11	1.02428456	0.965383	0.206155	4.09830852	0.68238827	0.98825035	0.69050142	0.42105263	5

2021-03-18-10-00-ADMIN	B	Dry	10	1.03091801	0.860208	0.188114	2.88350082	0.69408621	0.98819372	0.70237869	0.24975069	5
2021-03-20-09-12-ADMIN	B	Dry	09	1.02829802	1.078783	0.230457	3.80191785	0.71925129	0.98887927	0.72733984	0.18958449	4
2021-03-22-05-48-ADMIN	B	Dry	05	1.03321769	1.259389	0.269911	3.9302874	0.71409266	0.98706174	0.72345289	0.09920591	4
2021-03-23-08-48-ADMIN	B	Dry	08	1.02800692	0.879584	0.193812	3.04057276	0.74041975	0.99012531	0.74780408	0.13739612	4
2021-03-24-10-54-ADMIN	B	Dry	10	1.03106415	0.577338	0.146194	3.01271199	0.65215907	0.98747117	0.66043353	0.36155125	4
2021-03-24-16-12-ADMIN	B	Dry	16	1.02746202	0.886017	0.193982	2.9465907	0.70258702	0.98931811	0.71017302	0.40336103	4
2021-03-25-13-00-ADMIN	B	Dry	13	1.02105003	0.933416	0.204616	5.01694711	0.69571023	0.98704118	0.70484418	0.24992305	4
2021-03-27-05-30-ADMIN	B	Dry	05	1.03244525	1.127486	0.236987	2.80415701	0.75469762	0.98731501	0.76439395	0.01229917	3
2021-03-27-14-00-ADMIN	B	Dry	14	1.02387002	0.674935	0.162067	2.50723062	0.71241618	0.98920636	0.72018965	0.28473992	6
2021-03-27-18-30-ADMIN	B	Dry	18	1.02637637	0.859745	0.188375	2.16682949	0.72322248	0.98987535	0.73061975	0.23734072	2
2021-03-28-18-54-ADMIN	B	Dry	18	1.02809573	0.623609	0.152851	1.82013302	0.7012293	0.9880959	0.70967737	0.16842105	0
2021-03-31-05-06-ADMIN	B	Dry	05	1.03450743	0.765254	0.169512	1.62436469	0.75706802	0.98829691	0.76603297	0.00748538	1
2021-03-31-12-12-ADMIN	B	Dry	12	1.02865546	0.754965	0.172695	1.58434359	0.73908879	0.98954324	0.74689893	0.09307479	6
2021-04-07-17-00-ADMIN	B	Dry	17	1.03197028	0.980319	0.212561	3.04950731	0.70210056	0.98634477	0.71182063	0.12606956	4
2021-04-08-11-30-ADMIN	B	Dry	11	1.0278387	1.096134	0.23537	3.33419566	0.71570834	0.98954486	0.72327023	0.37402278	6
2021-04-08-14-48-ADMIN	B	Dry	14	1.02341484	0.793912	0.182135	1.7849612	0.73271926	0.99072753	0.73957697	0.21926747	6
2021-04-08-16-06-ADMIN	B	Dry	16	1.03163095	0.702277	0.163755	3.15949614	0.6822577	0.98544055	0.69233776	0.12899969	3
2021-04-10-06-24-ADMIN	B	Dry	06	1.02787236	0.873868	0.192418	3.44377804	0.76584109	0.98555763	0.77706374	0.00465374	4
2021-04-10-12-48-ADMIN	B	Dry	12	1.02273959	1.6534	0.409867	2.59305335	0.7838913	0.98980972	0.79196161	0.01446599	3
2021-04-12-10-18-ADMIN	B	Dry	10	1.02361568	1.276777	0.281717	2.07376679	0.7254646	0.99059578	0.7323518	0.33324715	5
2021-04-12-11-06-ADMIN	B	Dry	11	1.01908794	1.153773	0.255785	2.15504821	0.72289617	0.99061054	0.72974811	0.37754386	4
2021-04-12-15-00-ADMIN	B	Dry	15	1.02089423	1.129981	0.248468	2.49618362	0.76489571	0.98959244	0.77294014	0.05765466	5
2021-04-15-06-24-ADMIN	B	Dry	06	1.02594062	0.858651	0.187278	2.41917148	0.75478986	0.98939555	0.76287978	0.03804248	3
2021-04-15-10-12-ADMIN	B	Dry	10	1.0305946	1.024388	0.218321	4.03044964	0.6895952	0.98566069	0.69962737	0.14930132	4
2021-04-16-05-36-ADMIN	B	Dry	05	1.03911154	1.994047	0.58263	5.03280633	0.79121129	0.98955971	0.79955892	0.02979378	6
2021-04-16-14-06-ADMIN	B	Dry	14	1.02461442	0.803575	0.179218	3.23798253	0.72112171	0.98818268	0.72974534	0.21929209	2
2021-04-17-09-24-ADMIN	B	Dry	09	1.03663713	1.179506	0.254997	4.75727307	0.73985098	0.98795767	0.74886911	0.14168052	4
2021-04-18-12-54-ADMIN	B	Dry	12	1.02521661	1.487708	0.334761	3.58550659	0.79362378	0.98564667	0.80518081	0.00162512	5

2021-04-20-14-54-ADMIN	B	Dry	14	1.04026373	0.50289	0.13977	3.41553376	0.59369806	0.98346085	0.60368245	0.12151431	2
2021-04-21-10-48-ADMIN	B	Dry	10	1.0167081	1.264533	0.271276	5.03623557	0.74059792	0.98658332	0.75066942	0.22869806	2
2021-04-21-11-18-ADMIN	B	Dry	11	1.02988329	0.588386	0.147584	4.39189239	0.7046928	0.97892763	0.719862	0.02566944	4
2021-05-21-14-00-ADMIN	B	Wet	14	1.02250069	0.939661	0.199917	4.76929343	0.69032686	0.98378281	0.70170657	0.11912589	0
2021-05-22-13-06-ADMIN	B	Wet	13	1.02552077	0.401389	0.137714	6.82492705	0.74816449	0.97913072	0.76411093	0.04060326	0
2021-05-23-09-00-ADMIN	B	Wet	09	1.018897	1.663771	0.412701	7.68710921	0.81885925	0.9865053	0.83006067	0.06308403	1
2021-05-23-09-24-ADMIN	B	Wet	09	1.02171578	1.652379	0.417709	6.75642102	0.79113891	0.98608672	0.80230155	0.09292705	3
2021-05-23-18-18-ADMIN	B	Wet	18	1.03111898	1.562808	0.396197	4.30131353	0.73486757	0.98914438	0.74293257	0.21037858	2
2021-06-25-16-42-ADMIN	B	Wet	16	1.03171215	0.986572	0.208874	3.20786513	0.72727802	0.98820468	0.73595889	0.10203755	1
2021-06-26-06-12-ADMIN	B	Wet	06	1.02915513	1.69415	0.42198	6.16618875	0.80315546	0.98892816	0.81214743	0.09750693	4
2021-06-26-15-00-ADMIN	B	Wet	15	1.03395616	0.717852	0.166392	4.24888703	0.70918995	0.98712742	0.71843811	0.11634349	1
2021-06-26-16-54-ADMIN	B	Wet	16	1.03340051	1.839892	0.497509	4.01143699	0.77150736	0.98979076	0.77946511	0.0628378	4
2021-06-28-10-36-ADMIN	B	Wet	10	1.028952	0.082264	0.103209	4.36642946	0.61564697	0.97980989	0.62833308	0.06773777	3
2021-06-28-13-24-ADMIN	B	Wet	13	1.02522299	0.068512	0.10256	4.94732422	0.57734189	0.97237958	0.59374127	0.02747922	0
2021-06-28-15-12-ADMIN	B	Wet	15	1.02849458	0.128201	0.105603	6.76471679	0.6956104	0.9873982	0.70448822	0.31580179	2
2021-06-28-18-18-ADMIN	B	Wet	18	1.03115437	0.066616	0.102581	4.06948239	0.68348189	0.98475124	0.69406553	0.10038781	4
2021-06-29-07-30-ADMIN	B	Wet	07	1.02041242	0.486555	0.135566	7.59734431	0.80002811	0.98372225	0.81326625	0.06549708	3
2021-06-29-13-00-ADMIN	B	Wet	13	1.03030912	0.108958	0.104554	4.78571719	0.72212864	0.98530676	0.73289728	0.07844875	4
2021-07-02-08-06-ADMIN	B	Wet	08	1.03105469	1.761058	0.457852	5.66659465	0.71394765	0.98670907	0.7235645	0.27146814	3
2021-07-02-09-36-ADMIN	B	Wet	09	1.02477547	1.72612	0.435274	7.82224401	0.75602702	0.9796621	0.77172223	0.06008003	2
2021-07-02-09-48-ADMIN	B	Wet	09	1.01712348	1.564763	0.385292	6.85381666	0.75743615	0.97893559	0.77373441	0.04767005	3
2021-07-03-18-24-ADMIN	B	Wet	18	1.03676322	0.862429	0.185075	6.39334778	0.70247357	0.95923403	0.73232762	0.00380425	3
2021-07-04-14-12-ADMIN	B	Wet	14	1.0258985	0.863247	0.208343	5.94111551	0.74264937	0.98847599	0.75130744	0.41625115	1
2021-07-05-12-42-ADMIN	B	Wet	12	1.0538015	0.931905	0.198299	9.05381475	0.68799707	0.95155491	0.72302403	0.00220376	2
2021-07-05-17-36-ADMIN	B	Wet	17	1.02881582	1.380246	0.30555	5.94238793	0.70652957	0.98995587	0.71369805	0.74770083	2
2021-07-07-12-48-ADMIN	B	Wet	12	1.03283857	0.680786	0.161172	3.98222901	0.64984272	0.99086073	0.65583659	0.91074177	2
2021-07-09-08-18-ADMIN	B	Wet	08	1.01875961	2.044307	0.604912	6.85386438	0.73934433	0.98870861	0.74778789	0.50683903	2
2021-07-10-06-12-ADMIN	B	Wet	06	1.03048352	0.681169	0.162434	5.14320321	0.71569022	0.98149764	0.72918181	0.05537704	2

2021-07-10-07-24-ADMIN	B	Wet	07	1.01722074	1.86328	0.509767	5.72498337	0.76392508	0.98869924	0.77265668	0.23219452	3
2021-07-11-06-06-ADMIN	B	Wet	06	1.03483718	1.306274	0.285271	3.48185221	0.73062004	0.98903231	0.73872212	0.16345953	4
2021-07-13-16-24-ADMIN	B	Wet	16	1.02148809	0.683015	0.162194	2.71619817	0.6903388	0.98947176	0.69768419	0.48285626	4
2021-07-13-17-00-ADMIN	B	Wet	17	1.01249161	1.58942	0.382432	9.55523484	0.7662874	0.99109414	0.77317317	0.90877193	2
2021-07-15-05-36-ADMIN	B	Wet	05	1.02506878	0.330341	0.120947	7.17095912	0.76099851	0.9875245	0.77061228	0.15512465	3
2021-07-17-10-00-ADMIN	B	Wet	10	1.01774161	0.627085	0.161895	7.04237105	0.76856654	0.98858098	0.77744419	0.36299169	2
2021-07-17-12-36-ADMIN	B	Wet	12	1.02950508	0.003088	0.100069	2.78436642	0.70098966	0.9874133	0.70992527	0.17263158	4
2021-07-17-18-30-ADMIN	B	Wet	18	1.02014807	0.754849	0.182667	8.76049585	0.76574842	0.9865472	0.77619035	0.25429363	0
2021-07-18-10-30-ADMIN	B	Wet	10	1.03142999	0.192247	0.109565	2.89588767	0.70627197	0.98454391	0.71735954	0.04550323	3
2021-07-18-12-06-ADMIN	B	Wet	12	1.0324551	0.027666	0.100867	2.61514669	0.69712365	0.98530294	0.70752214	0.06569406	3
2021-07-18-14-30-ADMIN	B	Wet	14	1.03142343	0.054299	0.101922	4.97453865	0.56600346	0.96422972	0.58700063	0.01280394	2
2021-07-18-15-00-ADMIN	B	Wet	15	1.03171795	0.018438	0.100532	3.0962917	0.58353727	0.96753921	0.60311485	0.01602955	2
2021-07-20-09-00-ADMIN	B	Wet	09	1.03504141	0.166739	0.109046	4.27096315	0.69794882	0.98627465	0.70766172	0.08558941	5
2021-07-20-16-54-ADMIN	B	Wet	16	1.01542067	0.663245	0.157724	8.79635104	0.73496273	0.98012459	0.74986664	0.05660819	1
2021-07-21-11-36-ADMIN	B	Wet	11	1.01635021	0.573961	0.151776	6.27881647	0.68908056	0.98831327	0.69722889	0.5198892	0
2021-07-21-15-00-ADMIN	B	Wet	15	1.0344414	0.034257	0.101106	2.16280237	0.65676963	0.98539286	0.66650537	0.10889505	2
2021-07-23-05-42-ADMIN	B	Wet	05	1.03268902	0.011329	0.100372	4.87854797	0.73399636	0.98331335	0.74645215	0.0171622	3
2021-07-24-10-42-ADMIN	B	Wet	10	1.02568691	0.208499	0.110646	6.24359846	0.72537677	0.9878039	0.73433277	0.35752539	3
2021-07-24-13-18-ADMIN	B	Wet	13	1.02663336	0.072785	0.102759	4.36693804	0.67723148	0.98591578	0.68690601	0.18603878	4
2021-07-26-16-00-ADMIN	B	Wet	16	1.02953647	0.80598	0.199011	9.32484422	0.70020858	0.98102034	0.71375542	0.07871961	1
2021-07-27-18-00-ADMIN	B	Wet	18	1.04544472	0.145735	0.107216	3.48549718	0.62761228	0.97745739	0.64208659	0.03825177	2
2021-07-29-11-30-ADMIN	B	Wet	11	1.0381292	0.462761	0.133179	3.53537977	0.62911357	0.98601795	0.6380346	0.22989228	3
2021-07-30-09-00-ADMIN	B	Wet	09	1.02469006	1.279707	0.279741	5.56553499	0.69126831	0.98960875	0.69852687	0.68944291	2
2021-08-04-06-54-ADMIN	B	Wet	06	1.03476054	0.296656	0.117306	3.76682753	0.66393547	0.98505047	0.67401163	0.1461619	5
2021-08-04-18-30-ADMIN	B	Wet	18	1.03356962	0.457888	0.133906	7.81236326	0.61163417	0.9657022	0.63335692	0.01649738	0
2021-08-05-16-00-ADMIN	B	Wet	16	1.03195693	1.171147	0.268307	4.95130547	0.68672396	0.98172449	0.69950782	0.09376424	2
2021-08-06-08-24-ADMIN	B	Wet	08	1.0338576	0.731	0.172483	6.0289433	0.68488318	0.97637357	0.70145609	0.03426285	3
2021-08-06-13-18-ADMIN	B	Wet	13	1.04752148	0.347578	0.121369	6.46615913	0.5998805	0.95736873	0.62659296	0.00689443	0

2021-08-07-17-24-ADMIN	B	Wet	17	1.02580002	0.547831	0.141371	4.98313469	0.69626042	0.98381665	0.7077136	0.10184057	2
2021-08-09-06-06-ADMIN	B	Wet	06	1.02758745	1.890285	0.507781	3.74583823	0.76939318	0.99034238	0.77689615	0.05988304	4
2021-08-10-16-18-ADMIN	B	Wet	16	1.02910787	1.255745	0.273568	7.77304591	0.72824522	0.98764916	0.73735214	0.27239151	2
2021-08-12-08-18-ADMIN	B	Wet	08	1.02692332	0.666632	0.158441	2.96621759	0.71586312	0.98892836	0.72387764	0.21392428	2
2021-08-12-09-18-ADMIN	B	Wet	09	1.02782251	0.870975	0.188777	3.15002707	0.70555364	0.9867843	0.7150029	0.12594645	3
2021-08-15-08-48-ADMIN	B	Wet	08	1.03229898	0.496126	0.136227	2.79145643	0.7516118	0.98562147	0.76257653	0.00560172	5
2021-08-16-10-30-ADMIN	B	Wet	10	1.032817	0.720733	0.168911	2.62032649	0.6863588	0.98975623	0.69346247	0.4473992	5
2021-08-17-08-48-ADMIN	B	Wet	08	1.02872837	1.460575	0.332124	8.20080279	0.72664911	0.98394471	0.73850604	0.12520776	1
2021-08-17-14-30-ADMIN	B	Wet	14	1.02505102	0.927579	0.198583	2.90682137	0.69905932	0.99007178	0.70606933	0.47134503	2
2021-08-19-16-42-ADMIN	B	Wet	16	1.03097559	1.033154	0.215634	3.08233728	0.7261261	0.98874396	0.73439245	0.10074485	1
2021-08-21-11-36-ADMIN	B	Wet	11	1.03910383	0.414393	0.12864	1.69108837	0.6599872	0.98456115	0.67033642	0.09169591	3
2021-08-21-17-48-ADMIN	B	Wet	17	1.03077395	0.950171	0.201923	3.29980718	0.68002344	0.99041786	0.68660257	0.63944598	1
2021-08-21-18-00-ADMIN	B	Wet	18	1.03067839	1.352384	0.29526	5.87986938	0.7069881	0.9860322	0.71700306	0.193241	2
2021-08-22-07-24-ADMIN	B	Wet	07	1.02902162	0.644227	0.154932	2.40964304	0.73556142	0.98876313	0.74392076	0.07953216	4
2021-08-22-15-00-ADMIN	B	Wet	15	1.02292669	1.183491	0.267877	7.45500286	0.77479766	0.98802609	0.78418745	0.13540166	1
2021-08-23-05-18-ADMIN	B	Wet	05	1.030054	0.487597	0.134573	3.90669006	0.69156624	0.97688919	0.70792701	0.01728532	1
2021-08-24-07-12-ADMIN	B	Wet	07	1.02677245	0.454835	0.131463	3.8460909	0.68937176	0.98370065	0.70079425	0.06530009	4
2021-08-24-14-30-ADMIN	B	Wet	14	1.05096643	0.731624	0.168642	8.91406626	0.67779711	0.98379102	0.68896452	0.12254848	3
2021-08-25-09-54-ADMIN	B	Wet	09	1.03280368	0.745912	0.167462	4.11207798	0.69282972	0.98773073	0.70143583	0.23322869	2
2021-08-26-13-00-ADMIN	B	Wet	13	1.03640144	0.678081	0.163894	1.9350125	0.65996589	0.99043446	0.66633979	0.7188181	4
2021-08-28-10-06-ADMIN	B	Wet	10	1.03175244	1.056222	0.221057	3.9343623	0.71019119	0.98749677	0.7191833	0.19338874	1
2021-08-28-15-06-ADMIN	B	Wet	15	1.02421616	1.415997	0.313423	6.37249594	0.73034657	0.99001704	0.73771111	0.58578024	1
2021-08-29-09-42-ADMIN	B	Wet	09	1.03017977	0.671481	0.160338	1.96098568	0.72709639	0.98842104	0.73561404	0.06145891	4
2021-08-29-10-30-ADMIN	B	Wet	10	1.03763541	0.592291	0.148251	2.85438442	0.6890412	0.98700295	0.69811464	0.14504155	3
2021-08-31-18-42-ADMIN	B	Wet	18	1.03164529	0.858891	0.186414	3.43388653	0.68227563	0.98901072	0.68985666	0.38526316	0
2021-09-02-06-00-ADMIN	B	Wet	06	1.0368809	1.300301	0.284786	3.19735274	0.72737058	0.98723491	0.73677559	0.06718375	2
2021-09-03-05-00-ADMIN	B	Wet	05	1.03227436	0.493536	0.136631	2.45310076	0.73609587	0.98367914	0.74830891	0.00748538	0
2021-09-03-07-06-ADMIN	B	Wet	07	1.03103297	0.747048	0.169149	2.86120819	0.69768783	0.98258526	0.71005323	0.03693444	4

2021-09-03-12-24-ADMIN	B	Wet	12	1.03233258	0.868539	0.189369	2.66186253	0.69769742	0.98836288	0.7059122	0.2137273	2
2021-09-04-09-54-ADMIN	B	Wet	09	1.03146125	0.556096	0.145527	2.04941808	0.68312118	0.98881642	0.69084733	0.34060942	1
2021-09-04-13-06-ADMIN	B	Wet	13	1.03440246	0.284105	0.116226	2.68613626	0.67436803	0.98559622	0.68422343	0.12606956	2
2021-09-05-06-18-ADMIN	B	Wet	06	1.02975474	1.078234	0.230619	3.28514714	0.74036453	0.987256	0.74992153	0.03058172	4
2021-09-05-13-00-ADMIN	B	Wet	13	1.03160011	0.782765	0.177363	1.49321399	0.74261607	0.98905568	0.75083343	0.03024931	1
2021-09-05-14-00-ADMIN	B	Wet	14	1.02089642	2.118761	0.663317	8.69191663	0.77279376	0.986393	0.78345422	0.16960296	1
2021-09-06-12-36-ADMIN	B	Wet	12	1.0376025	0.579642	0.14518	3.70781653	0.66528961	0.98606257	0.6746931	0.18334257	2
2021-09-06-13-36-ADMIN	B	Wet	13	1.02623841	1.430922	0.3256	7.55238135	0.75942191	0.98617986	0.77006431	0.11449677	1
2021-09-07-14-00-ADMIN	B	Wet	14	1.02630435	1.15372	0.248167	2.76536588	0.69257855	0.99014843	0.69946943	0.49355494	3
2021-09-07-15-06-ADMIN	B	Wet	15	1.05311208	0.391921	0.12518	3.41565636	0.60993998	0.96865509	0.62967716	0.01663281	2
2021-09-08-07-42-ADMIN	B	Wet	07	1.02623879	1.887019	0.524801	6.69951695	0.77322008	0.99100356	0.78023946	0.52097261	0
2021-09-08-11-54-ADMIN	B	Wet	11	1.02346341	1.720195	0.450541	6.83729368	0.7627291	0.99093968	0.76970285	0.64923361	2
2021-09-09-15-12-ADMIN	B	Wet	15	1.06318603	0.890783	0.19261	5.6969391	0.70529762	0.98728475	0.71438115	0.35561711	1
2021-09-09-15-36-ADMIN	B	Wet	15	1.02163021	1.205663	0.26871	7.69486	0.7244631	0.98870125	0.73274217	0.50581718	1
2021-09-10-05-30-ADMIN	B	Wet	05	1.03316948	1.013245	0.21622	4.92577667	0.72475673	0.98823132	0.73338774	0.1724346	2
2021-09-10-07-00-ADMIN	B	Wet	07	1.03004978	0.929458	0.203771	3.97743592	0.73124973	0.9903325	0.7383881	0.23859649	3
2021-09-10-09-42-ADMIN	B	Wet	09	1.02789432	0.464818	0.134685	3.52324684	0.66685306	0.98625919	0.67614382	0.22086796	4
2021-09-11-06-06-ADMIN	B	Wet	06	1.03597035	1.163271	0.248617	5.41416733	0.71513162	0.99017129	0.72223021	0.45296399	2
2021-09-11-08-12-ADMIN	B	Wet	08	1.03208121	0.895625	0.194787	3.14477056	0.67700877	0.99098365	0.68316845	0.70031394	4
2021-11-27-15-42-ADMIN	B	Dry	15	1.0302217	0.59647	0.150167	2.50236765	0.69505417	0.98768201	0.70372262	0.18770083	2
2021-11-29-14-24-ADMIN	B	Dry	14	1.00496266	2.205131	0.751178	6.09189565	0.85459475	0.99004947	0.86318389	0.68144044	1
2021-11-29-15-00-ADMIN	B	Dry	15	1.02775506	0.713425	0.164638	2.46460947	0.69602109	0.98875657	0.70393575	0.35370883	3
2021-12-01-18-24-ADMIN	B	Dry	18	1.03060283	1.1428	0.245552	2.30903412	0.72572075	0.99065105	0.7325695	0.20609418	0
2021-12-03-17-18-ADMIN	B	Dry	17	1.0343281	0.694638	0.162841	2.93716712	0.69816442	0.98860245	0.70621352	0.27492767	2
2021-12-04-08-00-ADMIN	B	Dry	08	1.02683816	1.045255	0.223992	3.16175229	0.71912629	0.98598236	0.72935006	0.07842413	1
2021-12-05-05-36-ADMIN	B	Dry	05	1.03219162	0.773761	0.17212	2.05260666	0.7163713	0.98539143	0.72699162	0.03102493	0
2021-12-05-08-12-ADMIN	B	Dry	08	1.0249383	0.723051	0.166117	2.00280646	0.74282669	0.98805979	0.75180338	0.05457679	3
2021-12-07-12-06-ADMIN	B	Dry	12	1.02833201	0.962085	0.205635	3.39815487	0.67627765	0.98879794	0.68393918	0.45316097	2

2021-12-09-16-36-ADMIN	B	Dry	16	1.02497145	0.852646	0.1859	4.47045243	0.70886983	0.98752697	0.71782326	0.29346876	1
2021-12-12-16-18-ADMIN	B	Dry	16	1.02631027	0.666533	0.162643	1.93139208	0.75524525	0.98955839	0.76321443	0.03235457	1
2021-12-13-07-18-ADMIN	B	Dry	07	1.02419405	1.007047	0.213005	3.34897127	0.7375587	0.98903096	0.74573874	0.17174515	2
2021-12-13-13-42-ADMIN	B	Dry	13	1.0262757	0.883035	0.192313	4.74978341	0.71305945	0.98690677	0.72251957	0.1934626	1
2021-12-29-06-54-ADMIN	B	Dry	06	1.02478392	0.928411	0.199097	2.93854979	0.7507059	0.98942145	0.75873218	0.08491228	4
2021-12-30-05-18-ADMIN	B	Dry	05	1.03548124	1.381406	0.305319	1.76422391	0.78700088	0.98884157	0.79588167	0.00215451	0
2021-12-30-13-00-ADMIN	B	Dry	13	1.03208443	0.620467	0.152666	1.83531406	0.7243484	0.98843323	0.73282482	0.08891351	2
2021-12-30-15-54-ADMIN	B	Dry	15	1.02566869	0.647195	0.156771	2.21814528	0.73315019	0.99032396	0.74031349	0.20013543	1
2021-12-31-16-48-ADMIN	B	Dry	16	1.02871943	0.857069	0.192281	2.4969139	0.68461793	0.98880736	0.69236735	0.3833795	3
2022-01-03-07-30-ADMIN	B	Dry	07	1.0201835	1.700434	0.420083	2.38982398	0.75560478	0.98997085	0.76325963	0.12811327	4
2022-01-03-11-06-ADMIN	B	Dry	11	1.02221276	0.961421	0.206782	2.20431427	0.73910871	0.98960965	0.74686894	0.17451524	2
2022-01-03-16-24-ADMIN	B	Dry	16	1.02434897	0.950516	0.207998	2.15757022	0.73032547	0.98801147	0.73918724	0.12409972	2
2022-01-05-08-12-ADMIN	B	Dry	08	1.030777	0.493556	0.136386	2.16307915	0.72653657	0.98487943	0.73769088	0.03385657	5
2022-01-06-09-18-ADMIN	B	Dry	09	1.02135857	1.423318	0.314268	3.37664938	0.72736685	0.98985453	0.73482196	0.3501385	3
2022-01-06-14-36-ADMIN	B	Dry	14	1.02715547	0.948193	0.205685	1.89478737	0.73226296	0.99039538	0.73936427	0.23277316	3
2022-01-09-11-54-ADMIN	B	Dry	11	1.03242677	0.781422	0.176275	1.86263688	0.73851809	0.98979559	0.74613192	0.11187442	2
2022-01-10-05-00-ADMIN	B	Dry	05	1.02849047	0.525281	0.141761	1.8825695	0.73771975	0.98786507	0.74678189	0.0301385	0
2022-01-11-06-42-ADMIN	B	Dry	06	1.01909748	1.413657	0.33906	3.33237096	0.80332096	0.98799745	0.81308	0.01711296	4
2022-01-12-11-36-ADMIN	B	Dry	11	1.02412193	0.552166	0.142716	1.83438593	0.72599927	0.98752913	0.73516745	0.08110803	2
2022-01-14-05-36-ADMIN	B	Dry	05	1.0325641	1.158061	0.244529	1.70098242	0.75674704	0.98931537	0.76491992	0.01969837	0
2022-01-15-05-00-ADMIN	B	Dry	05	1.03710836	0.457934	0.132927	1.37893018	0.7563992	0.9879625	0.7656153	0.00509695	0
2022-01-18-17-18-ADMIN	B	Dry	17	1.0251631	0.87505	0.192983	2.29351924	0.73087791	0.98771417	0.73996904	0.08286858	1
2022-01-19-06-30-ADMIN	B	Dry	06	1.03342991	0.847059	0.184135	2.18497863	0.74702424	0.98833946	0.75583772	0.02654355	3
2022-01-20-18-00-ADMIN	B	Dry	18	1.03325637	0.725354	0.167064	1.72830148	0.73233943	0.9898809	0.73982581	0.10887042	0
2022-01-22-08-24-ADMIN	B	Dry	08	1.02599315	0.939456	0.200585	2.43808037	0.74528342	0.98991213	0.75287835	0.11819021	3
2022-01-23-06-12-ADMIN	B	Dry	06	1.03134902	1.240989	0.263252	2.28190368	0.72088416	0.9893497	0.72864445	0.17408433	1
2022-01-26-14-36-ADMIN	B	Dry	14	1.02870219	0.649274	0.160025	2.20171241	0.69988689	0.99015401	0.7068465	0.43571561	1
2022-01-26-15-36-ADMIN	B	Dry	15	1.01899851	1.475345	0.339749	6.43160045	0.76396795	0.98935569	0.77218736	0.39237919	0

2022-01-27-12-00-ADMIN	B	Dry	12	1.02777207	0.590345	0.152334	2.84600515	0.67540926	0.98804005	0.6835849	0.38635888	2
2022-01-28-11-12-ADMIN	B	Dry	11	1.02916961	0.880281	0.195047	2.53727763	0.67071226	0.9906761	0.67702477	0.78694983	2
2022-01-30-06-06-ADMIN	B	Dry	06	1.02973269	1.670944	0.399874	2.84526911	0.74956138	0.99090774	0.75643912	0.0908618	2
2020-12-18-12-24-CSLIB	C	Dry	12	1.04032626	0.284508	0.12261	9.90414426	0.51058011	0.97654247	0.52284476	0.18885811	3
2020-12-19-15-06-CSLIB	C	Dry	15	1.00862776	0.609107	0.16581	10.0146191	0.547385	0.98447608	0.55601656	0.47679902	1
2020-12-20-15-06-CSLIB	C	Dry	15	1.06037629	0.014878	0.10047	10.6517676	0.38497826	0.95764691	0.40200438	0.05207756	3
2020-12-20-17-42-CSLIB	C	Dry	17	1.02234097	0.112763	0.106486	8.73981725	0.42280456	0.98539298	0.42907203	0.55578947	1
2020-12-22-12-48-CSLIB	C	Dry	12	1.07629623	0.072344	0.104025	8.50367514	0.35312636	0.97847531	0.36089451	0.25927978	3
2020-12-22-14-00-CSLIB	C	Dry	14	1.02145415	0.184408	0.10897	8.20471891	0.38338731	0.98385598	0.38967829	0.43951985	1
2020-12-23-11-12-CSLIB	C	Dry	11	1.03792744	0.290788	0.131203	20.3396441	0.42058435	0.979386	0.42943676	0.29328409	3
2020-12-23-12-24-CSLIB	C	Dry	12	1.049024	0.042151	0.101803	11.9317414	0.38630896	0.97572211	0.39592109	0.18009234	4
2020-12-27-05-18-CSLIB	C	Dry	05	0.99663219	0.152195	0.110639	8.20174553	0.5194062	0.98230938	0.52876029	0.08204371	1
2020-12-27-09-18-CSLIB	C	Dry	09	1.00539145	1.997574	0.587358	7.25295549	0.81208031	0.98467811	0.82471653	0.54976916	3
2020-12-27-15-06-CSLIB	C	Dry	15	1.01416347	1.00128	0.242803	9.14896425	0.5119392	0.98754864	0.51839391	0.33868883	3
2020-12-27-16-06-CSLIB	C	Dry	16	1.01207347	0.397409	0.140044	9.05087252	0.50804327	0.98068245	0.51805074	0.13185596	4
2020-12-30-05-48-CSLIB	C	Dry	05	1.03605448	0.711279	0.166034	14.5878114	0.53285562	0.98345586	0.54181956	0.1767313	1
2021-01-04-18-00-CSLIB	C	Dry	18	0.9714184	0.16126	0.110425	9.86079458	0.46639639	0.98243766	0.47473382	0.26674054	1
2021-01-07-06-54-CSLIB	C	Dry	06	1.08389119	0.919044	0.214478	15.4941573	0.54693928	0.98260158	0.55662365	0.42307171	5
2021-01-13-05-42-CSLIB	C	Dry	05	1.00497661	0.109914	0.104664	8.50612644	0.48532219	0.98208708	0.49417429	0.14697445	1
2021-01-14-16-48-CSLIB	C	Dry	16	1.00019938	0.215508	0.111018	11.2735488	0.44746889	0.9797682	0.45670894	0.22049862	3
2021-01-16-06-54-CSLIB	C	Dry	06	1.08960391	0.953984	0.226643	14.3796577	0.57283664	0.98258488	0.58298947	0.21698984	6
2021-01-18-15-30-CSLIB	C	Dry	15	0.99826357	0.276956	0.11653	8.75126242	0.4787349	0.98830488	0.48440002	0.37016928	2
2021-01-20-14-54-CSLIB	C	Dry	14	0.97580605	0.072282	0.103013	7.79328321	0.37401789	0.9827457	0.38058461	0.2714189	2
2021-01-26-13-30-CSLIB	C	Dry	13	1.03498764	0.153762	0.107322	10.4385399	0.41779731	0.98632144	0.42359143	0.69978455	6
2021-01-26-14-36-CSLIB	C	Dry	14	1.01530318	0.619609	0.181276	10.2452062	0.45170195	0.98465378	0.4587419	0.48007387	4
2021-01-28-09-18-CSLIB	C	Dry	09	1.05891986	1.306195	0.323504	15.4241562	0.66776046	0.98158208	0.68028998	0.15364728	5
2021-01-28-14-30-CSLIB	C	Dry	14	0.97467691	0.073513	0.103012	4.07655346	0.48512833	0.972096	0.49905393	0.10944906	2
2021-01-30-07-00-CSLIB	C	Dry	07	1.01065345	0.792789	0.199044	6.56695588	0.52562799	0.98107888	0.53576527	0.18890736	7

2021-01-31-07-42-CSLIB	C	Dry	07	0.99275278	0.778165	0.185309	15.0976091	0.53007074	0.98460561	0.53835843	0.14108957	4
2021-02-01-12-12-CSLIB	C	Dry	12	1.02847827	0.062858	0.102303	6.52964359	0.40951833	0.98118267	0.41737216	0.27368421	4
2021-02-02-08-54-CSLIB	C	Dry	08	1.01722607	0.462828	0.139461	10.9675848	0.49495011	0.98302183	0.5034986	0.35601108	7
2021-02-02-13-30-CSLIB	C	Dry	13	0.99145288	0.221411	0.111776	9.05396228	0.43569497	0.98398215	0.44278747	0.40467836	3
2021-02-03-08-18-CSLIB	C	Dry	08	1.11871776	1.095247	0.252804	11.4315801	0.61973824	0.98272382	0.63063317	0.19458295	5
2021-02-03-10-00-CSLIB	C	Dry	10	1.05686918	0.52745	0.149529	14.7391699	0.52178814	0.9687288	0.53863181	0.06955987	3
2021-02-05-05-18-CSLIB	C	Dry	05	0.99455915	0.02581	0.100958	10.9293586	0.44393858	0.98342534	0.45142073	0.15157895	0
2021-02-05-17-54-CSLIB	C	Dry	17	0.97609332	0.250393	0.1137	9.83507261	0.43516914	0.98692169	0.44093583	0.36195753	1
2021-02-06-14-00-CSLIB	C	Dry	14	0.98858495	0.109381	0.105103	12.7100077	0.41409452	0.9829802	0.42126436	0.34578024	3
2021-02-06-15-54-CSLIB	C	Dry	15	1.0078648	0.073816	0.103906	14.1897501	0.41355681	0.97908159	0.42239259	0.23165282	2
2021-02-07-12-00-CSLIB	C	Dry	12	1.06131512	0.031738	0.101058	11.7337984	0.37951979	0.9373892	0.40486896	0.01827024	6
2021-02-10-10-42-CSLIB	C	Dry	10	1.00084988	0.278103	0.119864	8.13067927	0.44546544	0.98156819	0.45383035	0.31791936	5
2021-02-10-17-06-CSLIB	C	Dry	17	1.02677116	0.075467	0.103109	9.67605064	0.41391251	0.98473535	0.42032868	0.39533395	2
2021-02-12-14-00-CSLIB	C	Dry	14	1.01353566	0.040312	0.101842	10.474462	0.38529195	0.98426411	0.39145179	0.12631579	2
2021-02-13-07-06-CSLIB	C	Dry	07	1.01755339	0.404947	0.136535	14.6294459	0.45796839	0.98608399	0.46443142	0.23093875	3
2021-02-13-14-36-CSLIB	C	Dry	14	0.98120725	0.10763	0.104539	7.18546006	0.38074654	0.98413809	0.38688325	0.45460142	3
2021-02-14-10-54-CSLIB	C	Dry	10	0.96520064	0.115357	0.106744	10.2505946	0.40260885	0.98216209	0.40992098	0.05830717	5
2021-02-16-13-06-CSLIB	C	Dry	13	1.01253584	0.05591	0.102095	9.69529202	0.38529878	0.97794293	0.39398902	0.21514312	3
2021-02-18-11-54-CSLIB	C	Dry	11	0.9923724	0.168802	0.110367	6.81365509	0.42707093	0.9829963	0.43445833	0.34245614	3
2021-02-20-09-30-CSLIB	C	Dry	09	1.01018012	0.19535	0.11103	13.2628499	0.42951369	0.98249199	0.43716763	0.20022161	7
2021-02-21-06-30-CSLIB	C	Dry	06	0.99803725	0.518321	0.154902	8.20827337	0.49824846	0.98761179	0.50449829	0.08742382	5
2021-02-22-05-06-CSLIB	C	Dry	05	1.00669637	0.723314	0.167312	6.25122415	0.51901227	0.98608379	0.52633689	0.22714681	3
2021-02-23-05-42-CSLIB	C	Dry	05	0.99470573	0.561567	0.15644	10.9411753	0.54227283	0.98726772	0.54926624	0.30836565	6
2021-02-23-13-42-CSLIB	C	Dry	13	1.02246521	0.135328	0.109298	11.6113339	0.42753655	0.97768574	0.43729446	0.22869806	4
2021-02-25-10-54-CSLIB	C	Dry	10	0.96779811	0.12792	0.1064	10.1873072	0.39587484	0.97818406	0.40470384	0.19623269	4
2021-02-25-18-42-CSLIB	C	Dry	18	1.00419819	0.612583	0.149616	9.99282801	0.54557968	0.98702329	0.55275259	0.35417667	1
2021-03-01-06-36-CSLIB	C	Dry	06	1.02434761	0.652738	0.157806	8.9662943	0.54874845	0.98618046	0.55643817	0.31157895	5
2021-03-01-10-12-CSLIB	C	Dry	10	1.00136381	0.456093	0.137929	9.40870187	0.47316074	0.98649969	0.47963597	0.38479532	6

2021-03-02-09-48-CSLIB	C	Dry	09	1.00726349	0.154456	0.108591	13.3887688	0.46677974	0.97940755	0.47659397	0.08558941	3
2021-03-02-10-00-CSLIB	C	Dry	10	1.02657448	0.229375	0.114959	14.7616756	0.46650319	0.97510888	0.47841139	0.1047707	2
2021-03-03-06-42-CSLIB	C	Dry	06	0.99625784	0.973044	0.215258	9.26558853	0.57391394	0.98813918	0.58080274	0.30118806	5
2021-03-03-07-00-CSLIB	C	Dry	07	1.01433565	0.37375	0.127043	12.1858734	0.51824129	0.98599661	0.52560149	0.24434595	5
2021-03-03-08-36-CSLIB	C	Dry	08	1.04458472	0.661578	0.17316	12.5378222	0.54292555	0.98060826	0.55366202	0.08834718	4
2021-03-03-13-36-CSLIB	C	Dry	13	1.05531991	0.368938	0.135784	5.85871117	0.44146202	0.91930561	0.48021247	0.01030471	3
2021-03-03-15-48-CSLIB	C	Dry	15	1.03113307	0.225996	0.112506	13.047034	0.42000087	0.97491232	0.43080887	0.13961219	5
2021-03-03-17-30-CSLIB	C	Dry	17	1.0356964	0.401745	0.125977	11.8508509	0.48918138	0.95584437	0.51177932	0.04097261	1
2021-03-05-18-48-CSLIB	C	Dry	18	1.02101206	0.035799	0.101184	8.59447953	0.44763876	0.9844082	0.4547288	0.35597415	1
2021-03-06-09-06-CSLIB	C	Dry	09	1.00050604	0.561346	0.145483	12.1526559	0.50316903	0.98744073	0.50956884	0.22406894	6
2021-03-07-14-36-CSLIB	C	Dry	14	1.08545035	0.008684	0.100223	8.03820876	0.37112534	0.95377856	0.38911059	0.02573099	2
2021-03-07-16-48-CSLIB	C	Dry	16	1.07872635	0.033799	0.101459	9.99396006	0.39906103	0.97703238	0.40844197	0.1384426	6
2021-03-11-12-00-CSLIB	C	Dry	12	1.03252066	0.437604	0.139707	9.10146187	0.45522116	0.97441578	0.46717343	0.10095414	6
2021-03-11-13-24-CSLIB	C	Dry	13	1.0640518	0.046035	0.101916	7.87102598	0.36660542	0.93405066	0.39248987	0.01300092	2
2021-03-12-17-00-CSLIB	C	Dry	17	1.00801841	0.27842	0.121363	10.8771317	0.43436265	0.9804325	0.44303167	0.19787011	4
2021-03-14-13-00-CSLIB	C	Dry	13	0.99168288	0.300165	0.124611	11.4435432	0.42012598	0.98075308	0.4283708	0.10440135	5
2021-03-14-14-06-CSLIB	C	Dry	14	1.04384888	0.035844	0.101739	11.0627687	0.41314296	0.96899933	0.42636042	0.03257618	3
2021-03-16-14-24-CSLIB	C	Dry	14	1.00741581	0.29241	0.116725	10.2213924	0.44010443	0.98508748	0.44676685	0.43769775	6
2021-03-18-12-24-CSLIB	C	Dry	12	1.02691195	0.124197	0.107768	11.8724391	0.3758466	0.98280772	0.3824213	0.34073253	4
2021-03-20-06-54-CSLIB	C	Dry	06	1.00444123	0.40858	0.129518	10.8162887	0.46648986	0.98705349	0.47260849	0.05518006	6
2021-03-20-13-30-CSLIB	C	Dry	13	0.96955929	0.138977	0.1106	10.2129503	0.36579488	0.98406255	0.37171913	0.1820868	7
2021-03-22-08-42-CSLIB	C	Dry	08	0.99431327	0.342074	0.130206	7.5384119	0.4677506	0.98698394	0.47391916	0.06994152	6
2021-03-22-11-00-CSLIB	C	Dry	11	0.99555025	0.305117	0.122658	15.3521398	0.45368514	0.97814262	0.4638231	0.22081871	3
2021-03-22-14-24-CSLIB	C	Dry	14	1.04368917	0.375543	0.131427	5.98568609	0.38798328	0.90126496	0.43048748	0.00309018	4
2021-03-22-15-36-CSLIB	C	Dry	15	1.01551293	0.014332	0.100441	10.881507	0.3854019	0.95015391	0.4056205	0.02116344	3
2021-03-23-06-00-CSLIB	C	Dry	06	0.98702047	0.67089	0.162723	12.2176989	0.53922086	0.98771063	0.54593	0.31674977	5
2021-03-24-05-00-CSLIB	C	Dry	05	1.03003793	0.897965	0.212958	14.122856	0.54310746	0.98302233	0.55248741	0.29197907	5
2021-03-25-07-30-CSLIB	C	Dry	07	1.01054796	0.283407	0.121101	11.6171855	0.46000026	0.98451494	0.46723543	0.0767867	5

2021-03-26-07-36-CSLIB	C	Dry	07	1.00142138	0.440762	0.134189	14.726391	0.46700055	0.98724397	0.47303459	0.05894737	3
2021-03-27-06-36-CSLIB	C	Dry	06	1.0204274	0.359019	0.123371	10.330934	0.43836475	0.98768952	0.44382849	0.07859649	3
2021-03-27-17-12-CSLIB	C	Dry	17	1.04202559	0.094494	0.105997	8.33288202	0.45544363	0.97656071	0.46637513	0.1189289	2
2021-03-28-17-30-CSLIB	C	Dry	17	1.03779033	0.336698	0.123934	10.0225666	0.46374742	0.98313402	0.47170316	0.18629732	1
2021-03-28-18-12-CSLIB	C	Dry	18	1.02377909	0.685746	0.167421	10.9751594	0.51230662	0.95872591	0.53436192	0.01654663	0
2021-03-30-16-18-CSLIB	C	Dry	16	1.02718622	0.052618	0.102602	13.345608	0.4222946	0.97989625	0.43095849	0.14497999	2
2021-03-31-05-00-CSLIB	C	Dry	05	1.01999111	0.526052	0.147624	10.8487296	0.49034143	0.96956856	0.50573157	0.04034472	4
2021-03-31-16-48-CSLIB	C	Dry	16	1.08205112	0.081516	0.103175	9.92846015	0.36266775	0.93711666	0.38700384	0.0145891	3
2021-04-01-15-00-CSLIB	C	Dry	15	1.11668011	0.0293	0.100972	8.00557137	0.31104258	0.91722618	0.33911219	0.00435826	2
2021-04-01-16-54-CSLIB	C	Dry	16	1.05569747	0.550218	0.144965	11.8420794	0.56136075	0.98029258	0.57264613	0.03545706	0
2021-04-02-14-06-CSLIB	C	Dry	14	1.08252414	0.008049	0.100282	10.1459548	0.33730577	0.94825852	0.35571077	0.00384118	4
2021-04-04-09-12-CSLIB	C	Dry	09	1.00648005	0.255398	0.11426	11.333274	0.41217898	0.98537668	0.41829586	0.01024315	4
2021-04-04-15-00-CSLIB	C	Dry	15	1.03284215	0.332684	0.123786	11.0174461	0.44808878	0.97153718	0.46121629	0.00920899	5
2021-04-04-15-06-CSLIB	C	Dry	15	1.03301343	0.86197	0.207663	14.086776	0.50827191	0.98123934	0.51798974	0.06371191	4
2021-04-06-09-24-CSLIB	C	Dry	09	0.92606721	0.467437	0.156648	10.4484237	0.41111407	0.98016344	0.4194342	0.10762696	3
2021-04-06-16-18-CSLIB	C	Dry	16	1.04238096	0.433225	0.135219	9.44498255	0.47472408	0.98105008	0.48389383	0.20178517	1
2021-04-07-08-24-CSLIB	C	Dry	08	1.01767933	0.215737	0.112021	10.1599523	0.39621091	0.97317679	0.40713148	0.08244999	3
2021-04-08-05-24-CSLIB	C	Dry	05	1.01577778	0.291427	0.11774	12.0855228	0.40292055	0.98590215	0.40868209	0.17525392	4
2021-04-10-09-30-CSLIB	C	Dry	09	1.04171433	0.364085	0.128366	14.042547	0.42082217	0.96250545	0.43721537	0.03955679	7
2021-04-11-10-06-CSLIB	C	Dry	10	1.01558938	0.259473	0.117461	13.974783	0.43392291	0.954503	0.45460613	0.00027085	6
2021-04-13-08-00-CSLIB	C	Dry	08	0.96225462	1.003617	0.237572	9.90045783	0.59232265	0.98571382	0.60090732	0.12705448	3
2021-04-13-14-18-CSLIB	C	Dry	14	1.0308821	0.389975	0.126291	14.1191967	0.40118777	0.98197457	0.40855209	0.17373961	4
2021-04-14-11-18-CSLIB	C	Dry	11	0.95331735	0.959415	0.220259	8.46435869	0.53570385	0.98051396	0.54635005	0.09418283	2
2021-04-15-13-00-CSLIB	C	Dry	13	1.01335893	0.155116	0.107824	9.52179634	0.42322945	0.983833	0.43018424	0.21430594	4
2021-04-16-15-36-CSLIB	C	Dry	15	1.05258752	0.328801	0.133798	8.30173069	0.4395831	0.98102705	0.44808458	0.20800246	5
2021-04-19-13-12-CSLIB	C	Dry	13	1.01145956	0.291335	0.119186	7.19736374	0.47963739	0.98733437	0.48579023	0.10006771	2
2021-04-20-06-30-CSLIB	C	Dry	06	0.98291506	1.419208	0.3286	11.1565415	0.57850623	0.98626136	0.58656484	0.41237304	2
2021-04-21-14-36-CSLIB	C	Dry	14	0.99456994	0.117039	0.105094	8.17401148	0.47926245	0.98674925	0.48569832	0.16318867	3

2021-04-22-07-42-CSLIB	C	Dry	07	1.00414654	0.991837	0.275726	6.92307522	0.50766617	0.98624704	0.51474545	0.11609726	5
2021-04-22-09-00-CSLIB	C	Dry	09	0.9449784	1.520469	0.361147	11.0252453	0.61730753	0.98263908	0.6282139	0.25174515	5
2021-04-22-17-18-CSLIB	C	Dry	17	1.05461578	1.298929	0.303291	13.3009188	0.619674	0.98134873	0.63145137	0.08354571	1
2021-04-24-10-18-CSLIB	C	Dry	10	0.98150598	0.335664	0.133251	5.45100787	0.49827584	0.98764834	0.50450735	0.10113881	6
2021-04-24-16-12-CSLIB	C	Dry	16	1.0816537	1.160093	0.264559	13.8911666	0.57564068	0.98681834	0.58332994	0.08411203	1
2021-04-25-13-36-CSLIB	C	Dry	13	0.97569632	0.21846	0.112214	7.95443158	0.50023321	0.96130381	0.52036953	0.00066482	4
2021-04-26-06-30-CSLIB	C	Dry	06	0.96841994	1.078014	0.231693	7.5316155	0.70742167	0.97306991	0.72699984	0.09795014	2
2021-04-26-10-48-CSLIB	C	Dry	10	0.99106972	0.174461	0.111325	13.5163466	0.40235081	0.98781572	0.40731364	0.2550077	4
2021-04-26-17-18-CSLIB	C	Dry	17	1.07670751	0.608694	0.151285	13.6820768	0.58016257	0.97939543	0.59236805	0.11353647	0
2021-04-29-08-00-CSLIB	C	Dry	08	0.96954684	0.484106	0.150928	10.1674828	0.45067074	0.98657586	0.45680292	0.1859526	5
2021-05-01-12-48-CSLIB	C	Wet	12	0.93097757	1.532348	0.361769	8.10386376	0.69611199	0.98178896	0.70902405	0.2498615	4
2021-05-01-16-06-CSLIB	C	Wet	16	0.98022094	0.409085	0.135562	7.03150561	0.49677405	0.98795557	0.50283035	0.17141274	2
2021-05-02-11-12-CSLIB	C	Wet	11	0.93910822	0.923014	0.251182	9.8308918	0.5820651	0.98796738	0.58915417	0.02698677	3
2021-05-02-11-18-CSLIB	C	Wet	11	0.97198045	1.176614	0.301399	6.96593065	0.62874792	0.98612738	0.63759301	0.02515236	5
2021-05-04-08-06-CSLIB	C	Wet	08	0.944122	0.214682	0.11468	8.58397151	0.55788828	0.98094664	0.56872439	0.23933518	3
2021-05-06-08-00-CSLIB	C	Wet	08	0.97034235	0.646995	0.169264	9.55146705	0.60675478	0.97030319	0.62532494	0.05633734	5
2021-05-08-09-06-CSLIB	C	Wet	09	0.96807694	1.623272	0.446252	10.9876076	0.70636937	0.98550047	0.71676209	0.46499231	5
2021-05-09-10-24-CSLIB	C	Wet	10	0.97823591	1.629416	0.440341	13.8410433	0.74582413	0.98535541	0.75690875	0.22936288	1
2021-05-11-08-24-CSLIB	C	Wet	08	1.02570113	1.890847	0.520123	16.1103181	0.79300702	0.97383249	0.81431563	0.13156048	2
2021-05-11-09-30-CSLIB	C	Wet	09	0.98857364	1.832535	0.485834	14.0515809	0.74076716	0.9837588	0.75299673	0.25440443	3
2021-05-11-10-42-CSLIB	C	Wet	10	0.97401432	1.661237	0.445195	10.5038339	0.76679938	0.9851317	0.77837245	0.54751616	4
2021-05-12-09-54-CSLIB	C	Wet	09	0.98223533	1.640374	0.430122	12.9725574	0.74912616	0.98345004	0.76173281	0.48256079	3
2021-05-15-13-00-CSLIB	C	Wet	13	0.96930611	0.8203	0.185219	10.2321037	0.61058874	0.97572384	0.62578028	0.08376731	3
2021-05-16-13-18-CSLIB	C	Wet	13	0.97763033	0.016836	0.10057	6.95021817	0.68631403	0.96151605	0.71378323	0.01730994	3
2021-05-17-16-54-CSLIB	C	Wet	16	1.10484128	0.06205	0.102369	16.8813882	0.47919613	0.95666475	0.50090288	0.03068021	1
2021-05-19-15-48-CSLIB	C	Wet	15	0.98289838	0.17914	0.109753	7.42997058	0.54709588	0.97123966	0.56329648	0.05606648	2
2021-05-21-07-18-CSLIB	C	Wet	07	0.99315888	1.803682	0.511422	12.2873113	0.80032794	0.98746143	0.81049033	0.70371191	3
2021-05-21-15-42-CSLIB	C	Wet	15	1.03602419	0.434234	0.141985	13.0662412	0.48199128	0.98738576	0.4881489	0.12070175	2

2021-05-22-09-12-CSLIB	C	Wet	09	0.9902269	1.676198	0.44757	12.4043803	0.76315004	0.98044098	0.77837428	0.30040012	3
2021-05-22-14-54-CSLIB	C	Wet	14	0.99548902	1.88966	0.518493	7.63534781	0.77505082	0.98422503	0.78747318	0.1358695	3
2021-05-22-15-42-CSLIB	C	Wet	15	1.01107897	0.259816	0.121461	9.54988544	0.43620195	0.98814248	0.44143629	0.07213296	6
2021-05-23-05-06-CSLIB	C	Wet	05	0.99638494	1.655588	0.439149	13.8818932	0.81010481	0.98712911	0.82066753	0.84052939	3
2021-05-23-05-30-CSLIB	C	Wet	05	0.99834831	1.618609	0.416954	14.8927048	0.80451978	0.98441147	0.81725965	0.60584795	4
2021-05-25-11-06-CSLIB	C	Wet	11	0.98489067	1.695413	0.450983	10.4734846	0.77391691	0.98606496	0.78485388	0.5736165	4
2021-05-28-12-54-CSLIB	C	Wet	12	0.97350522	1.410809	0.324775	8.27400344	0.67473565	0.97605058	0.69129169	0.19304401	2
2021-05-29-17-00-CSLIB	C	Wet	17	0.99167796	1.151552	0.253154	13.4085395	0.61556704	0.98936747	0.62218241	0.05614035	1
2021-05-30-06-24-CSLIB	C	Wet	06	0.99511242	1.68187	0.448258	14.5999601	0.79874135	0.98624519	0.80988111	0.65408433	4
2021-06-01-09-00-CSLIB	C	Wet	09	0.98514468	1.670754	0.448757	12.5703141	0.76761446	0.98454578	0.77966356	0.5497076	3
2021-06-02-11-48-CSLIB	C	Wet	11	0.99015614	0.298314	0.128895	5.09621935	0.52769888	0.97895739	0.53904173	0.26874731	1
2021-06-02-16-18-CSLIB	C	Wet	16	1.02959158	0.4458	0.130465	13.3982646	0.54060965	0.98180607	0.55062773	0.35265005	3
2021-06-04-05-06-CSLIB	C	Wet	05	1.00903131	1.896573	0.578525	14.928716	0.81659829	0.98771027	0.82675893	0.92369344	0
2021-06-04-09-06-CSLIB	C	Wet	09	0.99289058	1.769668	0.492227	11.4242113	0.79221772	0.98712747	0.80254856	0.60634041	4
2021-06-05-05-42-CSLIB	C	Wet	05	0.99835458	1.807259	0.500986	11.4327091	0.79270892	0.98639529	0.80364224	0.65684211	5
2021-06-06-05-06-CSLIB	C	Wet	05	1.01158997	1.846558	0.536267	14.4561058	0.80872645	0.98484963	0.82116744	0.67767313	3
2021-06-06-05-48-CSLIB	C	Wet	05	1.02074545	1.798601	0.497147	11.3097837	0.80916912	0.98571628	0.82089455	0.39091413	3
2021-06-06-11-24-CSLIB	C	Wet	11	0.99094297	1.626546	0.418963	11.4154085	0.75798336	0.98531481	0.76928039	0.13188058	3
2021-06-06-12-00-CSLIB	C	Wet	12	0.98274369	1.683124	0.450148	11.4476804	0.74433304	0.98386057	0.75654322	0.21052632	4
2021-06-08-18-12-CSLIB	C	Wet	18	1.01732317	0.817769	0.196357	12.5576784	0.55605942	0.9828278	0.56577502	0.27516159	0
2021-06-09-16-24-CSLIB	C	Wet	16	1.00311028	0.097369	0.104978	7.23127357	0.57947208	0.98126017	0.59053868	0.17280394	1
2021-06-12-07-36-CSLIB	C	Wet	07	1.00171597	1.715808	0.454365	10.7783798	0.75918619	0.985458	0.77038919	0.57396122	1
2021-06-16-06-24-CSLIB	C	Wet	06	0.99302653	1.636589	0.424242	11.8366792	0.76745369	0.98263567	0.7810155	0.3080948	3
2021-06-16-12-24-CSLIB	C	Wet	12	0.96382367	1.700012	0.462727	7.43371903	0.75858513	0.98784748	0.76791726	0.22603878	2
2021-06-18-08-30-CSLIB	C	Wet	08	0.94423245	2.206799	0.762316	4.18919353	0.81544079	0.98739235	0.82585285	0.85775316	2
2021-06-18-11-24-CSLIB	C	Wet	11	0.96596091	1.511951	0.363471	7.37436136	0.70488714	0.98512447	0.71553104	0.2374269	4
2021-06-20-07-48-CSLIB	C	Wet	07	0.95755153	1.626103	0.392749	14.1062601	0.71430518	0.98183971	0.72751711	0.07694675	3
2021-06-23-12-06-CSLIB	C	Wet	12	0.97327353	1.574179	0.367711	9.28158656	0.74569639	0.98735892	0.75524349	0.35929825	2

2021-06-23-15-00-CSLIB	C	Wet	15	1.007083	0.884029	0.226156	7.63064406	0.56636914	0.98799984	0.57324821	0.08802709	3
2021-06-23-16-36-CSLIB	C	Wet	16	0.98184917	1.992616	0.591685	15.631296	0.78468424	0.98888907	0.79350077	0.52959064	0
2021-06-25-14-42-CSLIB	C	Wet	14	0.9930656	0.444564	0.14753	6.03363132	0.52988453	0.98634391	0.53722087	0.34136042	3
2021-06-25-18-00-CSLIB	C	Wet	18	1.05223	1.037047	0.229155	12.983196	0.58123703	0.9881001	0.58823699	0.43310557	1
2021-06-25-18-24-CSLIB	C	Wet	18	1.04577719	1.039679	0.227449	15.2341273	0.57235513	0.98734475	0.57969127	0.42382272	2
2021-06-30-07-48-CSLIB	C	Wet	07	0.95386269	0.853429	0.197411	7.91419512	0.55511853	0.98052666	0.56614323	0.21303786	2
2021-07-01-08-48-CSLIB	C	Wet	08	0.9300809	0.249568	0.114668	8.74445435	0.47055224	0.97582913	0.48220762	0.13407202	3
2021-07-04-10-12-CSLIB	C	Wet	10	0.96324206	1.668961	0.449637	11.2274707	0.71129063	0.97849836	0.72692062	0.08947984	3
2021-07-04-14-06-CSLIB	C	Wet	14	1.03314304	0.371531	0.140619	7.2388229	0.50194609	0.97879816	0.5128188	0.24631579	1
2021-07-06-07-42-CSLIB	C	Wet	07	0.94526139	1.630934	0.405326	9.92980324	0.66602036	0.98564106	0.67572303	0.58478301	3
2021-07-06-15-36-CSLIB	C	Wet	15	1.00479875	0.548166	0.160133	10.3074093	0.48873586	0.98827673	0.4945334	0.67565405	2
2021-07-07-10-00-CSLIB	C	Wet	10	0.96137648	0.129696	0.111286	7.72064546	0.46438172	0.98251633	0.47264529	0.41366574	4
2021-07-09-05-48-CSLIB	C	Wet	05	0.94905833	1.431116	0.322723	9.87710888	0.68023931	0.97518239	0.69755086	0.11862111	5
2021-07-09-08-00-CSLIB	C	Wet	08	0.9685563	0.648061	0.156804	10.9063471	0.59576152	0.97457354	0.61130484	0.13185596	2
2021-07-10-11-36-CSLIB	C	Wet	11	0.9612522	0.350999	0.137459	8.47086065	0.50473337	0.97552574	0.51739626	0.14158203	5
2021-07-11-11-12-CSLIB	C	Wet	11	0.94442925	0.370649	0.129569	9.7373548	0.61638991	0.96771155	0.63695624	0.0543675	4
2021-07-12-08-54-CSLIB	C	Wet	08	0.94450218	0.07146	0.103666	10.1634251	0.40770915	0.98278679	0.41485005	0.36919668	2
2021-07-13-06-48-CSLIB	C	Wet	06	0.96597423	1.579929	0.381522	9.11071084	0.67966575	0.98677946	0.68877169	0.36498615	3
2021-07-13-16-42-CSLIB	C	Wet	16	1.06419512	0.638953	0.152479	12.4344232	0.48247434	0.98591404	0.48936755	0.46044937	1
2021-07-14-14-12-CSLIB	C	Wet	14	0.96838305	0.216616	0.112161	9.77915373	0.39453131	0.98668504	0.39985537	0.38546014	4
2021-07-15-05-06-CSLIB	C	Wet	05	0.95394789	0.042877	0.10248	6.31301265	0.5327827	0.97704288	0.54530125	0.13569714	5
2021-07-15-05-12-CSLIB	C	Wet	05	0.96721599	1.431652	0.326917	11.4312836	0.6424683	0.98658606	0.6512035	0.69444137	5
2021-07-15-06-36-CSLIB	C	Wet	06	0.98364968	2.034525	0.627643	12.3009334	0.79068817	0.98591809	0.8019816	0.72102185	2
2021-07-15-10-00-CSLIB	C	Wet	10	0.99328368	1.488666	0.345952	6.7326293	0.8302985	0.98461861	0.84326915	0.58149585	0
2021-07-15-12-48-CSLIB	C	Wet	12	0.92693568	0.788636	0.18546	9.74318368	0.58743845	0.98111787	0.59874402	0.31580179	2
2021-07-18-13-30-CSLIB	C	Wet	13	1.00365278	0.504248	0.153994	9.35729547	0.50835004	0.98139767	0.51798578	0.05811019	4
2021-07-19-08-00-CSLIB	C	Wet	08	0.97983927	0.132349	0.109348	5.66534753	0.46522312	0.986778	0.47145672	0.50351493	5
2021-07-20-18-12-CSLIB	C	Wet	18	0.98654061	0.271694	0.115703	10.787929	0.41253245	0.98105178	0.42050018	0.11069252	1

2021-07-21-13-06-CSLIB	C	Wet	13	0.98876106	0.466854	0.140594	7.306318	0.60861497	0.98333052	0.61893225	0.38017852	2
2021-07-22-05-36-CSLIB	C	Wet	05	1.00181612	0.257124	0.121114	16.0904811	0.45089525	0.98672022	0.45696363	0.31911357	6
2021-07-22-08-24-CSLIB	C	Wet	08	0.99083149	1.311721	0.281406	7.29203921	0.6836393	0.98693025	0.69269262	0.64509695	3
2021-07-22-10-12-CSLIB	C	Wet	10	0.99078129	0.330994	0.135301	9.9704043	0.48967622	0.98595553	0.49665143	0.3143121	4
2021-07-22-18-48-CSLIB	C	Wet	18	1.00582261	0.83663	0.180847	7.40876822	0.70256414	0.98367949	0.71422059	0.08635272	0
2021-07-23-18-48-CSLIB	C	Wet	18	1.00615128	0.104243	0.104378	6.6699452	0.59092136	0.9626044	0.61387768	0.01923053	0
2021-07-25-07-00-CSLIB	C	Wet	07	0.98201177	1.592724	0.376384	5.02464004	0.73584775	0.98736304	0.74526564	0.24679594	1
2021-07-26-18-48-CSLIB	C	Wet	18	1.00298507	0.305978	0.122006	6.85312976	0.47550659	0.98801504	0.48127465	0.02038781	0
2021-07-27-18-00-CSLIB	C	Wet	18	1.02541168	0.048078	0.103233	6.41346295	0.44339483	0.98317551	0.45098238	0.01370268	0
2021-07-29-10-54-CSLIB	C	Wet	10	0.99413582	1.908737	0.537217	16.198411	0.78511138	0.9890703	0.79378724	0.99649123	1
2021-07-29-12-00-CSLIB	C	Wet	12	1.04462692	0.059991	0.103315	13.4992466	0.43073868	0.98344635	0.437989	0.40536781	1
2021-07-30-12-36-CSLIB	C	Wet	12	0.98080544	1.312493	0.298797	8.89662302	0.71338597	0.98791655	0.72211157	0.52240074	0
2021-08-01-06-48-CSLIB	C	Wet	06	1.00545218	0.428548	0.137406	16.2896416	0.47920042	0.98763529	0.48519977	0.11700831	4
2021-08-03-15-24-CSLIB	C	Wet	15	1.02652106	1.488979	0.354671	7.29851812	0.63981729	0.98831398	0.64738261	0.22914127	0
2021-08-04-11-30-CSLIB	C	Wet	11	0.99433353	1.369731	0.312964	7.78025748	0.65711951	0.98315405	0.66837899	0.39487842	1
2021-08-05-09-42-CSLIB	C	Wet	09	0.98781251	0.57242	0.156008	6.20422218	0.55390625	0.98507129	0.56230068	0.35634349	4
2021-08-06-12-48-CSLIB	C	Wet	12	0.9969517	0.027345	0.100888	8.27627119	0.62588376	0.95960372	0.65223149	0.01527855	1
2021-08-07-06-06-CSLIB	C	Wet	06	0.99688459	0.941872	0.21647	6.71119257	0.58887008	0.9857094	0.59740739	0.13963681	5
2021-08-09-14-54-CSLIB	C	Wet	14	0.9602338	0.060035	0.102176	4.22098395	0.35456101	0.98442876	0.36016929	0.06640813	1
2021-08-13-11-00-CSLIB	C	Wet	11	0.98914491	1.249918	0.274195	17.5098341	0.74078997	0.98314999	0.75348622	0.54701139	0
2021-08-15-15-36-CSLIB	C	Wet	15	1.02248473	0.117122	0.105728	6.41739884	0.41219051	0.9828702	0.41937431	0.10341644	0
2021-08-16-08-54-CSLIB	C	Wet	08	1.00645052	0.026003	0.10126	6.45932252	0.36031559	0.98022889	0.36758312	0.05146199	3
2021-08-22-11-54-CSLIB	C	Wet	11	0.99181751	1.065876	0.24352	11.5114637	0.67394727	0.97712133	0.68972732	0.04866728	2
2021-08-26-17-00-CSLIB	C	Wet	17	0.98838912	0.042196	0.101772	13.7089977	0.42550906	0.98418811	0.43234526	0.14517698	0
2021-08-27-10-00-CSLIB	C	Wet	10	0.98793547	0.30539	0.120332	8.35303943	0.51103649	0.98555826	0.51852489	0.49068637	3
2021-08-27-13-30-CSLIB	C	Wet	13	0.9841629	1.40852	0.322222	9.38571609	0.59182395	0.98520277	0.60071284	0.43915051	2
2021-08-29-06-36-CSLIB	C	Wet	06	0.99940779	0.06356	0.104517	11.7999316	0.35043767	0.9797122	0.35769451	0.09423207	3
2021-08-29-17-18-CSLIB	C	Wet	17	1.07513652	0.88346	0.198695	11.7057486	0.49012637	0.97746594	0.50142552	0.02888273	0

2021-08-30-11-42-CSLIB	C	Wet	11	1.05240003	0.097944	0.107574	10.7327699	0.42631323	0.93714905	0.45490441	0.02016621	1
2021-08-31-09-00-CSLIB	C	Wet	09	0.96904178	0.757979	0.168097	9.22339094	0.52386562	0.976155	0.53666233	0.18736842	2
2021-09-01-09-00-CSLIB	C	Wet	09	0.97594339	1.047943	0.227636	18.2197204	0.71600957	0.97539455	0.73407174	0.17132656	0
2021-09-02-10-54-CSLIB	C	Wet	10	0.98985283	1.286246	0.279257	13.6169036	0.77433912	0.98444164	0.78657697	0.60088643	1
2021-09-03-17-48-CSLIB	C	Wet	17	0.99482908	0.322072	0.129556	9.35231834	0.52140955	0.96393654	0.54091689	0.05493383	0
2021-09-05-09-00-CSLIB	C	Wet	09	0.9599189	0.084309	0.106769	6.25558627	0.37914744	0.9808017	0.38656891	0.00658664	2
2021-09-05-17-30-CSLIB	C	Wet	17	1.00984121	0.030643	0.10106	5.99853312	0.3694777	0.97485886	0.37900635	0.03811634	0
2021-09-06-12-54-CSLIB	C	Wet	12	1.00513804	0.159622	0.113373	9.62646867	0.39604446	0.98441108	0.40231613	0.59351185	3
2021-09-07-06-18-CSLIB	C	Wet	06	0.99179131	0.764538	0.171611	7.81279413	0.64553441	0.984371	0.65578365	0.57585719	0
2021-09-12-07-30-CSLIB	C	Wet	07	0.99913215	1.996638	0.595426	12.7625229	0.77543991	0.97552662	0.79489364	0.08853186	3
2021-09-12-18-00-CSLIB	C	Wet	18	1.03307966	0.195315	0.109716	17.4715523	0.43439438	0.96996582	0.44784504	0.04062789	0
2021-09-16-16-54-CSLIB	C	Wet	16	0.9894509	0.064148	0.103334	6.97348799	0.49028372	0.97875734	0.50092469	0.12705448	0
2021-09-18-18-12-CSLIB	C	Wet	18	1.00426166	0.30369	0.120136	12.1644914	0.38305091	0.97642532	0.39229924	0.1528101	0
2021-09-19-10-24-CSLIB	C	Wet	10	0.98225599	1.562522	0.367705	10.8650666	0.63657069	0.9866057	0.64521287	0.4072884	2
2021-09-19-18-30-CSLIB	C	Wet	18	0.98289562	0.160378	0.110122	10.8766276	0.41234769	0.9798029	0.42084759	0.03921207	0
2021-09-20-18-12-CSLIB	C	Wet	18	0.99268962	0.409312	0.128723	7.59489183	0.46730726	0.98176454	0.4759871	0.28838412	0
2021-09-21-11-18-CSLIB	C	Wet	11	0.98132549	0.319073	0.122829	15.5914321	0.56746233	0.95511272	0.59413127	0.04062789	0
2021-09-23-06-18-CSLIB	C	Wet	06	0.98724942	0.138619	0.110819	8.64001305	0.38998859	0.97537517	0.39983445	0.12705448	7
2021-09-23-17-06-CSLIB	C	Wet	17	1.03355838	0.20978	0.112138	8.90330408	0.41729732	0.98217939	0.42486874	0.19693444	1
2021-09-25-13-48-CSLIB	C	Wet	13	0.98366915	1.738542	0.438971	11.0196346	0.7652763	0.98787466	0.77466943	0.8941582	1
2021-09-25-15-00-CSLIB	C	Wet	15	0.9826498	0.705595	0.170306	16.1661012	0.508932	0.98462698	0.51687798	0.28038166	0
2021-09-26-13-48-CSLIB	C	Wet	13	1.04457384	0.642218	0.157054	10.997774	0.45963062	0.98357546	0.4673059	0.11036011	0
2021-10-01-06-54-CSLIB	C	Wet	06	0.93970851	0.150742	0.113445	13.0127858	0.4183759	0.98040277	0.42673879	0.16002462	3
2021-10-04-08-42-CSLIB	C	Wet	08	0.99826826	0.021632	0.100873	8.61260106	0.5460699	0.96218038	0.56753381	0.06672822	0
2021-10-08-16-00-CSLIB	C	Wet	16	0.98603563	0.262116	0.11734	9.98376711	0.40964563	0.97773355	0.41897471	0.08178517	1
2021-10-09-17-12-CSLIB	C	Wet	17	1.01404602	0.035888	0.101373	6.87583711	0.38229637	0.98043396	0.38992567	0.03047091	1
2021-10-15-07-12-CSLIB	C	Wet	07	0.96906477	0.688205	0.187829	9.18258182	0.47330402	0.9769178	0.48448704	0.19146814	4
2021-10-17-12-18-CSLIB	C	Wet	12	0.98971286	0.109164	0.106273	10.7161476	0.40643956	0.9850054	0.41262673	0.15020006	0

2021-10-19-18-00-CSLIB	C	Wet	18	0.9593591	0.012701	0.100516	15.3277281	0.36910515	0.96936857	0.38076863	0.05032933	1
2021-10-21-17-54-CSLIB	C	Wet	17	1.01577158	0.119353	0.105963	13.2463943	0.35522691	0.97682359	0.36365513	0.10548477	0
2021-11-17-16-00-CSLIB	C	Dry	16	1.01226117	0.046006	0.102329	8.45858551	0.36054772	0.98005347	0.36788577	0.19578947	0
2021-11-18-12-06-CSLIB	C	Dry	12	0.98769271	0.097449	0.105145	11.7039247	0.41879513	0.98164602	0.4266254	0.35901508	0
2021-11-22-11-42-CSLIB	C	Dry	11	0.9459092	0.007376	0.100185	5.73760649	0.35270433	0.9742591	0.36202313	0.08470299	0
2021-11-24-07-24-CSLIB	C	Dry	07	0.97194426	0.010522	0.100447	8.84863065	0.40455171	0.9701986	0.41697825	0.09465066	2
2021-11-29-13-54-CSLIB	C	Dry	13	1.04972153	0.001466	0.10003	4.30222922	0.34196273	0.96899872	0.35290319	0.042253	1
2021-12-03-12-18-CSLIB	C	Dry	12	0.9885849	0.128093	0.113799	3.70824687	0.38874354	0.98470234	0.39478279	0.39091413	0
2021-12-07-07-48-CSLIB	C	Dry	07	0.96879798	0.181639	0.112183	15.8004902	0.39574246	0.98394197	0.40220101	0.47645429	3
2021-12-10-07-42-CSLIB	C	Dry	07	1.01024395	0.312619	0.129518	11.3912597	0.49491994	0.97563024	0.50728229	0.16004925	3
2021-12-18-05-30-CSLIB	C	Dry	05	1.05846548	0.238872	0.117968	15.5985989	0.43768469	0.97785059	0.44759874	0.23596183	5
2021-12-31-16-00-CSLIB	C	Dry	16	1.0898058	0.134005	0.107815	10.6796572	0.48614745	0.98237355	0.49487026	0.39468144	0
2022-01-05-09-00-CSLIB	C	Dry	09	1.02266949	0.307683	0.118404	6.46606442	0.46485619	0.978903	0.47487462	0.25137581	1
2022-01-06-07-48-CSLIB	C	Dry	07	0.9982299	0.160231	0.110728	10.3810162	0.40078281	0.98493336	0.40691363	0.34299785	3
2022-01-08-15-36-CSLIB	C	Dry	15	0.99840425	0.260972	0.114878	11.0399593	0.47990852	0.98419325	0.48761615	0.10241921	1
2022-01-13-17-18-CSLIB	C	Dry	17	0.98247845	0.001024	0.100032	11.5350857	0.46433082	0.97761528	0.47496273	0.01485996	0
2022-01-14-16-00-CSLIB	C	Dry	16	1.00859967	0.072253	0.102729	9.03497033	0.42746439	0.97909832	0.43658985	0.16743613	0
2022-01-15-11-24-CSLIB	C	Dry	11	1.01101188	0.033748	0.1011	8.34317469	0.43283849	0.98334955	0.44016748	0.25767929	1
2022-01-17-18-12-CSLIB	C	Dry	18	1.02306795	0.009307	0.100241	10.6700422	0.43571033	0.97489372	0.4469311	0.02329332	0
2022-01-18-11-54-CSLIB	C	Dry	11	1.02693429	0.042772	0.101443	9.10478944	0.44130431	0.98047675	0.45009156	0.18403201	1
2022-01-18-17-42-CSLIB	C	Dry	17	0.98509226	0.029043	0.100908	9.88333965	0.39674153	0.97748593	0.40587953	0.15684826	1
2022-01-21-08-42-CSLIB	C	Dry	08	1.02254389	0.156339	0.108266	17.4952711	0.44087133	0.9843105	0.44789864	0.56377962	2
2022-01-24-07-30-CSLIB	C	Dry	07	1.00054926	0.189151	0.109231	7.77874388	0.39488757	0.98306861	0.40168872	0.19273623	3
2022-01-25-08-36-CSLIB	C	Dry	08	1.01973155	0.119689	0.10568	11.9542363	0.44407871	0.98587925	0.45043926	0.42674054	4
2022-01-25-14-00-CSLIB	C	Dry	14	0.99918061	0.061422	0.102235	11.7126676	0.44216474	0.97673358	0.45269739	0.04731302	0
2022-01-28-13-00-CSLIB	C	Dry	13	1.04050471	0.914759	0.200107	12.4191254	0.53994188	0.98573058	0.54775807	0.44348415	2
2022-01-28-16-00-CSLIB	C	Dry	16	1.05388671	0.299907	0.117411	10.4657934	0.4390775	0.97679645	0.44950767	0.16792859	0
2022-01-29-10-48-CSLIB	C	Dry	10	0.99321914	0.042916	0.101475	11.0509918	0.46050812	0.98424447	0.46787981	0.0440751	0

2022-01-30-17-54-CSLIB	C	Dry	17	1.02368573	0.188359	0.109295	10.2327775	0.37171367	0.97343405	0.38185809	0.02152047	0
2022-01-31-18-00-CSLIB	C	Dry	18	0.9935575	0.213275	0.110931	11.993555	0.43611442	0.97742585	0.44618671	0.00083718	0
2022-01-31-18-06-CSLIB	C	Dry	18	1.02762755	0.335394	0.120068	12.0288605	0.44283901	0.98222314	0.45085377	0.02039089	0

The filename of each *.wav* file based on the YYYY-MM-DD-HH-mm timestamp (WAV), Normalized Acoustic Complexity index (nACI), Acoustic Diversity index (ADI), Inverse Acoustic Evenness index (1-AEI), Bioacoustic index (BI), Acoustic Entropy index (H), Temporal Entropy Index (Ht), Spectral Entropy Index (Hf), Acoustic Richness Index (AR), and Number of species heard (SH).
