# Supplementary material for: Acoustic indices as proxies for bird species richness in an urban green space in Metro Manila
Source: PLoS One. 2023 Jul 28;18(7):e0289001. doi: 10.1371/journal.pone.0289001 (PMC10381043; doi:10.1371/journal.pone.0289001)
Supplement: S4 Table — (PDF) [file pone.0289001.s005.pdf]

S4 Table. The results of the acoustic index analysis for 840 pre-processed 5-min sound samples.

WAV	Site	Season	Hour	nACI	ADI	1-AEI	BI	H	Ht	Hf	AR	SH
2021-05-02-08-18-IB100p	A	Wet	08	1.02237803	2.266279	0.856061	3.48985376	0.87979972	0.98904646	0.88954337	0.6446281	3
2021-05-04-07-36-IB100p	A	Wet	07	1.10139084	2.168751	0.730718	8.90222451	0.8012504	0.99056515	0.80888208	0.97727273	3
2021-05-09-13-00-IB100p	A	Wet	13	1.01458471	2.29749	0.942925	3.68765324	0.89955445	0.98697276	0.91142783	0.12551653	2
2021-05-13-11-30-IB100p	A	Wet	11	1.04320343	2.260886	0.836628	7.27840848	0.81032135	0.95518755	0.84833742	0.01497934	2
2021-06-20-10-24-IB100p	A	Wet	10	1.00662254	2.289167	0.909525	7.01733302	0.84589823	0.98897171	0.85533107	0.57334711	4
2021-06-22-16-54-IB100p	A	Wet	16	1.01007156	2.302157	0.983334	2.74269967	0.89752134	0.98982044	0.90675167	0.35123967	5
2021-07-13-13-54-IB100p	A	Wet	13	1.07658431	2.188797	0.73421	2.98560456	0.84032701	0.98662824	0.85171596	0.43904959	4
2021-07-19-16-18-IB100p	A	Wet	16	1.00821768	2.299646	0.956684	3.5549032	0.86230335	0.98927091	0.87165542	0.45196281	5
2021-08-01-09-30-IB100p	A	Wet	09	1.0194158	1.915288	0.546272	2.14478842	0.86043949	0.96981645	0.8872189	0.04338843	5
2021-08-13-07-06-IB100p	A	Wet	07	0.99827472	2.298938	0.953201	4.04218123	0.83350241	0.99105039	0.8410293	0.61363636	5
2021-08-13-12-06-IB100p	A	Wet	12	1.02431246	2.298199	0.947295	2.82276166	0.88330991	0.98931116	0.89285347	0.14876033	3
2021-08-25-16-12-IB100p	A	Wet	16	1.08851407	2.301882	0.978973	2.31375178	0.90273253	0.98905049	0.91272644	0.32386364	2
2021-09-13-13-18-IB100p	A	Wet	13	1.01979476	1.682703	0.428761	4.48428757	0.86392603	0.96586428	0.89445903	0.01704546	3
2021-09-19-10-42-IB100p	A	Wet	10	1.01167801	2.188243	0.733311	2.97183094	0.90767257	0.98826048	0.91845479	0.19473141	4
2021-09-27-08-42-IB100p	A	Wet	08	1.07326613	2.267402	0.850465	1.81781316	0.90072555	0.97960815	0.91947535	0.15495868	5
2021-10-01-17-00-IB100p	A	Wet	17	1.02152724	2.167251	0.775296	11.7436169	0.84965113	0.99041403	0.85787469	0.93285124	1
2021-10-05-10-48-IB100p	A	Wet	10	1.06303157	2.301677	0.976964	2.92714859	0.87759293	0.98957623	0.88683712	0.68698347	3
2021-10-12-15-06-IB100p	A	Wet	15	1.07292474	2.292136	0.918421	1.54566819	0.88868953	0.9900878	0.89758659	0.59297521	4
2021-10-30-15-48-IB100p	A	Wet	15	1.0197854	2.166154	0.717471	3.49040172	0.88576426	0.98405735	0.90011447	0.04532541	1
2020-10-08-18-00-IB100p	A	Wet	18	1.07698141	1.430809	0.329339	8.4646084	0.74300358	0.92770902	0.80090154	0.00063776	0
2020-10-25-06-06-IB100p	A	Wet	06	1.20320986	2.239123	0.7996	7.12057002	0.87359285	0.98551936	0.8864289	0.33928571	3
2020-11-01-12-12-IB100p	A	Dry	12	1.15486846	2.275072	0.876875	8.83828056	0.90082238	0.9815911	0.91771654	0.34151786	2
2020-11-05-09-06-IB100p	A	Dry	09	1.028816	1.791079	0.4601	9.41261762	0.82039044	0.98493733	0.8329367	0.38966837	2
2020-11-07-09-30-IB100p	A	Dry	09	1.02324355	2.262559	0.843686	9.45972835	0.84028716	0.98752502	0.85090214	0.17346939	1
2020-11-25-17-30-IB100p	A	Dry	17	1.02086403	2.299603	0.956967	7.64908226	0.82063714	0.98996437	0.82895625	0.28125	1

2020-11-26-10-48-IB100p	A	Dry	10	1.02912646	1.162802	0.292701	6.97178675	0.73706834	0.96922237	0.76047392	0.12627551	2
2020-12-10-05-42-IB100p	A	Dry	05	1.15020245	1.712065	0.484552	8.6876979	0.81001924	0.97745617	0.82870134	0.29846939	2
2020-12-11-08-30-IB100p	A	Dry	08	1.03186261	1.227811	0.27449	5.38704755	0.75205693	0.98829302	0.76096553	0.56122449	7
2020-12-28-05-54-IB100p	A	Dry	05	1.05838919	0.853577	0.21268	4.92178368	0.73324349	0.9847006	0.74463598	0.29846939	2
2020-12-29-12-00-IB100p	A	Dry	12	1.01538838	2.277987	0.875479	3.67866383	0.84058454	0.99097399	0.84824077	0.06760204	2
2020-12-31-16-12-IB100p	A	Dry	16	1.02909226	2.002037	0.590152	2.76292909	0.85898891	0.96663066	0.88864231	0.04081633	2
2021-01-01-08-12-IB100p	A	Dry	08	1.01424551	1.299354	0.290169	5.71852182	0.71412722	0.99126469	0.72042032	0.64891582	5
2021-01-05-11-00-IB100p	A	Dry	11	1.01729963	2.298088	0.946298	2.45241774	0.86506818	0.99075428	0.873141	0.01658163	2
2021-01-12-09-12-IB100p	A	Dry	09	1.01286657	2.201514	0.752265	8.46245235	0.81019086	0.9463747	0.85609945	0.00765306	4
2021-01-14-08-36-IB100p	A	Dry	08	1.0730248	1.743623	0.439386	3.66140825	0.79370559	0.96860059	0.81943538	0.15943878	4
2021-01-16-07-54-IB100p	A	Dry	07	1.03670324	1.586931	0.381778	6.02744007	0.73560113	0.99004703	0.74299615	0.65369898	4
2021-01-18-14-42-IB100p	A	Dry	14	1.02424891	2.113743	0.659236	3.68127795	0.84866972	0.98351042	0.86289855	0.14030612	1
2021-01-19-05-42-IB100p	A	Dry	05	1.02895441	1.906446	0.556196	9.21209745	0.70967972	0.9903522	0.71659327	0.78061225	0
2021-01-27-14-54-IB100p	A	Dry	14	1.01644569	2.228132	0.795812	9.35438957	0.87554437	0.98648092	0.88754313	0.22193878	1
2021-02-04-06-06-IB100p	A	Dry	06	1.03160902	1.529512	0.362535	8.19178742	0.7254876	0.9891321	0.73345875	0.5625	3
2021-02-05-11-00-IB100p	A	Dry	11	1.04619441	1.801561	0.518055	7.56579998	0.79714937	0.98393252	0.8101667	0.24936225	3
2021-02-15-09-00-IB100p	A	Dry	09	1.02207094	2.16491	0.705248	6.66240967	0.82000724	0.98921812	0.82894482	0.14030612	4
2021-02-19-12-00-IB100p	A	Dry	12	1.07114248	2.268314	0.855637	5.32589379	0.8997419	0.97632584	0.92155904	0.12468112	3
2021-03-05-18-12-IB100p	A	Dry	18	1.04144067	1.559553	0.365298	9.08332579	0.77719843	0.97557133	0.79665977	0.25	1
2021-03-11-14-00-IB100p	A	Dry	14	1.04150527	2.254225	0.83819	9.0816277	0.8900962	0.98794767	0.9009548	0.49744898	2
2021-03-16-18-30-IB100p	A	Dry	18	1.02320081	1.175888	0.264419	6.48354694	0.82218504	0.98926314	0.83110853	0.15784439	0
2021-03-29-17-36-IB100p	A	Dry	17	1.17090768	2.183185	0.727962	6.77293502	0.85704615	0.96655868	0.88669852	0.05580357	2
2021-04-03-16-06-IB100p	A	Dry	16	1.03815852	2.20948	0.757468	3.66961282	0.8202431	0.9367898	0.87558927	0.00318878	3
2021-04-04-18-54-IB100p	A	Dry	18	1.06791947	1.444732	0.336212	10.8279635	0.76356739	0.95614575	0.79858891	0.08609694	0
2021-04-22-15-42-IB100p	A	Dry	15	1.03607017	1.794436	0.462613	7.07162804	0.78450956	0.96688267	0.81138032	0.10044643	4
2021-04-27-13-48-IB100p	A	Dry	13	1.14561346	2.103239	0.66567	9.32871464	0.86743384	0.97756209	0.88734399	0.0908801	2
2021-05-07-06-12-IB100p	A	Wet	06	1.10555664	1.855234	0.523851	11.0785184	0.78420889	0.97034498	0.80817534	0.22799745	5
2021-05-21-17-30-IB100p	A	Wet	17	1.01580892	1.329709	0.304891	7.8026353	0.78775368	0.95574718	0.8242281	0.02072704	3

2021-05-23-15-48-IB100p	A	Wet	15	1.03500442	2.275388	0.878022	10.5602937	0.816226	0.98942644	0.82494864	0.65816327	2
2021-06-01-10-24-IB100p	A	Wet	10	1.02717869	2.117256	0.666255	6.03967458	0.87738168	0.96948533	0.90499737	0.20280612	2
2021-06-29-11-42-IB100p	A	Wet	11	1.01224551	2.106696	0.650197	6.75676701	0.78509891	0.98926441	0.79361887	0.38137755	3
2021-07-08-15-36-IB100p	A	Wet	15	1.03914664	1.721844	0.441879	5.73355608	0.86753863	0.98105721	0.88428954	0.07653061	1
2021-07-13-17-00-IB100p	A	Wet	17	1.05704608	2.258427	0.854479	14.4752313	0.84032977	0.971952	0.8645795	0.25	5
2021-07-15-18-54-IB100p	A	Wet	18	1.02013426	2.00188	0.580112	8.44250008	0.80709556	0.98878012	0.81625383	0.18303571	0
2021-08-04-07-12-IB100p	A	Wet	07	1.02038333	2.26701	0.889177	4.0624612	0.89124173	0.984704	0.90508593	0.43048469	2
2021-08-05-05-00-IB100p	A	Wet	05	1.0104704	2.284054	0.896076	5.46542277	0.78464357	0.99136335	0.7914793	0.375	2
2021-08-16-11-54-IB100p	A	Wet	11	1.05514734	1.940849	0.587831	5.37308553	0.83103507	0.94970333	0.87504703	0.00892857	6
2021-08-18-06-48-IB100p	A	Wet	06	1.00177051	1.921101	0.525507	6.56150402	0.74607725	0.99107767	0.75279393	0.79209184	7
2021-08-27-12-18-IB100p	A	Wet	12	1.01044784	1.196517	0.279773	7.38911001	0.83433002	0.97357132	0.85697884	0.01434949	3
2021-09-06-14-42-IB100p	A	Wet	14	1.08468506	2.290957	0.916706	7.22537341	0.89952734	0.98684689	0.91151661	0.08609694	3
2021-09-13-05-30-IB100p	A	Wet	05	1.01755998	2.142112	0.710815	9.11819143	0.84928938	0.98685856	0.86059889	0.18781888	4
2021-09-16-07-48-IB100p	A	Wet	07	1.00669198	1.6273	0.419533	3.99815733	0.79945545	0.98521014	0.81145678	0.18941327	4
2021-09-26-13-42-IB100p	A	Wet	13	1.01467957	2.298344	0.947932	6.80700119	0.89359852	0.989279	0.90328261	0.08992347	2
2021-10-18-14-06-IB100p	A	Wet	14	1.01779542	1.21001	0.25767	4.94239035	0.7494657	0.98916273	0.75767685	0.45248725	2
2021-10-22-06-54-IB100p	A	Wet	06	1.0186682	1.279281	0.275279	7.90424153	0.76955714	0.9877364	0.77911186	0.33394452	3
2020-09-11-09-54-IB100p	A	Wet	09	1.00976493	2.302584	0.999172	2.91044967	0.94747208	0.99139824	0.95569272	0.0208239	0
2020-11-02-10-18-IB100p	A	Dry	10	1.02777122	2.30027	0.962308	1.91851153	0.9050639	0.98749706	0.91652314	0.01539158	5
2020-11-06-17-00-IB100p	A	Dry	17	1.01859321	1.129939	0.25964	3.58787158	0.85966557	0.98205371	0.87537531	0.04074242	1
2020-11-16-10-00-IB100p	A	Dry	10	1.02418527	2.288017	0.904128	2.78903838	0.88491858	0.98812702	0.89555145	0.03440471	3
2020-11-25-15-12-IB100p	A	Dry	15	1.0249477	2.286433	0.898675	3.31533244	0.83491663	0.98995512	0.84338837	0.18469896	0
2020-12-04-07-48-IB100p	A	Dry	07	1.01910516	1.302181	0.296038	4.35683718	0.80699105	0.98768437	0.81705358	0.11407877	5
2021-01-12-17-30-IB100p	A	Dry	17	1.0126954	2.301174	0.97041	2.64726799	0.82257629	0.99128334	0.82980945	0.55771843	2
2021-02-16-09-18-IB100p	A	Dry	09	1.02187064	2.20314	0.754305	2.36840986	0.87773754	0.98632864	0.88990374	0.06337709	7
2021-03-04-12-00-IB100p	A	Dry	12	1.02704284	1.610904	0.396422	2.32152258	0.86882337	0.98400279	0.88294807	0.04481666	3
2021-03-10-12-30-IB100p	A	Dry	12	1.04663415	1.883462	0.515111	4.61444113	0.85604535	0.96821163	0.88415108	0.00995926	0
2021-03-15-07-24-IB100p	A	Dry	07	1.02501912	2.127925	0.671596	6.66527576	0.80378113	0.98738398	0.81405121	0.24626528	4

2021-03-26-05-00-IB100p	A	Dry	05	1.03615784	2.20827	0.784267	10.1317216	0.76533786	0.9912854	0.77206611	0.59076505	2
2021-04-16-05-30-IB100p	A	Dry	05	1.05151693	2.287331	0.902903	7.62962675	0.85493263	0.99015247	0.86343533	0.29425079	6
2021-05-15-12-18-IB100p	A	Wet	12	1.00159077	2.299011	0.953406	4.58043168	0.87901481	0.9900273	0.88786926	0.20371209	3
2021-05-18-08-00-IB100p	A	Wet	08	1.04928932	2.256393	0.833699	8.53460429	0.83464753	0.98445618	0.84782598	0.21729289	0
2021-05-27-16-12-IB100p	A	Wet	16	1.02581301	2.104248	0.657234	4.75137732	0.83976589	0.98634879	0.85138838	0.15617927	3
2021-06-08-14-18-IB100p	A	Wet	14	1.03060306	2.302585	0.99954	1.52176957	0.96077808	0.99443052	0.96615908	1	0
2021-06-09-14-42-IB100p	A	Wet	14	1.00683394	2.301777	0.977066	2.24891557	0.8689038	0.99024732	0.8774614	0.30421005	2
2021-06-11-14-36-IB100p	A	Wet	14	1.00228424	2.286693	0.899411	3.37831006	0.86717508	0.9906904	0.87532399	0.31824355	4
2021-06-15-16-18-IB100p	A	Wet	16	1.01826535	2.229249	0.797813	4.9605024	0.88153873	0.98914039	0.89121701	0.19013128	2
2021-06-22-08-54-IB100p	A	Wet	08	1.00321871	2.298644	0.949767	5.24719606	0.87884262	0.99019016	0.88754934	0.33001358	4
2021-06-28-06-18-IB100p	A	Wet	06	1.01096454	2.281524	0.892953	7.40975413	0.82407794	0.98296789	0.83835692	0.14938886	4
2021-07-10-06-18-IB100p	A	Wet	06	1.00592349	2.21837	0.785542	9.21686212	0.80669095	0.98295173	0.82068217	0.1466727	4
2021-07-27-11-36-IB100p	A	Wet	11	1.06459802	2.264248	0.844115	3.03579235	0.86194299	0.98280427	0.87702405	0.12041648	2
2021-08-05-13-48-IB100p	A	Wet	13	1.03121957	2.30199	0.980632	5.01214055	0.91366273	0.9905291	0.92239867	0.63558171	0
2021-09-13-06-54-IB100p	A	Wet	06	1.00368743	2.29211	0.919413	3.98426085	0.88528053	0.99082918	0.89347443	0.22951562	5
2021-10-15-18-12-IB100p	A	Wet	18	1.09632718	2.301373	0.975059	6.40051746	0.83047033	0.98864062	0.84001235	0.31688547	0
2021-10-19-18-06-IB100p	A	Wet	18	1.01583677	1.392805	0.305822	7.04497616	0.82946797	0.9812845	0.84528796	0.04753282	1
2021-10-29-13-18-IB100p	A	Wet	13	1.01542092	2.300632	0.964853	3.16423616	0.79485675	0.99120094	0.80191283	0.58397465	0
2022-01-04-15-00-IB100p	A	Dry	15	1.02059632	2.302367	0.989057	2.20796877	0.89191059	0.9895462	0.90133294	0.11453146	1
2022-01-05-18-54-IB100p	A	Dry	18	1.03230846	2.295866	0.9416	9.46246302	0.80974293	0.99036908	0.81761734	0.42009959	0
2022-01-15-08-18-IB100p	A	Dry	08	1.02981771	2.188	0.739863	2.91962798	0.89541746	0.98020033	0.91350455	0.02172929	3
2022-01-29-13-54-IB100p	A	Dry	13	1.0204694	2.297279	0.943133	3.05472175	0.88439112	0.98941544	0.89385215	0.02987777	5
2022-01-31-11-00-IB100p	A	Dry	11	1.01708827	2.302299	0.986556	2.21114334	0.88332575	0.99092541	0.89141498	0.11407877	6
2021-07-28-12-36-IB100p	A	Wet	12	1.07933006	2.284758	0.899529	3.34171676	0.88360187	0.986572	0.89562837	0.25	1
2021-08-08-14-30-IB100p	A	Wet	14	1.03079013	1.745635	0.464805	1.87102314	0.79254972	0.94264248	0.84077446	0.015625	1
2021-08-19-18-00-IB100p	A	Wet	18	1.00831719	2.302483	0.992307	3.32035729	0.8415854	0.99037164	0.84976727	0.38671875	1
2020-10-02-10-18-IB100p	A	Wet	10	1.01098552	2.302584	0.999172	2.96941298	0.94794798	0.99135268	0.95621669	0.02379536	0
2020-10-04-11-06-IB100p	A	Wet	11	1.05701365	2.207564	0.761226	8.64953061	0.86371512	0.96904961	0.89130124	0.00535396	5

2020-11-06-10-48-IB100p	A	Dry	10	1.0956883	2.1508	0.704352	1.6723254	0.85815042	0.98991319	0.86689462	0.35574063	1
2020-11-27-05-06-IB100p	A	Dry	05	1.08268447	2.202463	0.766886	6.49815198	0.76660762	0.98191074	0.78073045	0.17132659	0
2021-01-01-05-12-IB100p	A	Dry	05	1.02122913	2.021804	0.618516	9.9026624	0.77900374	0.99080629	0.78623213	0.2082094	1
2021-01-28-06-30-IB100p	A	Dry	06	1.02605386	2.245567	0.810518	3.4699669	0.78672952	0.99116381	0.79374319	0.6728138	2
2021-02-05-07-00-IB100p	A	Dry	07	1.03033225	2.247134	0.815039	3.7993727	0.8478996	0.99001026	0.85645536	0.14455681	6
2021-02-05-08-30-IB100p	A	Dry	08	1.14675593	1.889286	0.536219	3.5015967	0.871708	0.9743098	0.89469284	0.1189768	3
2021-02-15-08-54-IB100p	A	Dry	08	1.01757264	2.26693	0.850808	2.08729173	0.87076209	0.98926269	0.88021321	0.10469958	4
2021-02-16-13-24-IB100p	A	Dry	13	1.03592654	1.454888	0.343688	3.69184831	0.79410936	0.93455472	0.84971949	0.0035693	3
2021-02-20-15-54-IB100p	A	Dry	15	1.0481144	1.701082	0.418061	3.63990348	0.86513901	0.96887549	0.89293105	0.00594884	3
2021-02-22-17-12-IB100p	A	Dry	17	1.04623693	2.302344	0.987637	2.02445237	0.86183478	0.9906213	0.8699942	0.4907793	2
2021-03-09-11-06-IB100p	A	Dry	11	1.01853495	2.258544	0.832778	2.6679854	0.85464098	0.99044311	0.86288751	0.40571089	2
2021-03-17-16-12-IB100p	A	Dry	16	1.01065332	1.530447	0.353615	2.67579126	0.87533628	0.98364678	0.88988883	0.0333135	4
2021-03-19-11-24-IB100p	A	Dry	11	1.02732506	2.301882	0.979073	3.19695637	0.87002079	0.99103432	0.87789169	0.66032124	4
2021-04-04-16-00-IB100p	A	Dry	16	1.03514357	2.302555	0.996129	2.40662217	0.93487141	0.98479412	0.94930645	0.24092802	2
2021-04-07-09-24-IB100p	A	Dry	09	1.0156019	2.301703	0.97667	2.07561986	0.88928373	0.9893609	0.89884665	0.09577632	5
2021-05-13-13-36-IB100p	A	Wet	13	1.00726908	2.257078	0.832645	5.50522927	0.86107363	0.99009847	0.86968484	0.51635931	4
2021-05-25-18-54-IB100p	A	Wet	18	1.06248749	2.301982	0.981365	6.61424778	0.8773429	0.98828553	0.88774233	0.13087448	0
2021-06-19-16-42-IB100p	A	Wet	16	1.00739593	2.275888	0.871532	3.35152458	0.89513435	0.98804866	0.90596181	0.12849494	2
2021-06-22-15-48-IB100p	A	Wet	15	0.99391874	2.273057	0.865399	4.5139287	0.8773352	0.9881943	0.8878165	0.38429506	4
2021-06-25-09-00-IB100p	A	Wet	09	1.09678555	2.278941	0.88264	11.3152415	0.81298835	0.99049494	0.82079001	0.78048781	4
2021-06-26-07-42-IB100p	A	Wet	07	1.09185815	2.301796	0.978587	7.18118849	0.84634887	0.99032844	0.85461432	0.69601428	2
2021-07-15-17-18-IB100p	A	Wet	17	1.01550901	2.289741	0.921612	12.1549067	0.83712625	0.97995679	0.85424812	0.22903034	2
2021-08-28-05-24-IB100p	A	Wet	05	1.00967581	2.29433	0.935342	8.02784061	0.81682664	0.98906956	0.82585358	0.41225461	1
2021-08-28-07-18-IB100p	A	Wet	07	1.02150259	2.087803	0.640815	7.39480634	0.85530036	0.98211668	0.87087449	0.20107079	4
2021-08-28-12-30-IB100p	A	Wet	12	1.0393064	2.302564	0.997317	2.27582021	0.91046293	0.98603611	0.92335657	0.34265318	1
2021-11-01-14-30-IB100p	A	Dry	14	1.02175649	2.299324	0.955754	2.68920925	0.86104741	0.99097647	0.86888784	0.40690065	0
2021-11-03-15-54-IB100p	A	Dry	15	1.01065539	2.300133	0.961056	3.20831131	0.82816885	0.99152362	0.83524874	0.78048781	0
2021-11-08-17-48-IB100p	A	Dry	17	1.03671217	2.294394	0.931956	3.84111719	0.84636188	0.98762768	0.85696452	0.28316478	0

2021-12-26-10-18-IB100p	A	Dry	10	1.01979368	2.299393	0.954509	4.02798004	0.89549179	0.98940642	0.90507983	0.02855443	4
2022-01-16-06-42-IB100p	A	Dry	06	1.0068233	2.294752	0.92919	4.91948091	0.82613865	0.99105543	0.8335948	0.47471743	3
2022-01-30-09-48-IB100p	A	Dry	09	1.01494438	1.845916	0.486454	4.11475554	0.83140335	0.96909208	0.85791988	0.05993456	5
2020-11-01-05-24-IB100p	A	Dry	05	1.01816666	0.665827	0.165217	5.32549757	0.81944013	0.97894121	0.83706776	0.01712247	0
2020-11-01-16-42-IB100p	A	Dry	16	1.07707113	2.27873	0.880089	2.49266576	0.88504023	0.97339591	0.90922945	0.10273484	0
2020-11-02-11-12-IB100p	A	Dry	11	1.02367643	2.302338	0.987588	2.62271221	0.91697844	0.9900659	0.92617919	0.01469679	3
2020-11-06-12-12-IB100p	A	Dry	12	1.02158407	1.74392	0.435261	2.22771737	0.85565487	0.98288451	0.87055484	0.05707491	2
2020-11-10-12-18-IB100p	A	Dry	12	1.01649548	2.302461	0.991096	2.23618343	0.9089584	0.99095118	0.91725851	0.03709869	2
2020-11-14-08-00-IB100p	A	Dry	08	1.03697692	2.266093	0.862008	3.87649954	0.87684638	0.98231721	0.89263058	0.10573127	5
2020-11-14-10-00-IB100p	A	Dry	10	1.03462644	2.294287	0.931348	1.71225995	0.87459309	0.98601701	0.88699595	0.14387634	3
2020-11-16-08-00-IB100p	A	Dry	08	1.02212575	2.038559	0.599151	3.52035231	0.88172189	0.98130527	0.89851947	0.05160523	3
2020-11-19-07-00-IB100p	A	Dry	07	1.0420083	1.570366	0.369091	4.09703152	0.82676967	0.9510619	0.86931216	0.0019025	4
2020-11-21-11-42-IB100p	A	Dry	11	1.01703382	2.301737	0.978082	1.50105251	0.86479391	0.99021918	0.87333586	0.11985731	1
2020-11-21-13-12-IB100p	A	Dry	13	1.02108915	1.866151	0.497632	1.84957613	0.84039916	0.96872288	0.8675331	0.01065398	1
2020-11-21-18-36-IB100p	A	Dry	18	1.10560018	2.23842	0.823715	9.9410202	0.82265038	0.98588276	0.83443023	0.159239	0
2020-11-26-16-24-IB100p	A	Dry	16	1.01991004	0.699476	0.166605	2.81996637	0.81311283	0.96988393	0.83836097	0.05174792	1
2020-11-30-15-24-IB100p	A	Dry	15	1.01659337	2.301042	0.969205	1.33731284	0.8950645	0.98891529	0.90509724	0.13902497	1
2020-12-01-08-00-IB100p	A	Dry	08	1.04469163	1.676536	0.440223	2.1921969	0.83500294	0.96383617	0.86633286	0.03376932	3
2020-12-02-11-00-IB100p	A	Dry	11	1.02930319	1.73653	0.433292	2.27659115	0.8437858	0.9872453	0.85468708	0.15334126	1
2020-12-04-13-42-IB100p	A	Dry	13	1.01522691	0.371889	0.124603	1.94813349	0.84257826	0.97936344	0.86033257	0.06340071	1
2020-12-07-10-30-IB100p	A	Dry	10	1.1208244	2.301159	0.969776	1.84741459	0.87979829	0.9899352	0.88874332	0.38049941	0
2020-12-07-12-42-IB100p	A	Dry	12	1.04421919	2.301568	0.975241	0.99656017	0.91169224	0.9879728	0.92279084	0.02197384	1
2020-12-09-15-06-IB100p	A	Dry	15	1.11629806	2.295182	0.933939	1.93658321	0.90182193	0.980995	0.91929309	0.16480381	0
2020-12-18-15-24-IB100p	A	Dry	15	1.02022983	0.977046	0.208487	2.4605363	0.79632603	0.94169341	0.84563195	0.00185494	2
2020-12-18-16-54-IB100p	A	Dry	16	1.03738554	2.30241	0.989574	2.06186029	0.91002476	0.98157106	0.92711042	0.07229489	1
2020-12-22-07-30-IB100p	A	Dry	07	1.05092701	1.866623	0.527075	3.56552951	0.85903314	0.96864863	0.88683668	0.04494649	5
2020-12-25-12-48-IB100p	A	Dry	12	1.01855382	2.301906	0.980178	2.19217007	0.90389141	0.99054444	0.91251979	0.09136742	2
2020-12-26-09-42-IB100p	A	Dry	09	1.03441234	2.30221	0.98469	2.06873159	0.91217106	0.98820082	0.92306245	0.03662307	3

2020-12-26-18-06-IB100p	A	Dry	18	1.0581605	2.302549	0.995615	2.58511743	0.87761968	0.99042213	0.88610669	0.26439952	0
2021-01-27-07-18-IB100p	A	Dry	07	1.02490111	1.98944	0.575046	2.53152645	0.85240065	0.98945568	0.86148442	0.37222354	4
2021-02-07-14-00-IB100p	A	Dry	14	1.02396552	2.3012	0.970653	1.89149046	0.88398747	0.98995494	0.89295728	0.13931035	2
2021-02-11-08-00-IB100p	A	Dry	08	1.07373686	2.101644	0.648436	4.08760524	0.84560943	0.98645281	0.85722239	0.22373365	3
2021-02-12-09-18-IB100p	A	Dry	09	1.02300103	2.297621	0.945252	1.76675953	0.90292975	0.98833964	0.91358244	0.00694411	3
2021-02-12-12-30-IB100p	A	Dry	12	1.02142167	2.289723	0.920691	2.55311959	0.92927203	0.98683546	0.94166866	0.00285375	7
2021-02-13-09-54-IB100p	A	Dry	09	1.14151528	2.294874	0.933149	1.7529355	0.91002413	0.97482064	0.93352981	0.11533888	2
2021-02-15-14-36-IB100p	A	Dry	14	1.00740961	2.302087	0.982265	5.34282105	0.89649069	0.97710502	0.91749677	0.16646849	0
2021-02-23-10-06-IB100p	A	Dry	10	1.03967922	2.270911	0.873141	2.01045046	0.90011584	0.98813177	0.91092693	0.07966706	3
2021-02-26-18-36-IB100p	A	Dry	18	1.03444183	1.236686	0.262326	6.11133267	0.81783108	0.96355425	0.84876495	0.03210464	0
2021-03-02-07-54-IB100p	A	Dry	07	1.0072857	2.294327	0.928815	1.88445535	0.80190087	0.98986832	0.81010863	0.34844233	6
2021-03-03-09-24-IB100p	A	Dry	09	1.03266672	2.300087	0.95987	4.10272558	0.87145423	0.99105621	0.87931866	0.67686088	2
2021-03-06-11-30-IB100p	A	Dry	11	1.02428377	2.302231	0.98513	1.70092648	0.86475276	0.99071566	0.87285666	0.20808561	5
2021-03-10-06-42-IB100p	A	Dry	06	1.0183923	2.188949	0.732767	3.04212215	0.79333517	0.99120227	0.80037667	0.55219976	6
2021-03-10-08-24-IB100p	A	Dry	08	1.02663657	2.299691	0.960739	4.96497057	0.88530322	0.99062664	0.89368001	0.45398335	3
2021-03-12-14-36-IB100p	A	Dry	14	1.02340424	2.298416	0.948645	2.59608097	0.8692688	0.99068007	0.87744654	0.53821641	1
2021-03-16-15-06-IB100p	A	Dry	15	1.04857828	2.3009	0.967422	2.32484952	0.88925606	0.99022016	0.89803874	0.32770511	1
2021-03-17-07-54-IB100p	A	Dry	07	1.04412777	2.008362	0.583635	2.04786488	0.87368344	0.97357032	0.89740147	0.08689655	4
2021-03-17-18-00-IB100p	A	Dry	18	1.0719114	1.697814	0.426824	6.82785635	0.78048542	0.96472545	0.80902335	0.05545779	2
2021-03-20-16-18-IB100p	A	Dry	16	1.03380726	1.372999	0.303093	4.07455491	0.7979516	0.95252324	0.83772402	0.01450654	2
2021-03-21-09-00-IB100p	A	Dry	09	1.02561107	2.135785	0.678858	3.01300117	0.85190258	0.9895978	0.86085739	0.08494649	6
2021-03-25-08-06-IB100p	A	Dry	08	1.03190506	2.29555	0.934328	2.31823408	0.89598664	0.98660118	0.90815484	0.21517241	5
2021-03-27-05-48-IB100p	A	Dry	05	1.07155202	2.250964	0.819874	7.00619351	0.78913944	0.99097916	0.79632294	0.62154578	3
2021-03-30-05-48-IB100p	A	Dry	05	1.06912824	2.124097	0.668445	5.81583993	0.80198505	0.98865553	0.81118754	0.35424495	4
2021-03-30-08-36-IB100p	A	Dry	08	1.0223928	2.282172	0.894873	2.76769393	0.87687691	0.98984302	0.88587472	0.38221165	3
2021-03-30-12-24-IB100p	A	Dry	12	1.02892896	2.206801	0.760729	2.83794915	0.92527703	0.98732363	0.93715677	0.06891796	3
2021-04-01-14-42-IB100p	A	Dry	14	1.03563889	1.553711	0.36859	2.33582731	0.83962955	0.93772967	0.89538549	0.000761	4
2021-04-03-12-42-IB100p	A	Dry	12	1.0288228	2.228739	0.782739	3.10641451	0.90478208	0.98378612	0.91969389	0.00837099	4

2021-04-07-13-18-IB100p	A	Dry	13	1.03210348	1.449663	0.338844	3.59593714	0.78090508	0.93206062	0.83782649	0.00133175	4
2021-04-10-10-42-IB100p	A	Dry	10	1.01818587	2.302272	0.986439	1.99495834	0.89865581	0.98516665	0.91218659	0.09750297	2
2021-04-11-16-00-IB100p	A	Dry	16	1.03256643	2.283782	0.891567	0.90841606	0.90571076	0.97401054	0.92987778	0.02406659	2
2021-04-14-16-48-IB100p	A	Dry	16	1.01246746	2.300373	0.96329	2.24603501	0.88078065	0.98933223	0.89027793	0.05992866	3
2021-04-16-06-54-IB100p	A	Dry	06	1.028765	1.762236	0.44479	6.7380294	0.82361064	0.96644317	0.85220804	0.0587396	4
2021-04-17-07-12-IB100p	A	Dry	07	1.01053974	2.277435	0.878566	7.25133188	0.83521013	0.98954967	0.84403053	0.41550535	6
2021-04-18-05-12-IB100p	A	Dry	05	1.02025992	2.251029	0.823365	6.59385502	0.85182805	0.9904534	0.86003849	0.27980975	2
2021-04-19-15-48-IB100p	A	Dry	15	1.02960808	1.793257	0.494072	1.58799801	0.83621044	0.95884695	0.87210002	0.00865636	2
2021-04-22-10-06-IB100p	A	Dry	10	1.01433291	1.706724	0.416788	3.03349935	0.87754407	0.97487872	0.90015717	0.03338882	4
2021-04-29-15-00-IB100p	A	Dry	15	1.05432907	2.26032	0.843293	2.15881559	0.90879133	0.98151186	0.92590968	0.18302021	4
2021-05-02-06-06-IB100p	A	Wet	06	1.01726471	2.274499	0.869099	2.87465673	0.87157412	0.98957062	0.8807599	0.17250892	5
2021-05-13-15-06-IB100p	A	Wet	15	1.04833098	2.033849	0.602844	7.58801765	0.82245172	0.96104059	0.85579291	0.04147444	2
2021-05-16-11-54-IB100p	A	Wet	11	1.05637698	1.866896	0.506136	6.95331621	0.82884748	0.97397497	0.85099464	0.1297503	0
2021-05-17-13-00-IB100p	A	Wet	13	1.016693	2.059456	0.620321	5.93313477	0.82872367	0.9772788	0.84799105	0.13931035	0
2021-05-18-07-54-IB100p	A	Wet	07	1.06578494	2.297597	0.946737	6.03384346	0.85939786	0.98930321	0.86869006	0.54872771	0
2021-05-18-14-36-IB100p	A	Wet	14	1.02391034	2.258269	0.834286	7.47554716	0.8236459	0.96669086	0.85202616	0.07790725	4
2021-05-21-07-54-IB100p	A	Wet	07	1.06156342	2.279041	0.887257	7.69509636	0.84983216	0.99293874	0.85587573	0.97260404	0
2021-05-23-18-30-IB100p	A	Wet	18	1.02378848	2.297725	0.944469	4.12604654	0.90087178	0.98972567	0.91022372	0.1626635	2
2021-05-27-06-42-IB100p	A	Wet	06	1.04919948	2.265043	0.865569	11.7552772	0.8327533	0.99397147	0.83780402	0.98625446	1
2021-05-28-10-54-IB100p	A	Wet	10	1.00822908	2.281456	0.887738	6.53585268	0.84341985	0.98760621	0.8540042	0.36223543	4
2021-05-30-14-00-IB100p	A	Wet	14	1.05451434	2.239448	0.807953	7.66096035	0.8338519	0.98353715	0.84780925	0.26996433	2
2021-06-03-10-54-IB100p	A	Wet	10	1.07834656	2.296631	0.940476	9.07971634	0.83943127	0.98844333	0.84924573	0.47162901	2
2021-06-06-06-48-IB100p	A	Wet	06	1.04312141	2.2727	0.876543	11.4879887	0.84361097	0.99422878	0.8485079	1	2
2021-06-08-07-24-IB100p	A	Wet	07	1.06382362	2.299632	0.959093	6.41144364	0.88273432	0.99260296	0.8893126	0.95904875	1
2021-06-09-17-18-IB100p	A	Wet	17	1.01794841	2.30248	0.991941	2.10316699	0.90648397	0.99097802	0.91473671	0.20561237	3
2021-06-11-16-54-IB100p	A	Wet	16	1.11252361	2.30258	0.998447	1.84823373	0.91951144	0.98825774	0.93043687	0.24313912	3
2021-06-12-09-12-IB100p	A	Wet	09	1.01102971	2.0379	0.618576	8.40404071	0.82639067	0.99028302	0.83449948	0.65141498	4
2021-06-13-06-42-IB100p	A	Wet	06	1.17149877	2.215501	0.803843	12.4835149	0.80169585	0.99145851	0.80860252	0.89250892	4

2021-06-17-17-18-IB100p	A	Wet	17	1.02582047	2.259466	0.836519	2.86287451	0.9070885	0.98447698	0.92139127	0.12965517	3
2021-06-18-14-48-IB100p	A	Wet	14	1.0157955	2.259485	0.838762	7.04250625	0.8517571	0.98098175	0.86827008	0.1932937	5
2021-06-20-05-30-IB100p	A	Wet	05	1.09444455	2.23463	0.814203	12.8068885	0.80384655	0.99069731	0.8113947	0.82568371	3
2021-06-23-07-00-IB100p	A	Wet	07	1.14302843	2.196597	0.778993	12.8139745	0.80383188	0.99181314	0.81046706	0.93888228	4
2021-07-03-14-24-IB100p	A	Wet	14	1.2083372	2.302574	0.997804	2.14501542	0.92624198	0.98549257	0.93987719	0.27205708	0
2021-07-04-16-48-IB100p	A	Wet	16	1.01594528	2.300442	0.966469	1.8160444	0.92154419	0.98899917	0.93179471	0.0616409	0
2021-07-08-09-00-IB100p	A	Wet	09	1.14774738	2.292865	0.9235	5.44306822	0.83724585	0.99137553	0.84452948	0.89921522	1
2021-07-13-06-06-IB100p	A	Wet	06	0.99883496	2.090112	0.65487	5.86502768	0.83885043	0.98474636	0.85184415	0.20741974	4
2021-07-16-15-30-IB100p	A	Wet	15	1.05687553	2.288559	0.929077	14.1336774	0.85960698	0.98870107	0.86943061	0.4870868	1
2021-07-17-06-48-IB100p	A	Wet	06	1.01452011	2.294951	0.933261	7.47604794	0.82335015	0.98532191	0.83561539	0.27167658	3
2021-07-18-13-06-IB100p	A	Wet	13	1.02195241	2.211209	0.765757	7.56686706	0.85005064	0.97162988	0.87487084	0.07319857	6
2021-07-19-06-00-IB100p	A	Wet	06	1.12197996	2.302567	0.996922	2.11890142	0.89162685	0.98829615	0.9021859	0.3219025	3
2021-07-21-12-12-IB100p	A	Wet	12	1.11814928	2.302571	0.997611	2.24376102	0.92255322	0.9894702	0.9323709	0.50482759	1
2021-07-23-18-12-IB100p	A	Wet	18	1.1054008	2.302452	0.990919	4.07871297	0.90675403	0.99116967	0.9148323	0.87952438	0
2021-07-24-05-00-IB100p	A	Wet	05	1.13639852	2.302569	0.997837	2.21382085	0.92331765	0.98884789	0.93373072	0.45631391	0
2021-07-25-06-48-IB100p	A	Wet	06	1.15161764	2.302422	0.990885	1.31996395	0.92435713	0.98461699	0.93879868	0.29678954	1
2021-07-25-08-12-IB100p	A	Wet	08	1.1212735	2.30255	0.997088	1.72321702	0.92245609	0.98894921	0.93276387	0.47942925	2
2021-07-25-09-00-IB100p	A	Wet	09	1.15395221	2.302551	0.995756	1.82381138	0.92332523	0.98712454	0.93536853	0.35105826	1
2021-07-25-17-06-IB100p	A	Wet	17	1.07794983	2.29839	0.958464	12.4018883	0.84902336	0.99121916	0.85654455	0.87971463	1
2021-07-27-10-30-IB100p	A	Wet	10	1.08746333	2.291427	0.916111	2.38692696	0.90996151	0.98316451	0.92554348	0.2164566	2
2021-08-01-16-48-IB100p	A	Wet	16	1.01909716	2.092442	0.662491	2.08098711	0.85666217	0.96551856	0.88725603	0.04337693	2
2021-08-01-17-54-IB100p	A	Wet	17	1.00798428	2.293297	0.924637	2.49663062	0.87769263	0.99003971	0.88652266	0.32989299	0
2021-08-02-11-18-IB100p	A	Wet	11	1.06100446	2.171049	0.730084	2.14840645	0.80480902	0.97108494	0.82877304	0.10102259	2
2021-08-03-05-00-IB100p	A	Wet	05	1.18518835	2.302569	0.997014	1.6466633	0.92055301	0.9866439	0.93301445	0.28623068	0
2021-08-06-05-00-IB100p	A	Wet	05	1.05896858	2.297376	0.950501	7.76986236	0.85394774	0.98786334	0.86443914	0.24423306	0
2021-08-12-08-18-IB100p	A	Wet	08	1.01277061	2.299003	0.953389	5.03866012	0.8715589	0.97698029	0.89209466	0.13227111	4
2021-08-21-17-06-IB100p	A	Wet	17	1.08652528	2.302301	0.986789	2.24206926	0.91682083	0.98978257	0.92628508	0.60437574	0
2021-08-24-16-12-IB100p	A	Wet	16	1.05157603	2.302544	0.996116	2.30043725	0.91543987	0.99074601	0.92399047	0.68917955	1

2021-08-24-18-42-IB100p	A	Wet	18	1.05196444	2.302383	0.990143	2.85410542	0.91119282	0.9895273	0.92083646	0.17778835	0
2021-08-28-11-30-IB100p	A	Wet	11	1.02074815	2.13649	0.711331	8.27213594	0.83649043	0.98100597	0.85268638	0.18758621	1
2021-08-29-18-30-IB100p	A	Wet	18	1.02511541	2.301538	0.976736	4.82410977	0.90169985	0.99018456	0.91063817	0.15828775	1
2021-09-03-13-30-IB100p	A	Wet	13	1.03714918	2.291949	0.920198	2.05383665	0.90631762	0.98378981	0.92125128	0.11557669	0
2021-09-08-17-42-IB100p	A	Wet	17	1.03879038	2.302583	0.998778	2.64003012	0.91864943	0.99038938	0.92756389	0.2568371	1
2021-09-23-13-06-IB100p	A	Wet	13	1.03077109	2.302557	0.995808	2.07140222	0.91133811	0.98650048	0.92380909	0.30906064	1
2021-09-25-08-06-IB100p	A	Wet	08	1.00000891	2.260709	0.847002	5.69551827	0.88699399	0.99087982	0.89515799	0.42615933	5
2021-09-25-15-24-IB100p	A	Wet	15	1.00953315	2.283226	0.889373	3.26305111	0.75049528	0.99192273	0.7566066	0.86511296	0
2021-09-27-14-12-IB100p	A	Wet	14	0.99956773	2.302482	0.992258	3.25819208	0.90564903	0.99133685	0.91356336	0.24760999	5
2021-09-27-17-12-IB100p	A	Wet	17	1.00124207	2.250906	0.818043	3.36696715	0.8977709	0.99062459	0.90626753	0.39581451	2
2021-09-30-05-18-IB100p	A	Wet	05	1.05762234	2.302567	0.997835	2.36432313	0.91230066	0.98912839	0.92232786	0.44071344	0
2021-10-01-13-06-IB100p	A	Wet	13	1.01636255	1.355755	0.323659	3.33077336	0.86259854	0.9740768	0.88555496	0.07648038	0
2021-10-05-09-54-IB100p	A	Wet	09	1.06369288	2.301501	0.973644	2.48794212	0.90836156	0.98556959	0.92166152	0.30249703	0
2021-10-15-12-00-IB100p	A	Wet	12	1.01321445	2.298319	0.94741	2.46509373	0.84469541	0.9905235	0.85277675	0.18111772	2
2021-10-18-12-48-IB100p	A	Wet	12	1.0226313	0.88375	0.200921	3.23921588	0.76680734	0.95594311	0.80214747	0.01883472	1
2021-10-24-17-42-IB100p	A	Wet	17	1.0174575	2.301702	0.976426	7.34961142	0.85299553	0.99044536	0.86122422	0.44470868	1
2021-10-25-10-48-IB100p	A	Wet	10	1.03263355	2.302399	0.989049	2.30319346	0.86743022	0.9909241	0.87537504	0.47243757	3
2022-01-01-05-24-IB100p	A	Dry	05	1.00992955	2.105563	0.661729	6.20957918	0.80201238	0.98813192	0.81164505	0.28461356	0
2022-01-02-17-54-IB100p	A	Dry	17	1.00265588	2.295867	0.935038	7.27685113	0.84682944	0.99075745	0.85472931	0.56175981	1
2022-01-04-14-30-IB100p	A	Dry	14	1.03346109	2.302537	0.994409	2.26155129	0.90012113	0.98977445	0.90942045	0.26939358	3
2022-01-06-13-06-IB100p	A	Dry	13	1.01505735	2.285503	0.897862	3.42258995	0.88684311	0.98951457	0.89624058	0.03766944	3
2022-01-08-11-18-IB100p	A	Dry	11	1.01993184	2.103932	0.655959	4.33301378	0.88637259	0.98135759	0.90321062	0.00856124	7
2022-01-09-17-54-IB100p	A	Dry	17	1.05286928	2.300674	0.972621	10.1527085	0.86768031	0.98942403	0.87695496	0.40428062	0
2022-01-10-11-06-IB100p	A	Dry	11	1.01863394	1.543103	0.368894	3.00403862	0.83897266	0.98318365	0.85332242	0.03995244	4
2022-01-11-18-30-IB100p	A	Dry	18	1.0382802	2.243198	0.811046	4.59759319	0.85108133	0.98853133	0.86095534	0.24613555	0
2022-01-15-11-54-IB100p	A	Dry	11	1.01353964	2.299886	0.960166	3.02404336	0.86451189	0.98953711	0.87365282	0.08989299	3
2022-01-15-18-24-IB100p	A	Dry	18	1.04501608	2.257424	0.835492	5.57919277	0.83936409	0.9881985	0.84938814	0.29536266	0
2022-01-17-13-24-IB100p	A	Dry	13	1.02709107	2.295856	0.938709	1.85537479	0.87062661	0.98409864	0.88469446	0.12252081	4

2022-01-24-10-00-IB100p	A	Dry	10	1.00565027	2.300088	0.961221	2.79360229	0.84373584	0.98955148	0.85264472	0.26254459	4
2022-01-24-15-42-IB100p	A	Dry	15	1.01461658	2.301508	0.97399	2.68392134	0.87968691	0.99067993	0.88796279	0.10444709	5
2022-01-29-09-12-IB100p	A	Dry	09	1.00882021	2.295479	0.931942	3.33688388	0.86613432	0.99065337	0.87430613	0.26208086	4
2020-07-17-08-36-ADMINp	B	Wet	08	1.021037	1.979728	0.570063	4.75570721	0.83341387	0.98239242	0.84835128	0.32541322	6
2020-07-19-07-42-ADMINp	B	Wet	07	1.09661938	2.18527	0.775118	4.14476079	0.85565627	0.98152198	0.87176475	0.26859504	5
2020-08-27-17-30-ADMINp	B	Wet	17	1.0291702	0.779702	0.199141	2.61607445	0.87147692	0.97495615	0.89386268	0.05423554	0
2020-09-01-17-18-ADMINp	B	Wet	17	1.03524028	1.38982	0.306747	2.97525538	0.88513952	0.98040888	0.90282691	0.06818182	2
2020-09-07-07-18-ADMINp	B	Wet	07	1.03560496	0.813868	0.199159	3.90561456	0.84248536	0.96480826	0.87321533	0.01859504	3
2020-09-16-16-18-ADMINp	B	Wet	16	1.03239718	1.260959	0.28976	5.44214401	0.79193257	0.97760167	0.81007695	0.15340909	3
2020-10-20-18-42-ADMINp	B	Wet	18	1.01965627	1.770147	0.467391	6.85302578	0.74867182	0.98604425	0.75926797	0.48708678	0
2020-11-03-08-48-ADMINp	B	Dry	08	1.03406476	1.107508	0.239405	3.21920977	0.84860473	0.98119068	0.86487239	0.12396694	2
2020-11-24-16-06-ADMINp	B	Dry	16	1.031982	1.078456	0.24332	6.73322394	0.827333	0.9768421	0.8469465	0.10743802	3
2020-11-25-12-48-ADMINp	B	Dry	12	1.0395171	2.079607	0.627476	3.49770295	0.8432995	0.98380732	0.85717954	0.18181818	4
2020-12-21-10-54-ADMINp	B	Dry	10	1.04511034	1.987599	0.561251	2.46215195	0.88001084	0.98222183	0.895939	0.10123967	5
2020-12-30-13-30-ADMINp	B	Dry	13	1.03753963	2.298173	0.946376	2.0677027	0.90878529	0.98972002	0.91822462	0.20144628	3
2021-01-23-15-54-ADMINp	B	Dry	15	1.02606578	2.0872	0.636822	1.50357003	0.93357441	0.98938764	0.9435881	0.01911157	5
2021-03-05-10-42-ADMINp	B	Dry	10	1.02662615	1.859254	0.488052	3.03496523	0.86142084	0.98902452	0.87098026	0.36828512	4
2021-03-06-13-00-ADMINp	B	Dry	13	1.02594246	1.855794	0.486055	2.40447981	0.89537581	0.98909954	0.90524338	0.28099174	7
2021-03-10-07-30-ADMINp	B	Dry	07	1.01731471	1.91244	0.530632	4.78535109	0.78104362	0.98680618	0.79148635	0.48347107	6
2021-04-21-11-18-ADMINp	B	Dry	11	1.03311619	1.203395	0.263661	5.14570386	0.82647167	0.97291784	0.84947735	0.06198347	4
2021-07-21-11-36-ADMINp	B	Wet	11	1.01572159	1.905594	0.534097	6.24775625	0.78794033	0.9876637	0.797782	0.54958678	0
2021-08-05-16-00-ADMINp	B	Wet	16	1.03349709	1.714575	0.447268	4.8706422	0.84051264	0.97317265	0.86368296	0.09917355	2
2021-09-09-15-12-ADMINp	B	Wet	15	1.06381092	2.266934	0.849435	5.66178773	0.86811678	0.98659039	0.87991612	0.38429752	1
2020-10-21-09-36-ADMINp	B	Wet	09	1.1112076	2.197823	0.745387	4.3160473	0.78621355	0.97331794	0.80776643	0.4	1
2020-11-05-14-06-ADMINp	B	Dry	14	1.01968706	1.668269	0.405068	9.9537606	0.76599839	0.97127341	0.78865372	0.17777778	1
2020-11-18-18-12-ADMINp	B	Dry	18	1.03106009	0.828	0.180189	3.3436327	0.85797944	0.98808492	0.86832561	0.12444444	0
2020-12-04-18-06-ADMINp	B	Dry	18	1.03684402	1.137566	0.243991	7.1389647	0.81024469	0.96152082	0.84266994	0.01333333	0
2020-12-06-17-30-ADMINp	B	Dry	17	1.03151315	0.962824	0.203326	7.55282133	0.77647782	0.96289234	0.8064015	0.05333333	0

2020-12-09-09-42-ADMINp	B	Dry	09	1.03570001	1.170797	0.247812	7.16535464	0.75888038	0.97092745	0.78160359	0.12444444	1
2021-03-03-18-18-ADMINp	B	Dry	18	1.0377178	1.057173	0.223113	3.44569857	0.82377248	0.97955919	0.84096243	0.12444444	3
2021-03-31-12-12-ADMINp	B	Dry	12	1.02288597	0.858147	0.189093	2.27927512	0.88860533	0.98956872	0.89797233	0.06666667	6
2021-04-17-09-24-ADMINp	B	Dry	09	1.03948476	1.138677	0.245433	9.40082315	0.8208919	0.96407954	0.85147736	0.06666667	4
2021-07-02-08-06-ADMINp	B	Wet	08	1.03551586	1.870591	0.536169	8.76068091	0.84094532	0.98209274	0.85627893	0.53666667	3
2020-10-09-05-12-ADMINp	B	Wet	05	1.04655604	0.912036	0.195459	4.85518211	0.85544325	0.97631603	0.87619502	0.11851852	0
2020-10-18-06-54-ADMINp	B	Wet	06	1.02743788	1.084656	0.234883	6.26596104	0.80438934	0.96102994	0.83700757	0.20740741	3
2020-10-21-07-24-ADMINp	B	Wet	07	1.05519375	0.655739	0.159617	8.44181306	0.73894299	0.9629671	0.76736057	0.28444444	3
2020-11-06-14-30-ADMINp	B	Dry	14	1.02779957	0.927784	0.197757	4.84424053	0.81207167	0.97123899	0.83611931	0.27259259	2
2020-11-19-11-18-ADMINp	B	Dry	11	1.03935705	1.169625	0.247824	9.16828159	0.74172824	0.97049642	0.76427715	0.4345679	3
2020-11-23-15-00-ADMINp	B	Dry	15	1.03114393	0.759434	0.170459	4.65259579	0.81154134	0.97971242	0.82834649	0.45333333	2
2020-12-25-12-00-ADMINp	B	Dry	12	1.03297579	0.569777	0.144264	4.20389598	0.75554131	0.9378113	0.80564322	0.0237037	3
2021-01-13-14-42-ADMINp	B	Dry	14	1.0257781	1.221238	0.262454	3.32229784	0.84066427	0.98675761	0.85194607	0.51851852	2
2021-02-20-17-18-ADMINp	B	Dry	17	1.02644198	0.877034	0.189387	5.00100654	0.85624525	0.96940996	0.88326434	0.03111111	4
2021-02-24-08-00-ADMINp	B	Dry	08	1.03973746	0.919632	0.195377	6.76760491	0.74888587	0.95507266	0.78411403	0.17185185	5
2021-02-25-11-18-ADMINp	B	Dry	11	1.03308816	0.865845	0.192922	6.19562085	0.80984507	0.97721402	0.82872846	0.39802469	5
2021-03-06-17-12-ADMINp	B	Dry	17	1.01887512	0.583995	0.147203	2.4894408	0.80309968	0.98342473	0.81663563	0.63555556	2
2021-03-17-05-48-ADMINp	B	Dry	05	1.04233863	1.243378	0.26511	8.41600657	0.78178757	0.95269094	0.82060984	0.03703704	4
2021-03-20-09-12-ADMINp	B	Dry	09	1.0256568	1.195774	0.256362	7.12760468	0.8604278	0.97555833	0.88198498	0.12888889	4
2021-03-22-05-48-ADMINp	B	Dry	05	1.0296019	1.015813	0.213561	5.95233353	0.82021611	0.96404211	0.85080942	0.05876543	4
2021-04-08-11-30-ADMINp	B	Dry	11	1.0269179	0.784768	0.175925	5.80863286	0.79735034	0.96565452	0.82570974	0.2908642	6
2021-04-10-06-24-ADMINp	B	Dry	06	1.02530042	0.924062	0.219463	6.13602387	0.84730218	0.96273474	0.88009931	0.02962963	4
2021-04-12-10-18-ADMINp	B	Dry	10	1.01928585	0.914558	0.201825	2.94429745	0.83608518	0.98180547	0.85157927	0.31061728	5
2021-04-15-06-24-ADMINp	B	Dry	06	1.02166244	0.806258	0.178546	3.98309801	0.88885243	0.98044793	0.90657791	0.01728395	3
2021-04-20-14-54-ADMINp	B	Dry	14	1.04787446	0.903491	0.200315	4.75112522	0.7418559	0.9650413	0.76872969	0.33777778	2
2021-04-21-10-48-ADMINp	B	Dry	10	1.01915098	1.629633	0.395706	10.7815478	0.7725751	0.95993291	0.80482198	0.25037037	2
2021-05-22-13-06-ADMINp	B	Wet	13	1.02786781	1.746003	0.490137	9.23715173	0.82877748	0.98077624	0.84502198	0.72888889	0
2021-05-23-09-00-ADMINp	B	Wet	09	1.01860561	1.817748	0.528096	11.4605418	0.82204863	0.98941828	0.83084035	0.93333333	1

2021-05-23-18-18-ADMINp	B	Wet	18	1.02811535	1.721268	0.440359	8.43135543	0.87565854	0.97553537	0.89761845	0.17975309	2
2021-06-25-16-42-ADMINp	B	Wet	16	1.0266793	1.000751	0.211561	4.76869214	0.8604767	0.97724572	0.88051212	0.07901235	1
2021-06-28-10-36-ADMINp	B	Wet	10	1.03399906	0.90775	0.202588	7.18585846	0.78382098	0.96843301	0.80937036	0.33580247	3
2021-07-05-12-42-ADMINp	B	Wet	12	1.10084502	1.977433	0.560829	5.68230156	0.73556354	0.90511839	0.81267107	0.01382716	2
2021-07-10-07-24-ADMINp	B	Wet	07	1.01637051	1.801375	0.470292	10.3032148	0.83960307	0.97340888	0.86253895	0.44938272	3
2021-07-26-16-00-ADMINp	B	Wet	16	1.03455489	1.507043	0.352911	13.7960455	0.69545018	0.98923844	0.70301572	0.97777778	1
2021-08-12-08-18-ADMINp	B	Wet	08	1.02377368	0.638437	0.153881	5.05777774	0.84491387	0.97145779	0.86973812	0.13037037	2
2021-08-23-05-18-ADMINp	B	Wet	05	1.04846555	1.095622	0.233108	7.68814419	0.64169623	0.91578385	0.70070709	0.01580247	1
2021-08-24-14-30-ADMINp	B	Wet	14	1.07791965	1.368213	0.307333	7.02861131	0.74139208	0.93779677	0.79056795	0.04740741	3
2021-08-28-15-06-ADMINp	B	Wet	15	1.02367427	1.647857	0.407293	9.63570199	0.81424091	0.98388249	0.82757942	0.7308642	1
2021-09-02-06-00-ADMINp	B	Wet	06	1.03312267	1.294255	0.29002	5.13301321	0.87293323	0.97345826	0.89673412	0.02666667	2
2021-09-03-12-24-ADMINp	B	Wet	12	1.0291297	1.119752	0.237051	3.54184996	0.86507653	0.98518915	0.87808167	0.20246914	2
2021-09-04-13-06-ADMINp	B	Wet	13	1.03141679	0.701162	0.161505	4.14831539	0.82118326	0.97256858	0.84434484	0.17283951	2
2021-09-05-06-18-ADMINp	B	Wet	06	1.02760998	1.557934	0.361632	5.6396844	0.86916234	0.97729084	0.88935894	0.09777778	4
2021-09-06-13-36-ADMINp	B	Wet	13	1.02560053	1.517966	0.343949	11.4692726	0.76279306	0.98312842	0.77588344	0.80691358	1
2021-09-09-15-36-ADMINp	B	Wet	15	1.02072724	1.524654	0.362091	10.9307181	0.70954993	0.98886658	0.71753859	0.93432099	1
2021-09-10-05-30-ADMINp	B	Wet	05	1.0305533	0.937778	0.201933	8.74467325	0.75848544	0.95395359	0.7950968	0.08604938	2
2020-09-12-17-42-ADMINp	B	Wet	17	1.04816698	1.022523	0.236622	4.84852697	0.8366627	0.96107336	0.8705503	0.02716161	0
2020-10-08-07-18-ADMINp	B	Wet	07	1.04014513	0.828796	0.179192	3.2978137	0.84255173	0.94831221	0.88847504	0.00135808	4
2020-10-11-05-42-ADMINp	B	Wet	05	1.04470744	1.588297	0.380115	4.53686896	0.88416574	0.97195793	0.90967491	0.04074242	1
2020-10-14-17-00-ADMINp	B	Wet	17	1.03228189	1.421878	0.317753	5.8985985	0.86937712	0.98454012	0.88302864	0.32684473	2
2020-11-10-10-42-ADMINp	B	Dry	10	1.03158132	2.16786	0.707388	3.10524559	0.81971245	0.9891006	0.82874527	0.83657764	2
2020-11-10-11-42-ADMINp	B	Dry	11	1.03776365	1.115341	0.24545	3.24889954	0.88113097	0.96935846	0.90898363	0.02942508	1
2020-11-10-12-00-ADMINp	B	Dry	12	1.03951004	1.865106	0.490689	2.34517257	0.90642479	0.98378946	0.92136054	0.05703938	2
2020-11-26-07-18-ADMINp	B	Dry	07	1.03346784	1.552624	0.35657	3.54652338	0.89277085	0.98043342	0.91058794	0.06518787	3
2020-12-06-16-30-ADMINp	B	Dry	16	1.03545601	1.727197	0.423847	2.49034792	0.88517872	0.98782784	0.89608602	0.40742417	2
2020-12-20-18-48-ADMINp	B	Dry	18	1.03710132	2.274216	0.867971	2.18754209	0.90473072	0.98909687	0.91470385	0.25984608	2
2021-01-15-13-12-ADMINp	B	Dry	13	1.03032408	1.817403	0.465522	2.98828451	0.89792642	0.98534795	0.91127852	0.17926664	4

2021-01-18-08-00-ADMINp	B	Dry	08	1.02902455	2.056699	0.61263	2.91034939	0.92969715	0.98102295	0.94768135	0.00452694	4
2021-01-25-07-06-ADMINp	B	Dry	07	1.03059093	1.800248	0.457205	3.33588706	0.90434293	0.98671525	0.91651865	0.24717067	4
2021-03-25-13-00-ADMINp	B	Dry	13	1.02198996	2.000044	0.570376	4.96287677	0.887251	0.9824876	0.90306585	0.1466727	4
2021-04-12-11-06-ADMINp	B	Dry	11	1.02016873	1.380382	0.315212	2.05834181	0.8574517	0.98773494	0.86809899	0.63377094	4
2021-04-16-05-36-ADMINp	B	Dry	05	1.03972295	2.213043	0.768054	5.03679424	0.94676042	0.98484006	0.96133419	0.06337709	6
2021-06-28-18-18-ADMINp	B	Wet	18	1.03119829	2.279363	0.895124	3.94114999	0.91571411	0.98394654	0.93065433	0.07967406	4
2021-07-13-16-24-ADMINp	B	Wet	16	1.02175503	1.81885	0.490459	2.66781537	0.84622191	0.98202413	0.86171193	0.17926664	4
2021-07-17-12-36-ADMINp	B	Wet	12	1.02991266	2.283957	0.899253	2.72171705	0.94241389	0.98927284	0.95263294	0.09732911	4
2021-07-20-16-54-ADMINp	B	Wet	16	1.01831144	1.426573	0.323451	8.8739648	0.80546333	0.97649173	0.82485423	0.14893617	1
2021-07-24-10-42-ADMINp	B	Wet	10	1.02568908	2.238221	0.802447	6.21300684	0.85583326	0.99035295	0.86416995	0.95744681	3
2021-07-24-13-18-ADMINp	B	Wet	13	1.02644771	2.264622	0.844574	4.32326785	0.88711753	0.98891685	0.89705978	0.50701675	4
2021-07-30-09-00-ADMINp	B	Wet	09	1.02683828	2.168844	0.70884	5.51284855	0.86834108	0.9857287	0.88091285	0.42689	2
2021-08-16-10-30-ADMINp	B	Wet	10	1.03482659	2.109018	0.652279	2.51365638	0.89359478	0.98671206	0.90562872	0.21502943	5
2021-08-17-14-30-ADMINp	B	Wet	14	1.02615155	2.258736	0.8323	2.88060992	0.89434219	0.98648651	0.90659344	0.23902218	2
2021-09-07-15-06-ADMINp	B	Wet	15	1.05155772	1.059752	0.228537	3.25371366	0.83509649	0.95552391	0.87396714	0.01539158	2
2021-12-30-15-54-ADMINp	B	Dry	15	1.02641225	2.28501	0.894962	2.11363164	0.93186289	0.99018773	0.94109719	0.16659122	1
2022-01-12-11-36-ADMINp	B	Dry	11	1.02509016	2.300047	0.960449	1.70146494	0.91709412	0.98970242	0.92663623	0.2444545	2
2022-01-30-06-06-ADMINp	B	Dry	06	1.03056696	2.284979	0.897597	2.69745418	0.93884579	0.98827244	0.94998681	0.06880942	2
2020-08-16-09-42-ADMINp	B	Wet	09	1.02874823	2.287959	0.904878	2.14441433	0.90741885	0.98877987	0.91771575	0.34877127	4
2020-08-25-09-12-ADMINp	B	Wet	09	1.04192225	1.299926	0.290849	3.70774891	0.77534052	0.9868573	0.7856663	0.70699433	1
2020-08-30-14-12-ADMINp	B	Wet	14	1.02701905	2.209511	0.756431	2.66753322	0.89345866	0.98814232	0.90418014	0.55293006	3
2020-09-08-12-30-ADMINp	B	Wet	12	1.03853097	1.373836	0.305772	1.94772052	0.83331835	0.98862222	0.84290878	0.69943289	2
2020-09-19-09-30-ADMINp	B	Wet	09	1.02184298	1.650667	0.406248	5.60203699	0.78564779	0.97937708	0.80219132	0.25803403	2
2020-09-20-12-54-ADMINp	B	Wet	12	1.03575486	1.956384	0.548652	5.45668266	0.9059805	0.98063283	0.92387331	0.16068053	1
2020-11-04-06-36-ADMINp	B	Dry	06	1.05745248	1.131066	0.241007	4.62613219	0.83968578	0.96129216	0.87349697	0.00897921	5
2020-11-11-13-00-ADMINp	B	Dry	13	1.02790393	2.172568	0.720882	7.12284623	0.8333541	0.98389438	0.84699548	0.44706994	0
2020-11-21-05-24-ADMINp	B	Dry	05	1.03876737	2.282331	0.889072	3.53702346	0.93137174	0.98464597	0.94589504	0.02362949	0
2020-11-25-10-18-ADMINp	B	Dry	10	1.02276409	1.703156	0.425468	5.97755238	0.80786159	0.98782413	0.81781924	0.80434783	1

2020-11-26-07-30-ADMINp	B	Dry	07	1.03509788	2.13182	0.675067	2.97489477	0.92139024	0.98739159	0.93315585	0.25519849	4
2020-11-27-05-48-ADMINp	B	Dry	05	1.0383152	2.262368	0.846717	1.61745676	0.96373811	0.99042241	0.97305765	0.02126654	1
2020-11-27-15-30-ADMINp	B	Dry	15	1.03987336	2.219825	0.7712	2.2341529	0.9063847	0.98714405	0.91818889	0.28119093	2
2020-11-30-18-12-ADMINp	B	Dry	18	1.03879282	2.277315	0.875844	2.84489836	0.91339614	0.98251588	0.92965026	0.03780718	0
2020-12-01-12-00-ADMINp	B	Dry	12	1.03481614	1.905808	0.519094	6.15483762	0.84217538	0.98614163	0.85401058	0.55671078	0
2020-12-21-10-48-ADMINp	B	Dry	10	1.04364897	1.68876	0.408286	2.50236249	0.8728124	0.98083541	0.88986633	0.22967864	4
2021-01-12-11-54-ADMINp	B	Dry	11	1.03407165	2.181817	0.724063	2.27832021	0.89831821	0.98800804	0.90922156	0.41304348	4
2021-02-17-17-00-ADMINp	B	Dry	17	1.03171439	1.312671	0.284591	2.98427569	0.89898451	0.97957832	0.91772601	0.10586011	2
2021-02-28-08-06-ADMINp	B	Dry	08	1.03508827	2.168212	0.711293	2.07680446	0.92407543	0.98647614	0.93674382	0.09357278	6
2021-03-02-15-00-ADMINp	B	Dry	15	1.02870146	2.060142	0.612169	2.65408609	0.8819759	0.98636054	0.89417192	0.48393195	5
2021-03-03-16-24-ADMINp	B	Dry	16	1.03303749	0.815192	0.186763	2.25687312	0.82705828	0.96759059	0.85476056	0.03119093	5
2021-03-03-17-30-ADMINp	B	Dry	17	1.02978147	1.174004	0.260729	2.99363596	0.88601185	0.97986306	0.90422008	0.19659735	2
2021-03-10-13-36-ADMINp	B	Dry	13	1.01397506	1.936825	0.543158	5.57926542	0.78572342	0.99014583	0.79354313	0.93572779	3
2021-03-17-11-18-ADMINp	B	Dry	11	1.02514966	1.630597	0.384875	4.05111003	0.8441895	0.97979239	0.86160038	0.24810964	5
2021-03-27-05-30-ADMINp	B	Dry	05	1.03283988	2.272163	0.861764	2.68250984	0.92886645	0.9855697	0.94246653	0.06616257	3
2021-04-07-17-00-ADMINp	B	Dry	17	1.03266485	1.887319	0.502731	3.05586475	0.86450384	0.97887608	0.88315964	0.1758034	4
2021-05-21-14-00-ADMINp	B	Wet	14	1.02359717	1.531681	0.354066	4.82852362	0.84115219	0.97539566	0.86237024	0.08506616	0
2021-06-26-06-12-ADMINp	B	Wet	06	1.02980123	2.188559	0.741764	6.12812416	0.92799406	0.98899188	0.93832324	0.57561437	4
2021-07-04-14-12-ADMINp	B	Wet	14	1.02900816	1.99655	0.570515	7.09937543	0.85778662	0.9858572	0.87009217	0.53449906	1
2021-07-09-08-18-ADMINp	B	Wet	08	1.01962097	2.108426	0.655429	6.86657482	0.84965547	0.9829347	0.86440683	0.39697543	2
2021-07-18-14-30-ADMINp	B	Wet	14	1.03101149	1.412875	0.318438	5.07697414	0.84815317	0.96990389	0.87447136	0.04820416	2
2021-07-18-15-00-ADMINp	B	Wet	15	1.0321495	1.274093	0.274827	3.08951155	0.86470826	0.97172023	0.88987368	0.0415879	2
2021-08-09-06-06-ADMINp	B	Wet	06	1.02824248	2.111297	0.657016	3.70167445	0.93055705	0.98130649	0.94828381	0.07183365	4
2021-08-31-18-42-ADMINp	B	Wet	18	1.03304573	1.474451	0.33719	3.3367358	0.88791105	0.98458111	0.90181606	0.28355388	0
2021-09-04-09-54-ADMINp	B	Wet	09	1.03289846	2.243043	0.804773	2.03181538	0.88372894	0.98527578	0.89693563	0.26795841	1
2021-09-08-07-42-ADMINp	B	Wet	07	1.02666642	2.293101	0.92349	6.66864709	0.85100446	0.99087871	0.85883817	0.89130435	0
2021-12-01-18-24-ADMINp	B	Dry	18	1.03144915	2.244258	0.809016	3.65278086	0.93694837	0.98919839	0.94717943	0.06096408	0
2021-12-04-08-00-ADMINp	B	Dry	08	1.02758421	1.830932	0.473018	3.08155173	0.89595667	0.98604594	0.90863582	0.39697543	1

2021-12-29-06-54-ADMINp	B	Dry	06	1.02601153	2.191301	0.739015	2.8346959	0.92616001	0.98508486	0.94018298	0.08601134	4
2021-12-31-16-48-ADMINp	B	Dry	16	1.03012527	1.469496	0.334724	2.39681823	0.89083289	0.98446904	0.90488665	0.26086957	3
2022-01-05-08-12-ADMINp	B	Dry	08	1.03236072	0.800654	0.188201	2.11824313	0.90696396	0.9767581	0.92854511	0.04619565	5
2020-12-12-05-48-ADMINp	B	Dry	05	1.03877542	2.288619	0.907041	2.14310162	0.91934001	0.98972962	0.92887997	0.2403805	1
2020-12-14-11-36-ADMINp	B	Dry	11	1.03602286	1.18085	0.251632	4.7362559	0.81082305	0.97266824	0.83360699	0.07990488	1
2020-12-23-11-30-ADMINp	B	Dry	11	1.04128471	1.79175	0.456152	4.246042	0.81571325	0.98306514	0.82976521	0.33022592	2
2020-12-30-08-12-ADMINp	B	Dry	08	1.04228939	2.115364	0.659052	3.98956495	0.88677536	0.98426353	0.90095318	0.22525565	2
2021-01-06-12-06-ADMINp	B	Dry	12	1.03562763	2.1071	0.649625	3.23075263	0.86014721	0.98508353	0.87317185	0.32080856	0
2021-01-07-06-30-ADMINp	B	Dry	06	1.03868554	1.186895	0.254076	4.71861082	0.85673876	0.97000413	0.88323207	0.03766944	1
2021-01-12-13-36-ADMINp	B	Dry	13	1.03092737	1.298606	0.286257	6.36862053	0.80969253	0.96081812	0.84271155	0.03429251	2
2021-02-15-11-24-ADMINp	B	Dry	11	1.0323696	1.026362	0.222943	4.23850854	0.86488859	0.97753209	0.88476746	0.12841855	5
2021-02-15-13-18-ADMINp	B	Dry	13	1.03021086	1.446053	0.321867	2.70395261	0.8529275	0.97813809	0.87199089	0.13583829	2
2021-02-15-18-12-ADMINp	B	Dry	18	1.03751769	1.274276	0.274997	4.24370839	0.85955363	0.9661421	0.8896762	0.02321046	1
2021-02-16-06-18-ADMINp	B	Dry	06	1.04065532	1.389375	0.30579	4.35144366	0.82266287	0.95673298	0.85986674	0.00599287	4
2021-02-17-07-30-ADMINp	B	Dry	07	1.03810585	1.206438	0.259072	3.09270286	0.88800587	0.98256351	0.90376435	0.18677765	4
2021-02-18-15-24-ADMINp	B	Dry	15	1.03578014	1.675854	0.406317	4.75806518	0.83032307	0.97895969	0.8481688	0.21060642	1
2021-02-18-16-00-ADMINp	B	Dry	16	1.0281318	1.413459	0.315395	3.771705	0.8791181	0.98752477	0.89022385	0.43833532	2
2021-02-20-07-24-ADMINp	B	Dry	07	1.01976364	1.067607	0.237877	3.38805181	0.86548631	0.9695959	0.8926258	0.03086801	4
2021-02-20-16-42-ADMINp	B	Dry	16	1.03144643	2.013085	0.579322	2.06973325	0.92481235	0.99050234	0.93368013	0.27690844	1
2021-02-20-17-12-ADMINp	B	Dry	17	1.03110926	1.183867	0.25355	2.7610595	0.89809148	0.98143846	0.91507672	0.10059453	2
2021-02-20-17-36-ADMINp	B	Dry	17	1.03281511	2.097031	0.643943	1.93281289	0.93046066	0.98666266	0.94303828	0.10344828	3
2021-02-21-10-48-ADMINp	B	Dry	10	1.03358619	2.292321	0.921773	2.60208997	0.93953016	0.98430104	0.95451506	0.02511296	4
2021-02-22-18-42-ADMINp	B	Dry	18	1.03409726	2.292825	0.923802	2.75567581	0.93227172	0.98843626	0.94317839	0.12142687	0
2021-02-23-06-00-ADMINp	B	Dry	06	1.03659667	1.306221	0.28879	2.84425517	0.89127784	0.95998426	0.92842964	0.00237812	2
2021-02-23-13-36-ADMINp	B	Dry	13	1.04213683	1.235085	0.265094	4.73671917	0.79773715	0.97614097	0.8172356	0.15695601	3
2021-02-26-15-18-ADMINp	B	Dry	15	1.03129877	2.194807	0.73781	3.05591816	0.84421585	0.98803016	0.8544434	0.59491082	3
2021-02-27-08-36-ADMINp	B	Dry	08	1.03246297	2.234666	0.791434	2.79914514	0.90647533	0.98546225	0.91984785	0.20413793	4
2021-02-27-10-30-ADMINp	B	Dry	10	1.03833635	1.852726	0.483077	2.39302047	0.91128859	0.98709449	0.923203	0.19043995	5

2021-03-02-14-24-ADMINp	B	Dry	14	1.03132831	1.333083	0.291684	4.5032938	0.88152935	0.98305213	0.89672697	0.21764566	6
2021-03-03-11-00-ADMINp	B	Dry	11	1.03070995	1.384208	0.309088	4.88586222	0.84771299	0.97769223	0.86705505	0.15776457	6
2021-03-04-17-36-ADMINp	B	Dry	17	1.0288086	2.100948	0.644268	2.81025957	0.86743782	0.9888169	0.87724818	0.58863258	1
2021-03-06-07-12-ADMINp	B	Dry	07	1.02446136	1.681621	0.406609	4.11613163	0.92229369	0.98429085	0.93701337	0.15457788	6
2021-03-06-10-48-ADMINp	B	Dry	10	1.02515653	1.364524	0.299222	3.51380046	0.88831291	0.98634936	0.90060676	0.31976219	5
2021-03-12-17-00-ADMINp	B	Dry	17	1.02737691	2.295479	0.93504	2.2084706	0.93272542	0.9873473	0.94467816	0.10615933	3
2021-03-13-12-06-ADMINp	B	Dry	12	1.03004461	2.24401	0.807435	2.2619634	0.91960295	0.98944412	0.92941372	0.29450654	6
2021-03-15-14-12-ADMINp	B	Dry	14	1.02773264	1.55907	0.360509	4.03372599	0.86475135	0.97598215	0.88603193	0.09360285	4
2021-03-16-08-12-ADMINp	B	Dry	08	1.01851612	1.419079	0.317131	6.01924879	0.83934821	0.97988241	0.85658054	0.24960761	3
2021-03-16-09-30-ADMINp	B	Dry	09	1.04094969	1.476177	0.337688	7.17747164	0.85449246	0.9735032	0.87775002	0.0706302	2
2021-03-16-12-18-ADMINp	B	Dry	12	1.03827018	1.88857	0.503523	2.96507135	0.83901307	0.98844767	0.84881891	0.67120095	4
2021-03-18-10-00-ADMINp	B	Dry	10	1.03225051	1.885118	0.501624	2.85063323	0.90041699	0.98527936	0.91386973	0.22259215	5
2021-03-23-08-48-ADMINp	B	Dry	08	1.0291997	1.195315	0.263116	2.98725425	0.93747659	0.98561568	0.95115836	0.06958383	4
2021-03-24-10-54-ADMINp	B	Dry	10	1.03202468	2.057448	0.610155	2.9443965	0.86309578	0.98552955	0.87576855	0.34601665	4
2021-03-24-16-12-ADMINp	B	Dry	16	1.02844382	2.255954	0.826671	2.92841555	0.87310664	0.98935551	0.88250041	0.60879905	4
2021-03-27-14-00-ADMINp	B	Dry	14	1.02520551	0.779534	0.182226	2.4546616	0.87217368	0.9855779	0.88493631	0.33978597	6
2021-03-27-18-30-ADMINp	B	Dry	18	1.02738944	1.088321	0.230907	2.17538481	0.86761507	0.98153061	0.88394091	0.18263972	2
2021-03-28-18-54-ADMINp	B	Dry	18	1.02875524	2.243815	0.811072	1.95347922	0.88835316	0.98497377	0.9019054	0.22972652	0
2021-03-31-05-06-ADMINp	B	Dry	05	1.03490146	2.30205	0.982115	2.82292891	0.95718911	0.98971654	0.9671346	0.0313912	1
2021-04-08-14-48-ADMINp	B	Dry	14	1.02428275	0.953184	0.211116	1.66238611	0.90008062	0.98766273	0.91132387	0.32019025	6
2021-04-08-16-06-ADMINp	B	Dry	16	1.03327765	1.149127	0.245395	3.09074405	0.86951438	0.983243	0.88433315	0.2388585	3
2021-04-10-12-48-ADMINp	B	Dry	12	1.02329174	2.297468	0.946354	2.4635304	0.93659374	0.9889798	0.9470302	0.16551724	3
2021-04-12-15-00-ADMINp	B	Dry	15	1.02158992	1.409472	0.325864	2.40095751	0.90206165	0.98635775	0.914538	0.30002378	5
2021-04-15-10-12-ADMINp	B	Dry	10	1.03212987	1.460853	0.327204	3.96606405	0.85048867	0.97543545	0.87190667	0.09521998	4
2021-04-16-14-06-ADMINp	B	Dry	14	1.02604803	1.1435	0.249802	3.18563506	0.88522702	0.98475671	0.89892966	0.27814507	2
2021-04-18-12-54-ADMINp	B	Dry	12	1.02392445	2.186615	0.728878	3.12022881	0.91199333	0.98351089	0.9272834	0.15181926	5
2021-05-23-09-24-ADMINp	B	Wet	09	1.02401609	2.141828	0.702989	6.68928669	0.90865842	0.98755313	0.92011092	0.48442331	3
2021-06-26-15-00-ADMINp	B	Wet	15	1.03364026	2.263452	0.854093	4.10388576	0.92965364	0.98544511	0.9433845	0.09027348	1

2021-06-26-16-54-ADMINp	B	Wet	16	1.03390339	2.301515	0.976753	6.25300162	0.95407511	0.98855591	0.96512003	0.10939358	4
2021-06-28-13-24-ADMINp	B	Wet	13	1.02591097	1.672701	0.403137	4.98269299	0.82796615	0.97746296	0.84705629	0.16694411	0
2021-06-28-15-12-ADMINp	B	Wet	15	1.02942082	1.488173	0.350727	6.70326174	0.84755142	0.9860475	0.85954421	0.47181926	2
2021-06-29-07-30-ADMINp	B	Wet	07	1.02142706	1.909857	0.562341	7.4528104	0.87392504	0.97353813	0.89767931	0.09512485	3
2021-06-29-13-00-ADMINp	B	Wet	13	1.03067609	2.24161	0.807179	4.75736987	0.9148682	0.97746605	0.93595906	0.04261593	4
2021-07-02-09-36-ADMINp	B	Wet	09	1.02943025	1.919757	0.527904	7.37015702	0.80050481	0.95901078	0.8347193	0.02530321	2
2021-07-02-09-48-ADMINp	B	Wet	09	1.01961737	2.145547	0.699962	5.15534871	0.81067094	0.96058108	0.84393806	0.03881094	3
2021-07-03-18-24-ADMINp	B	Wet	18	1.06036326	1.656561	0.401154	7.7341711	0.78375952	0.92390776	0.84830928	0.00147444	3
2021-07-05-17-36-ADMINp	B	Wet	17	1.02949183	2.169191	0.718535	5.91108742	0.85281755	0.99029002	0.86117959	0.88589774	2
2021-07-07-12-48-ADMINp	B	Wet	12	1.03513171	2.223678	0.78316	3.94068078	0.86960526	0.98772576	0.88041165	0.52394768	2
2021-07-10-06-12-ADMINp	B	Wet	06	1.03244374	2.071592	0.625211	5.22828377	0.87396549	0.97051408	0.90051809	0.0537931	2
2021-07-11-06-06-ADMINp	B	Wet	06	1.03673703	2.209882	0.758661	3.37138933	0.9171259	0.98360297	0.93241473	0.0937931	4
2021-07-13-17-00-ADMINp	B	Wet	17	1.01352325	2.290182	0.913081	12.7730505	0.81285284	0.99148326	0.81983516	1	2
2021-07-15-05-36-ADMINp	B	Wet	05	1.02564078	1.657603	0.409488	7.13010036	0.87434826	0.98767807	0.88525632	0.56599287	3
2021-07-17-10-00-ADMINp	B	Wet	10	1.01817667	2.298385	0.949154	7.58329898	0.84908264	0.98739052	0.85992586	0.61697979	2
2021-07-17-18-30-ADMINp	B	Wet	18	1.02165979	2.19083	0.737518	8.72451288	0.80017525	0.98761869	0.81020667	0.67120095	0
2021-07-18-10-30-ADMINp	B	Wet	10	1.03162871	1.179336	0.251912	2.78611846	0.89831835	0.97951048	0.91710949	0.07976219	3
2021-07-18-12-06-ADMINp	B	Wet	12	1.03285901	2.266662	0.866252	2.49256726	0.94644986	0.98771976	0.95821699	0.06725327	3
2021-07-20-09-00-ADMINp	B	Wet	09	1.03521305	1.53626	0.36383	4.22135134	0.94645684	0.97809731	0.96765099	0.00627824	5
2021-07-21-15-00-ADMINp	B	Wet	15	1.03493651	2.255678	0.8301	2.01662662	0.92392838	0.98666646	0.9364141	0.08789536	2
2021-07-23-05-42-ADMINp	B	Wet	05	1.03257097	2.268203	0.851673	4.73645645	0.93753425	0.99022474	0.94678937	0.19690844	3
2021-07-27-18-00-ADMINp	B	Wet	18	1.04333619	1.475504	0.332756	3.35126563	0.88414914	0.97932819	0.90281189	0.13726516	2
2021-07-29-11-30-ADMINp	B	Wet	11	1.03965009	1.893987	0.507088	3.47889988	0.85541366	0.9843485	0.86901505	0.33778835	3
2021-08-04-06-54-ADMINp	B	Wet	06	1.0367191	0.956168	0.208157	3.73123753	0.89306125	0.97942111	0.91182561	0.13555291	5
2021-08-04-18-30-ADMINp	B	Wet	18	1.03385235	1.487148	0.348784	7.58534655	0.83376192	0.97363308	0.85634099	0.10915577	0
2021-08-06-08-24-ADMINp	B	Wet	08	1.03365889	1.995788	0.569269	6.0659615	0.85825732	0.97643848	0.87896712	0.15086801	3
2021-08-06-13-18-ADMINp	B	Wet	13	1.0367177	1.560385	0.366843	7.10725303	0.80198792	0.96866277	0.82793305	0.06753864	0
2021-08-07-17-24-ADMINp	B	Wet	17	1.02682997	2.001814	0.579926	4.9243585	0.89034261	0.98600204	0.90298253	0.34568371	2

2021-08-10-16-18-ADMINp	B	Wet	16	1.03131694	2.257264	0.831542	7.73546551	0.87776646	0.98796033	0.88846327	0.54435196	2
2021-08-12-09-18-ADMINp	B	Wet	09	1.02950286	2.254646	0.825827	3.12188472	0.8970854	0.97751609	0.91771931	0.0537931	3
2021-08-15-08-48-ADMINp	B	Wet	08	1.03368278	2.060724	0.616762	2.77343027	0.95605041	0.98700222	0.96864059	0.02568371	5
2021-08-17-08-48-ADMINp	B	Wet	08	1.02931991	2.091213	0.657487	8.16910325	0.7943968	0.97521031	0.81459025	0.14083234	1
2021-08-19-16-42-ADMINp	B	Wet	16	1.03271126	2.297223	0.942174	3.03638888	0.94337381	0.98876487	0.95409317	0.08347206	1
2021-08-21-11-36-ADMINp	B	Wet	11	1.04161555	1.712215	0.423777	1.60109645	0.88912277	0.98057522	0.90673591	0.09407848	3
2021-08-21-17-48-ADMINp	B	Wet	17	1.03209549	2.211381	0.759937	3.22399031	0.90481104	0.98854787	0.91529309	0.36328181	1
2021-08-21-18-00-ADMINp	B	Wet	18	1.03110639	1.889606	0.524571	5.84634292	0.8372203	0.97984186	0.85444431	0.24161712	2
2021-08-22-07-24-ADMINp	B	Wet	07	1.03006788	1.120865	0.240972	2.32573302	0.93617163	0.98009719	0.95518244	0.0179786	4
2021-08-22-15-00-ADMINp	B	Wet	15	1.02448936	2.228026	0.784275	7.39317442	0.84529673	0.98489855	0.85825766	0.42335315	1
2021-08-24-07-12-ADMINp	B	Wet	07	1.02906229	1.373343	0.30326	3.76425597	0.91865424	0.98388048	0.93370512	0.13412604	4
2021-08-25-09-54-ADMINp	B	Wet	09	1.03461859	1.867647	0.495465	4.02475165	0.90697441	0.98340489	0.92227975	0.13864447	2
2021-08-26-13-00-ADMINp	B	Wet	13	1.03884564	2.136643	0.677871	1.83265375	0.87737323	0.99048301	0.88580341	0.59686088	4
2021-08-28-10-06-ADMINp	B	Wet	10	1.03328992	1.279512	0.278202	3.87063966	0.85993483	0.96829206	0.88809447	0.02996433	1
2021-08-29-09-42-ADMINp	B	Wet	09	1.03182829	2.296954	0.941984	1.83318129	0.94248767	0.98955843	0.95243256	0.0803805	4
2021-08-29-10-30-ADMINp	B	Wet	10	1.03929011	0.964949	0.209818	2.75421262	0.88236657	0.97597786	0.90408461	0.06016647	3
2021-09-03-05-00-ADMINp	B	Wet	05	1.03266357	1.274727	0.274014	2.30252208	0.85262138	0.9582714	0.88974938	0.00171225	0
2021-09-03-07-06-ADMINp	B	Wet	07	1.03237803	2.23734	0.796692	2.78860247	0.91511981	0.98204963	0.9318468	0.08799049	4
2021-09-05-13-00-ADMINp	B	Wet	13	1.03314191	2.268035	0.858802	1.37783069	0.95524291	0.98861619	0.96624244	0.01655172	1
2021-09-05-14-00-ADMINp	B	Wet	14	1.02303902	2.127593	0.670982	10.4259523	0.78447665	0.97845941	0.80174675	0.23804994	1
2021-09-06-12-36-ADMINp	B	Wet	12	1.038959	1.239956	0.264778	3.62614494	0.85112171	0.98078549	0.86779598	0.20508918	2
2021-09-07-14-00-ADMINp	B	Wet	14	1.02778153	2.227375	0.781876	2.68316537	0.90017941	0.98391521	0.91489531	0.17407848	3
2021-09-08-11-54-ADMINp	B	Wet	11	1.02414867	2.301413	0.973088	6.80582165	0.8467306	0.9912164	0.85423385	0.9588585	2
2021-09-10-07-00-ADMINp	B	Wet	07	1.03060995	2.255973	0.829494	3.97466636	0.9328756	0.98573154	0.94637898	0.10016647	3
2021-09-10-09-42-ADMINp	B	Wet	09	1.02960454	1.929304	0.530396	3.45905553	0.8433792	0.98658108	0.85485037	0.47300832	4
2021-09-11-06-06-ADMINp	B	Wet	06	1.03642887	1.418959	0.319168	5.37839474	0.89852678	0.98382191	0.91330226	0.20485137	2
2021-09-11-08-12-ADMINp	B	Wet	08	1.0341947	2.299154	0.953388	3.04473722	0.92176899	0.99039346	0.93070989	0.32627824	4
2021-11-27-15-42-ADMINp	B	Dry	15	1.03145896	2.263341	0.840839	2.42260974	0.89484377	0.98745105	0.90621582	0.32532699	2

2021-11-29-14-24-ADMINp	B	Dry	14	1.00553038	2.296574	0.937728	6.09446465	0.93537632	0.98664611	0.94803629	0.5603805	1
2021-11-29-15-00-ADMINp	B	Dry	15	1.02901412	0.982363	0.212684	2.44548727	0.85586672	0.97771157	0.87537752	0.1293698	3
2021-12-03-17-18-ADMINp	B	Dry	17	1.03569443	2.1059	0.651165	2.84075723	0.89756015	0.98352299	0.91259702	0.17350773	2
2021-12-05-05-36-ADMINp	B	Dry	05	1.03291158	1.274649	0.276371	1.90793117	0.89178084	0.97472119	0.91490864	0.02825208	0
2021-12-05-08-12-ADMINp	B	Dry	08	1.02581491	1.171894	0.249703	1.9143636	0.90860185	0.98103213	0.92616931	0.07876338	3
2021-12-07-12-06-ADMINp	B	Dry	12	1.03032735	1.476227	0.331887	3.32762975	0.87506866	0.98097635	0.89203849	0.16908442	2
2021-12-09-16-36-ADMINp	B	Dry	16	1.02675828	1.125547	0.242151	4.62048135	0.83348538	0.97485608	0.85498301	0.10482759	1
2021-12-12-16-18-ADMINp	B	Dry	16	1.02686147	1.595474	0.374774	1.80185035	0.91050192	0.98426282	0.92505975	0.11985731	1
2021-12-13-07-18-ADMINp	B	Dry	07	1.02527917	2.295468	0.932304	3.25710529	0.92324393	0.98829158	0.93418172	0.27995244	2
2021-12-13-13-42-ADMINp	B	Dry	13	1.02728362	1.714289	0.422401	4.70681526	0.8756178	0.98732694	0.88685699	0.4463258	1
2021-12-30-05-18-ADMINp	B	Dry	05	1.03596901	2.302218	0.986748	3.90739305	0.97585813	0.99090605	0.98481399	0.01360285	0
2021-12-30-13-00-ADMINp	B	Dry	13	1.03344491	2.27467	0.868575	1.71008389	0.93643249	0.98806527	0.94774355	0.09160523	2
2022-01-03-07-30-ADMINp	B	Dry	07	1.02107527	2.286038	0.900568	2.33140805	0.92074495	0.98779994	0.93211683	0.27434007	4
2022-01-03-11-06-ADMINp	B	Dry	11	1.02279791	2.152257	0.692188	2.14650752	0.90586285	0.98812068	0.91675326	0.36470868	2
2022-01-03-16-24-ADMINp	B	Dry	16	1.02529416	1.858823	0.497623	2.13878218	0.8999555	0.9816979	0.91673365	0.14449465	2
2022-01-06-09-18-ADMINp	B	Dry	09	1.02187427	2.268546	0.852377	3.3222997	0.87009895	0.98791829	0.88073979	0.49959572	3
2022-01-06-14-36-ADMINp	B	Dry	14	1.02793631	2.301672	0.976326	1.7870071	0.92030571	0.9902065	0.92940786	0.33883472	3
2022-01-09-11-54-ADMINp	B	Dry	11	1.03364436	2.292662	0.921806	1.73039358	0.95363632	0.98989662	0.96336962	0.04494649	2
2022-01-10-05-00-ADMINp	B	Dry	05	1.02903841	2.239846	0.809701	3.04007337	0.92830584	0.98635601	0.94114684	0.08580262	0
2022-01-11-06-42-ADMINp	B	Dry	06	1.01981345	1.784423	0.473819	3.18601506	0.91925357	0.97514903	0.94268008	0.03995244	4
2022-01-14-05-36-ADMINp	B	Dry	05	1.03283902	2.243641	0.811899	2.88064927	0.94574881	0.98845255	0.95679738	0.0591201	0
2022-01-15-05-00-ADMINp	B	Dry	05	1.03761326	2.300309	0.965199	3.36762062	0.97468295	0.98999427	0.98453393	0.00646849	0
2022-01-18-17-18-ADMINp	B	Dry	17	1.02617675	2.161455	0.709221	2.17094336	0.91218393	0.98644528	0.92471822	0.20375743	1
2022-01-19-06-30-ADMINp	B	Dry	06	1.03419648	2.293372	0.924807	2.05986233	0.93971722	0.98680709	0.95228057	0.07196195	3
2022-01-20-18-00-ADMINp	B	Dry	18	1.03404019	1.575127	0.367551	2.57993694	0.92959969	0.98409941	0.94461969	0.04718193	0
2022-01-22-08-24-ADMINp	B	Dry	08	1.02689763	2.276465	0.872019	2.35361493	0.93613894	0.98878035	0.94676127	0.15714625	3
2022-01-23-06-12-ADMINp	B	Dry	06	1.03216669	2.298942	0.952728	2.13843205	0.92377163	0.98919363	0.9338633	0.20142687	1
2022-01-26-14-36-ADMINp	B	Dry	14	1.02939558	2.294074	0.925851	2.12384	0.88844894	0.98963972	0.89774988	0.5046849	1

2022-01-26-15-36-ADMINp	B	Dry	15	1.01933024	1.684876	0.438795	6.40146882	0.84511452	0.98839052	0.8550411	0.72722949	0
2022-01-27-12-00-ADMINp	B	Dry	12	1.02883029	2.212516	0.759878	2.7765593	0.84808806	0.98985846	0.8567771	0.77117717	2
2022-01-28-11-12-ADMINp	B	Dry	11	1.0307518	1.968501	0.54927	2.46324563	0.84770678	0.98931588	0.8568616	0.66617123	2
2020-12-18-12-24-CSLIBp	C	Dry	12	1.04237493	1.53917	0.350917	14.8161972	0.83556237	0.96677502	0.864278	0.22479167	3
2020-12-19-15-06-CSLIBp	C	Dry	15	1.01989793	1.794003	0.498793	9.26592471	0.88964774	0.9703617	0.91682075	0.19333333	1
2020-12-23-12-24-CSLIBp	C	Dry	12	1.0104376	0.37212	0.127308	12.1310594	0.69721843	0.96859255	0.71982634	0.22493056	4
2020-12-27-05-18-CSLIBp	C	Dry	05	1.01704273	1.92039	0.532144	7.81081629	0.89637026	0.98751046	0.90770711	0.18368056	1
2020-12-27-15-06-CSLIBp	C	Dry	15	1.02403037	1.575613	0.407195	14.9732809	0.8646199	0.94944622	0.91065706	0.01319444	3
2020-12-27-16-06-CSLIBp	C	Dry	16	1.01861925	1.495331	0.405607	7.56014191	0.85243441	0.95780606	0.88998645	0.01833333	4
2020-12-30-05-48-CSLIBp	C	Dry	05	1.01979256	1.679684	0.406005	18.7969129	0.82598859	0.98496165	0.83859975	0.6	1
2021-01-07-06-54-CSLIBp	C	Dry	06	1.08092062	1.566616	0.363185	16.5906718	0.82016136	0.95210461	0.86141938	0.12375	5
2021-01-13-05-42-CSLIBp	C	Dry	05	1.00510087	0.582791	0.15787	10.2690679	0.80550602	0.96542173	0.83435663	0.07291667	1
2021-01-14-16-48-CSLIBp	C	Dry	16	1.01682324	0.909467	0.209297	11.7765653	0.80331714	0.97543082	0.82355111	0.22819444	3
2021-01-16-06-54-CSLIBp	C	Dry	06	1.07820373	1.744426	0.440224	14.6931591	0.84809776	0.94516014	0.89730589	0.06354167	6
2021-01-18-15-30-CSLIBp	C	Dry	15	0.99596296	1.216715	0.282041	9.56560998	0.85638441	0.98254969	0.871594	0.12638889	2
2021-01-20-14-54-CSLIBp	C	Dry	14	1.00052743	0.822029	0.185281	11.4177875	0.81213759	0.98372861	0.82557078	0.048125	2
2021-01-26-13-30-CSLIBp	C	Dry	13	1.00532657	0.697879	0.160732	10.5687452	0.76280822	0.98420374	0.77505112	0.39722222	6
2021-01-28-09-18-CSLIBp	C	Dry	09	1.06810609	2.027305	0.594213	15.5770408	0.82856888	0.91442009	0.90611404	0.021875	5
2021-01-28-14-30-CSLIBp	C	Dry	14	0.99787981	0.691167	0.175124	5.20552438	0.83436229	0.96653614	0.86324997	0.19791667	2
2021-01-30-07-00-CSLIBp	C	Dry	07	1.01460124	1.527482	0.381886	6.2069178	0.8067589	0.95069349	0.84860042	0.09722222	7
2021-01-31-07-42-CSLIBp	C	Dry	07	0.99689587	1.558348	0.389273	18.3546604	0.84247267	0.95082309	0.88604565	0.06270833	4
2021-02-02-08-54-CSLIBp	C	Dry	08	1.00430462	0.860294	0.208238	12.8839023	0.7945221	0.96341626	0.82469244	0.14423611	7
2021-02-02-13-30-CSLIBp	C	Dry	13	1.00708388	0.829031	0.19061	14.2029602	0.78980684	0.97767522	0.80784173	0.17472222	3
2021-02-03-10-00-CSLIBp	C	Dry	10	1.0657296	1.236174	0.28581	14.2351634	0.78394265	0.92697592	0.84569904	0.04277778	3
2021-02-05-17-54-CSLIBp	C	Dry	17	1.00534438	1.492288	0.357758	11.3081755	0.83759736	0.98412749	0.85110656	0.13590278	1
2021-02-18-11-54-CSLIBp	C	Dry	11	1.00258618	0.642757	0.167226	5.77807179	0.80199062	0.97672815	0.82109912	0.15347222	3
2021-02-20-09-30-CSLIBp	C	Dry	09	1.00537007	0.447445	0.138728	14.4206618	0.75379428	0.96488292	0.78122876	0.06138889	7
2021-02-25-10-54-CSLIBp	C	Dry	10	1.00161085	0.493121	0.143222	13.9252764	0.77362601	0.97368002	0.79453825	0.16041667	4

2021-02-25-18-42-CSLIBp	C	Dry	18	1.00073332	1.665678	0.433587	12.0802565	0.79120877	0.98841339	0.80048367	0.7375	1
2021-03-01-06-36-CSLIBp	C	Dry	06	1.0120457	1.466178	0.339469	12.5422383	0.83596198	0.97130889	0.86065513	0.24083333	5
2021-03-02-10-00-CSLIBp	C	Dry	10	0.99395903	0.901302	0.210307	18.166129	0.71260004	0.946373	0.7529801	0.08145833	2
2021-03-03-08-36-CSLIBp	C	Dry	08	1.03873791	1.504908	0.353908	18.7871664	0.85464412	0.96370906	0.88682795	0.11333333	4
2021-03-03-15-48-CSLIBp	C	Dry	15	1.01116264	0.630538	0.15812	16.4639792	0.67823751	0.93167125	0.72797944	0.04	5
2021-03-05-18-48-CSLIBp	C	Dry	18	1.00261585	1.388911	0.310962	11.0664679	0.79586054	0.98378553	0.80897768	0.36111111	1
2021-03-06-09-06-CSLIBp	C	Dry	09	1.00040252	1.134477	0.241964	12.732527	0.8208778	0.98322688	0.83488136	0.3	6
2021-03-07-16-48-CSLIBp	C	Dry	16	1.00468166	0.132756	0.10851	11.6671429	0.70052521	0.96193235	0.72824789	0.10416667	6
2021-03-16-14-24-CSLIBp	C	Dry	14	1.00406575	0.447462	0.130977	10.6386921	0.73436856	0.97019617	0.75692791	0.17888889	6
2021-03-20-13-30-CSLIBp	C	Dry	13	1.00042129	0.780916	0.185635	14.7283113	0.81828151	0.98235709	0.83297766	0.01875	7
2021-03-22-14-24-CSLIBp	C	Dry	14	1.02408694	1.418441	0.323072	5.95595784	0.63464377	0.87829184	0.72258871	0.00527778	4
2021-03-23-06-00-CSLIBp	C	Dry	06	0.9891453	1.368105	0.319096	12.6189167	0.82597884	0.9662258	0.85485074	0.16958333	5
2021-03-28-17-30-CSLIBp	C	Dry	17	1.01932765	1.214307	0.279209	15.8940798	0.80150486	0.97490364	0.82213752	0.19583333	1
2021-04-02-14-06-CSLIBp	C	Dry	14	1.00475608	0.25274	0.118675	13.2989859	0.71025369	0.94108425	0.7547185	0.00666667	4
2021-04-07-08-24-CSLIBp	C	Dry	08	1.00751665	0.624099	0.158296	9.73142588	0.77208873	0.96696076	0.79846955	0.08611111	3
2021-04-15-13-00-CSLIBp	C	Dry	13	0.99749658	0.625388	0.152612	12.6207854	0.79073974	0.98341787	0.80407298	0.19055556	4
2021-04-22-17-18-CSLIBp	C	Dry	17	1.05301586	1.737294	0.491487	17.283276	0.77206125	0.98034811	0.78753786	0.53083333	1
2021-04-26-17-18-CSLIBp	C	Dry	17	1.05559883	1.777443	0.491578	18.7802412	0.79943203	0.97425817	0.8205546	0.36020833	0
2021-04-29-08-00-CSLIBp	C	Dry	08	0.98589574	1.586351	0.381348	13.5125161	0.8592409	0.98390259	0.87329875	0.14875	5
2021-05-01-12-48-CSLIBp	C	Wet	12	0.9466449	1.717465	0.47769	10.9961573	0.81910977	0.98083168	0.83511757	0.55409722	4
2021-05-02-11-18-CSLIBp	C	Wet	11	0.9780161	1.960159	0.544633	12.9034277	0.87891387	0.9616952	0.91392145	0.091875	5
2021-05-08-09-06-CSLIBp	C	Wet	09	0.97347226	1.790105	0.51521	16.4878647	0.79817656	0.98184149	0.81293831	0.64777778	5
2021-05-11-08-24-CSLIBp	C	Wet	08	1.02829199	2.039141	0.619886	17.2425087	0.86189847	0.97437565	0.88456487	0.47118056	2
2021-05-12-09-54-CSLIBp	C	Wet	09	0.9884864	1.931411	0.566573	19.6238603	0.81205352	0.97840213	0.82997931	0.55222222	3
2021-05-16-13-18-CSLIBp	C	Wet	13	0.98275187	1.559615	0.371231	8.77770301	0.82506433	0.97901531	0.84274916	0.5	3
2021-05-17-16-54-CSLIBp	C	Wet	16	1.06019193	0.953008	0.209118	15.0035108	0.70271235	0.9564142	0.73473643	0.14694444	1
2021-05-21-07-18-CSLIBp	C	Wet	07	0.99323067	1.985866	0.61575	20.0580785	0.81114076	0.98315509	0.82503846	0.76527778	3
2021-05-22-15-42-CSLIBp	C	Wet	15	1.00241213	1.109523	0.244198	10.592636	0.8461402	0.98459908	0.85937537	0.02944444	6

2021-05-30-06-24-CSLIBp	C	Wet	06	0.99589821	2.027371	0.65404	20.8273871	0.8024116	0.98491214	0.81470373	0.869375	4
2021-06-02-11-48-CSLIBp	C	Wet	11	1.00578775	1.247151	0.320419	4.65384421	0.81130978	0.94928162	0.85465657	0.10625	1
2021-06-12-07-36-CSLIBp	C	Wet	07	1.00103652	1.845197	0.536047	15.7176054	0.82831595	0.98595123	0.84011858	0.87888889	1
2021-06-16-12-24-CSLIBp	C	Wet	12	0.97386855	2.122394	0.695383	12.8697975	0.81981622	0.98427189	0.83291642	0.75833333	2
2021-06-18-11-24-CSLIBp	C	Wet	11	0.97815211	1.928315	0.565763	12.2333402	0.81843033	0.97818595	0.83668175	0.46666667	4
2021-06-20-07-48-CSLIBp	C	Wet	07	0.97184318	1.836212	0.51881	19.8180974	0.82799716	0.98267197	0.84259772	0.64625	3
2021-06-23-12-06-CSLIBp	C	Wet	12	0.97987659	1.91148	0.550131	11.430856	0.84427129	0.98138622	0.86028444	0.61625	2
2021-06-25-18-00-CSLIBp	C	Wet	18	1.04013023	1.619795	0.388036	16.4296327	0.70739023	0.97252018	0.72737846	0.35701389	1
2021-06-25-18-24-CSLIBp	C	Wet	18	1.03871749	1.68514	0.415518	18.3988734	0.72719303	0.97401614	0.74659238	0.36555556	2
2021-07-04-10-12-CSLIBp	C	Wet	10	0.97115957	1.716949	0.456073	16.2737986	0.80877097	0.97799811	0.82696578	0.503125	3
2021-07-09-05-48-CSLIBp	C	Wet	05	0.95579356	1.730164	0.467001	15.2801975	0.81515489	0.98379426	0.82858268	0.75048611	5
2021-07-10-11-36-CSLIBp	C	Wet	11	0.97337988	1.695158	0.456011	9.51269197	0.83118081	0.96090964	0.86499372	0.15166667	5
2021-07-13-16-42-CSLIBp	C	Wet	16	1.05117877	1.115634	0.259984	16.096704	0.77069546	0.970316	0.79427265	0.23826389	1
2021-07-20-18-12-CSLIBp	C	Wet	18	1.0219625	1.136625	0.241442	10.1741101	0.83610947	0.97939472	0.85370021	0.045625	1
2021-07-22-05-36-CSLIBp	C	Wet	05	1.00596307	1.049944	0.23087	17.5257556	0.83960363	0.98329941	0.85386366	0.16166667	6
2021-09-02-10-54-CSLIBp	C	Wet	10	0.99156766	1.997168	0.608217	16.7873791	0.80353416	0.98858534	0.81281213	0.97513889	1
2021-09-05-17-30-CSLIBp	C	Wet	17	1.00174794	0.291104	0.121095	8.2068128	0.77444431	0.98123062	0.7892582	0.08987847	0
2020-12-22-14-00-CSLIBp	C	Dry	14	1.00470809	0.357011	0.122017	5.55857247	0.70465777	0.96057196	0.73358145	0.09234948	1
2021-01-26-14-36-CSLIBp	C	Dry	14	1.00413656	1.267608	0.334567	10.1605068	0.81705142	0.96005418	0.8510472	0.10140335	4
2021-02-10-10-42-CSLIBp	C	Dry	10	1.00836439	0.848034	0.202555	7.57430041	0.72492687	0.94868869	0.76413567	0.05387053	5
2021-02-14-10-54-CSLIBp	C	Dry	10	1.00543977	0.424034	0.136764	11.9114292	0.79784559	0.96509527	0.82670138	0.02987777	5
2021-03-03-07-00-CSLIBp	C	Dry	07	0.99648137	1.479685	0.344519	15.1038141	0.8051883	0.97871578	0.82269881	0.34857402	5
2021-03-11-12-00-CSLIBp	C	Dry	12	1.01479824	1.04525	0.239576	9.31848805	0.67409284	0.93484335	0.72107572	0.03802626	6
2021-03-11-13-24-CSLIBp	C	Dry	13	1.0270166	0.777108	0.17278	9.53432502	0.61058024	0.92059381	0.66324609	0.01448619	2
2021-03-12-17-00-CSLIBp	C	Dry	17	1.01273006	0.830457	0.205846	10.5558676	0.65007559	0.92322129	0.70413842	0.00995926	4
2021-03-14-13-00-CSLIBp	C	Dry	13	1.00458906	1.097521	0.258594	11.3547286	0.82324517	0.97526804	0.84412197	0.07243096	5
2021-03-31-16-48-CSLIBp	C	Dry	16	1.03425675	0.707063	0.161907	10.7176633	0.59021869	0.9328849	0.63268115	0.0421005	3
2021-04-10-09-30-CSLIBp	C	Dry	09	1.03863754	1.257668	0.282737	15.0783562	0.75169147	0.94137159	0.79850664	0.03621548	7

2021-04-16-15-36-CSLIBp	C	Dry	15	1.02030377	0.831578	0.205846	8.151222	0.77481779	0.9686498	0.79989465	0.19782707	5
2021-05-06-08-00-CSLIBp	C	Wet	08	0.98065378	1.756982	0.456113	12.6000851	0.82601535	0.96105016	0.85949245	0.1955636	5
2021-05-23-05-30-CSLIBp	C	Wet	05	0.99939532	2.069613	0.669207	20.2083959	0.80586776	0.98518428	0.81798683	0.9578995	4
2021-05-25-11-06-CSLIBp	C	Wet	11	0.98717206	2.10682	0.689761	18.296439	0.81542608	0.98112768	0.83111108	0.6364871	4
2021-06-04-09-06-CSLIBp	C	Wet	09	0.99389234	2.149198	0.740846	17.8687749	0.81199432	0.98320861	0.82586168	0.79447714	4
2021-06-23-16-36-CSLIBp	C	Wet	16	0.98719496	2.128048	0.75166	21.7555628	0.83800416	0.98953747	0.84686451	0.80851064	0
2021-07-09-08-00-CSLIBp	C	Wet	08	0.99938397	2.102578	0.652728	10.8528361	0.84763956	0.97134287	0.87264712	0.36939792	2
2021-07-13-06-48-CSLIBp	C	Wet	06	0.97866877	2.003138	0.602133	10.6962106	0.82735925	0.97721568	0.84664959	0.49298325	3
2021-07-15-05-06-CSLIBp	C	Wet	05	0.96595366	1.739799	0.434225	6.25907338	0.8073944	0.97450287	0.82851926	0.40697148	5
2021-07-23-18-48-CSLIBp	C	Wet	18	1.00522402	1.012215	0.221197	6.51513218	0.84371539	0.95576675	0.88276286	0.16296967	0
2021-08-07-06-06-CSLIBp	C	Wet	06	0.99972136	2.080137	0.633412	6.46721448	0.85880656	0.96275365	0.89203148	0.12901766	5
2021-08-09-14-54-CSLIBp	C	Wet	14	1.00045972	0.148747	0.10768	7.38295643	0.79999411	0.98002013	0.81630376	0.01674966	1
2021-08-29-17-18-CSLIBp	C	Wet	17	1.05271106	1.393401	0.326658	12.150791	0.73171021	0.97843149	0.74784001	0.40018108	0
2021-09-19-10-24-CSLIBp	C	Wet	10	0.98706188	2.027188	0.588963	10.850565	0.83006406	0.95239023	0.87155878	0.08329561	2
2021-09-21-11-18-CSLIBp	C	Wet	11	0.98703148	2.015019	0.595765	15.8042962	0.81331101	0.95695355	0.84989602	0.22815754	0
2021-10-15-07-12-CSLIBp	C	Wet	07	1.00324203	1.619728	0.434806	14.4440509	0.85032811	0.95532487	0.89009314	0.08963332	4
2021-11-17-16-00-CSLIBp	C	Dry	16	1.007595	0.316895	0.122785	7.91169724	0.67221819	0.95588401	0.70324242	0.04708013	0
2021-11-18-12-06-CSLIBp	C	Dry	12	1.01893985	0.525798	0.143963	11.7043165	0.62465274	0.94337947	0.66214366	0.05432322	0
2021-12-03-12-18-CSLIBp	C	Dry	12	1.00755582	0.394876	0.144419	3.68170056	0.74937868	0.97166546	0.77123116	0.08239022	0
2021-12-07-07-48-CSLIBp	C	Dry	07	1.00855284	0.494181	0.147678	14.3046828	0.7738116	0.97189482	0.79618862	0.15889543	3
2021-12-18-05-30-CSLIBp	C	Dry	05	1.02275162	0.945079	0.218724	15.0779778	0.66965568	0.95471733	0.70141775	0.12222725	5
2022-01-08-15-36-CSLIBp	C	Dry	15	1.00875186	1.028107	0.231159	12.5245928	0.78343184	0.96382702	0.81283449	0.09506564	1
2022-01-18-17-42-CSLIBp	C	Dry	17	1.00634365	1.433158	0.335548	10.5768476	0.79431974	0.9736226	0.81583947	0.12222725	1
2022-01-28-13-00-CSLIBp	C	Dry	13	1.03711432	1.131898	0.273317	14.445949	0.70429975	0.97354106	0.72344124	0.45948393	2
2022-01-31-18-06-CSLIBp	C	Dry	18	1.02743842	1.786018	0.472422	17.0212374	0.87264943	0.97968065	0.89074887	0.06518787	0
2021-01-04-18-00-CSLIBp	C	Dry	18	1.01244069	1.064321	0.22978	11.340546	0.82908961	0.98175118	0.84450075	0.32132964	1
2021-02-05-05-18-CSLIBp	C	Dry	05	1.00619811	1.295319	0.283907	15.0601026	0.82775476	0.98169132	0.84319251	0.19390582	0
2021-02-16-13-06-CSLIBp	C	Dry	13	1.00579596	0.40048	0.127853	10.5500826	0.72355437	0.978953	0.73911043	0.27423823	3

2021-03-01-10-12-CSLIBp	C	Dry	10	1.00578725	0.890464	0.216307	9.95673916	0.81164294	0.97224405	0.83481399	0.15512465	6
2021-03-07-14-36-CSLIBp	C	Dry	14	1.02591692	0.330387	0.120039	6.84438047	0.60739672	0.94874587	0.64021014	0.03739612	2
2021-03-18-12-24-CSLIBp	C	Dry	12	0.99917269	0.62638	0.159688	13.4021811	0.76949217	0.97858923	0.78632806	0.13088643	4
2021-03-22-08-42-CSLIBp	C	Dry	08	1.00822085	1.329203	0.329323	8.60583536	0.85984062	0.97917542	0.87812726	0.03185596	6
2021-03-26-07-36-CSLIBp	C	Dry	07	0.99269355	0.715662	0.18181	14.167901	0.81521156	0.97200891	0.83868734	0.06232687	3
2021-03-27-06-36-CSLIBp	C	Dry	06	1.00111316	0.83228	0.183379	11.4199927	0.80161518	0.98095945	0.81717464	0.05609418	3
2021-03-27-17-12-CSLIBp	C	Dry	17	1.02713605	1.037312	0.225876	9.21808992	0.78679975	0.97436834	0.80749725	0.29432133	2
2021-04-01-15-00-CSLIBp	C	Dry	15	1.04678537	0.42884	0.129803	8.36816046	0.51034589	0.90654482	0.56295715	0.01800554	2
2021-04-04-15-06-CSLIBp	C	Dry	15	1.00238758	1.564095	0.373092	17.7160168	0.82266232	0.95618652	0.86035757	0.03047091	4
2021-05-04-08-06-CSLIBp	C	Wet	08	0.95647269	1.662635	0.409459	10.8125316	0.82138323	0.9793025	0.83874312	0.48199446	3
2021-06-02-16-18-CSLIBp	C	Wet	16	1.01866464	1.142185	0.272392	14.4001605	0.71465005	0.97766991	0.73097273	0.4432133	3
2021-06-04-05-06-CSLIBp	C	Wet	05	1.00963131	2.038257	0.657041	19.4064395	0.80996644	0.98583026	0.82160842	0.92105263	0
2021-06-06-11-24-CSLIBp	C	Wet	11	0.99404186	1.648092	0.43936	17.8738176	0.80899913	0.98481954	0.82146942	0.77700831	3
2021-06-25-14-42-CSLIBp	C	Wet	14	1.00466314	1.570264	0.410703	6.71983496	0.86255172	0.97129598	0.8880421	0.14542936	3
2021-07-15-10-00-CSLIBp	C	Wet	10	0.99515711	2.252604	0.828078	6.70377406	0.86751292	0.98516605	0.88057533	0.82409972	0
2021-07-21-13-06-CSLIBp	C	Wet	13	1.00043697	2.187373	0.758294	7.19670141	0.89229108	0.98713512	0.9039199	0.79432133	2
2021-08-01-06-48-CSLIBp	C	Wet	06	0.99880339	0.871277	0.206672	16.3079499	0.817846	0.96877895	0.84420291	0.06301939	4
2021-08-13-11-00-CSLIBp	C	Wet	11	0.99081587	2.087854	0.691795	24.6876832	0.80071242	0.98619643	0.81191981	0.89750693	0
2021-08-15-15-36-CSLIBp	C	Wet	15	1.01296927	0.966388	0.214193	6.66564754	0.78626101	0.98000219	0.80230536	0.13850416	0
2021-08-29-06-36-CSLIBp	C	Wet	06	1.0035274	0.35069	0.144001	13.1866433	0.75795473	0.97683789	0.77592684	0.06578947	3
2021-08-31-09-00-CSLIBp	C	Wet	09	0.99360089	1.085249	0.22912	12.5002794	0.72456227	0.96150433	0.75357151	0.25138504	2
2021-09-01-09-00-CSLIBp	C	Wet	09	0.99407544	1.979934	0.590551	23.4259929	0.79427419	0.98780528	0.80407971	0.97368421	0
2021-09-12-18-00-CSLIBp	C	Wet	18	1.00868195	1.053583	0.244081	16.0140668	0.72677073	0.97506209	0.74535841	0.34903047	0
2021-09-16-16-54-CSLIBp	C	Wet	16	1.00801728	1.859894	0.527489	6.28239148	0.8806137	0.98398372	0.89494743	0.3767313	0
2021-10-04-08-42-CSLIBp	C	Wet	08	0.99936049	2.073074	0.650906	8.54415348	0.84112544	0.95051114	0.88491907	0.06232687	0
2021-10-09-17-12-CSLIBp	C	Wet	17	1.01016678	1.284083	0.288706	9.44667991	0.83096925	0.98034614	0.84762842	0.01800554	1
2021-10-17-12-18-CSLIBp	C	Wet	12	1.00630641	1.095985	0.24599	10.7822851	0.81631263	0.98210948	0.83118293	0.08310249	0
2021-10-19-18-00-CSLIBp	C	Wet	18	1.01581694	1.040875	0.238353	14.1541832	0.79745486	0.98318337	0.81109474	0.27908587	1

2021-11-22-11-42-CSLIBp	C	Dry	11	1.00372772	0.028177	0.101019	6.65322899	0.68824276	0.96395863	0.71397542	0.09972299	0
2021-11-24-07-24-CSLIBp	C	Dry	07	1.00851235	0.390848	0.143598	9.07082715	0.72022517	0.95944164	0.75067116	0.11166898	2
2020-12-20-15-06-CSLIBp	C	Dry	15	1.02209415	0.62727	0.154856	10.1940951	0.62089913	0.95358842	0.65111857	0.10749108	3
2020-12-20-17-42-CSLIBp	C	Dry	17	1.00826401	0.631923	0.166126	11.70158	0.73846846	0.97807586	0.75502166	0.3353151	1
2020-12-22-12-48-CSLIBp	C	Dry	12	1.01556539	0.382024	0.134622	10.8935121	0.66920801	0.96518649	0.69334581	0.17065398	3
2020-12-23-11-12-CSLIBp	C	Dry	11	1.04202466	1.049801	0.264014	20.7084231	0.73552477	0.96315757	0.76365985	0.18178359	3
2020-12-27-09-18-CSLIBp	C	Dry	09	1.01098391	2.286727	0.905343	7.22267807	0.88398163	0.98697383	0.8956485	0.91224733	3
2021-02-01-12-12-CSLIBp	C	Dry	12	1.00669123	0.222296	0.111502	7.2473251	0.68930016	0.96331601	0.71554937	0.11605232	4
2021-02-03-08-18-CSLIBp	C	Dry	08	1.10334275	1.780429	0.455412	11.4103255	0.80275237	0.92769034	0.86532363	0.03467301	5
2021-02-06-14-00-CSLIBp	C	Dry	14	0.99988159	0.435748	0.134938	11.9848711	0.71868971	0.96171013	0.74730388	0.08323425	3
2021-02-06-15-54-CSLIBp	C	Dry	15	1.00429389	0.462452	0.140255	13.7796769	0.68658418	0.96080082	0.71459575	0.12085612	2
2021-02-07-12-00-CSLIBp	C	Dry	12	1.02310404	0.546693	0.144641	11.1434449	0.62222592	0.92547749	0.67232961	0.02739596	6
2021-02-10-17-06-CSLIBp	C	Dry	17	1.02429099	0.801806	0.197798	11.0548131	0.7890998	0.97769665	0.80710085	0.19476813	2
2021-02-12-14-00-CSLIBp	C	Dry	14	1.00364794	0.881229	0.194546	9.89383396	0.80541001	0.98087661	0.82111246	0.05041617	2
2021-02-13-07-06-CSLIBp	C	Dry	07	1.00431091	0.862934	0.202123	14.8741934	0.83604776	0.96818756	0.86351838	0.10758621	3
2021-02-13-14-36-CSLIBp	C	Dry	14	1.00402948	0.617876	0.154365	10.8391239	0.7438339	0.97901331	0.75977915	0.22606421	3
2021-02-21-06-30-CSLIBp	C	Dry	06	0.99686576	1.808582	0.471854	9.33604203	0.88114794	0.96706542	0.9111565	0.05231867	5
2021-02-22-05-06-CSLIBp	C	Dry	05	1.01131484	1.248934	0.280263	6.47152519	0.81198179	0.96515728	0.84129479	0.15410226	3
2021-02-23-05-42-CSLIBp	C	Dry	05	0.99662072	1.798072	0.466859	12.3747264	0.86026194	0.97359018	0.88359759	0.29307967	6
2021-02-23-13-42-CSLIBp	C	Dry	13	1.00345561	0.664694	0.175324	14.4476173	0.69081579	0.96051826	0.71921151	0.14302021	4
2021-03-02-09-48-CSLIBp	C	Dry	09	1.01001431	0.692322	0.165611	14.2258732	0.75227406	0.96903405	0.77631333	0.19120095	3
2021-03-03-06-42-CSLIBp	C	Dry	06	0.99815098	1.842928	0.5052	11.9047162	0.76094351	0.97346396	0.78168638	0.38820452	5
2021-03-03-13-36-CSLIBp	C	Dry	13	1.02441713	1.542496	0.356861	6.29831791	0.73810444	0.93429492	0.79001226	0.07191439	3
2021-03-03-17-30-CSLIBp	C	Dry	17	1.01312887	1.614618	0.383367	12.8957097	0.67455624	0.93075093	0.7247441	0.05707491	1
2021-03-14-14-06-CSLIBp	C	Dry	14	1.00169939	0.322465	0.123165	13.4696037	0.70320414	0.95994945	0.73254288	0.08523187	3
2021-03-20-06-54-CSLIBp	C	Dry	06	0.99841387	0.954218	0.227104	11.2934644	0.82069502	0.97611521	0.8407768	0.11586207	6
2021-03-22-11-00-CSLIBp	C	Dry	11	1.0051296	1.041564	0.227294	11.5380287	0.71668106	0.93371031	0.76756254	0.03976219	3
2021-03-22-15-36-CSLIBp	C	Dry	15	1.01025446	0.494021	0.137264	9.61716541	0.66432252	0.94201358	0.70521543	0.05850178	3

2021-03-24-05-00-CSLIBp	C	Dry	05	1.02115128	1.592342	0.376277	14.1522384	0.81342514	0.93855234	0.86668064	0.04513674	5
2021-03-25-07-30-CSLIBp	C	Dry	07	1.00379236	0.72817	0.185134	13.5846907	0.793443	0.96588287	0.82146917	0.0662069	5
2021-03-28-18-12-CSLIBp	C	Dry	18	1.01347443	1.613998	0.385278	16.0580323	0.67042985	0.9136448	0.73379704	0.00442331	0
2021-03-30-16-18-CSLIBp	C	Dry	16	1.01385446	0.572328	0.157937	12.9791592	0.71385952	0.96477138	0.7399261	0.11862069	2
2021-03-31-05-00-CSLIBp	C	Dry	05	1.00904991	1.241238	0.274709	10.7448121	0.66340407	0.9235666	0.71830669	0.01598098	4
2021-04-01-16-54-CSLIBp	C	Dry	16	1.02717876	1.62147	0.462668	12.9464725	0.77931664	0.97729417	0.79742279	0.38953627	0
2021-04-04-09-12-CSLIBp	C	Dry	09	0.99569562	0.804621	0.175932	11.2636867	0.81539045	0.98122172	0.83099511	0.0616409	4
2021-04-04-15-00-CSLIBp	C	Dry	15	1.01723111	1.321905	0.291297	11.2879801	0.73959144	0.92646285	0.79829584	0.00722949	5
2021-04-06-09-24-CSLIBp	C	Dry	09	0.99774833	1.247641	0.325533	10.2439234	0.84741053	0.9619755	0.88090656	0.03167658	3
2021-04-06-16-18-CSLIBp	C	Dry	16	1.02250312	0.926168	0.209557	12.1894653	0.7006928	0.94814738	0.73901253	0.07277051	1
2021-04-08-05-24-CSLIBp	C	Dry	05	0.99916398	0.55075	0.145883	13.8328055	0.76866913	0.97033429	0.79216939	0.0803805	4
2021-04-11-10-06-CSLIBp	C	Dry	10	1.0009465	1.530868	0.357021	14.1085135	0.80094326	0.94078026	0.85136061	0.01065398	6
2021-04-13-08-00-CSLIBp	C	Dry	08	0.98192155	1.779629	0.464481	10.3392295	0.84928424	0.97908454	0.86742688	0.40085612	3
2021-04-13-14-18-CSLIBp	C	Dry	14	0.99902284	0.703347	0.165441	15.7717724	0.73102003	0.96627441	0.75653461	0.12366231	4
2021-04-14-11-18-CSLIBp	C	Dry	11	0.99495751	2.036574	0.59721	13.6124279	0.86306037	0.96779492	0.89178022	0.12470868	2
2021-04-19-13-12-CSLIBp	C	Dry	13	1.00853888	1.751315	0.455607	6.34744518	0.86695011	0.98158042	0.88321863	0.08347206	2
2021-04-20-06-30-CSLIBp	C	Dry	06	0.9972725	1.898682	0.516052	14.7755242	0.82526124	0.96962667	0.85111235	0.29593341	2
2021-04-21-14-36-CSLIBp	C	Dry	14	1.00253061	1.577024	0.388744	6.61324141	0.85394433	0.9844325	0.86744833	0.22259215	3
2021-04-22-07-42-CSLIBp	C	Dry	07	0.99887039	1.779686	0.475464	8.03754228	0.88231064	0.95784675	0.92113967	0.02497027	5
2021-04-24-10-18-CSLIBp	C	Dry	10	0.99921284	1.677788	0.470455	6.05794144	0.88335175	0.97159278	0.909179	0.05659929	6
2021-04-24-16-12-CSLIBp	C	Dry	16	1.06286607	1.700465	0.419437	13.7385557	0.83345916	0.95829959	0.86972714	0.05935791	1
2021-04-25-13-36-CSLIBp	C	Dry	13	0.98629107	1.300901	0.286574	8.81701883	0.7861479	0.92630787	0.84868965	0.00799049	4
2021-04-26-06-30-CSLIBp	C	Dry	06	0.97271193	1.678234	0.436325	9.30526316	0.81743878	0.98304512	0.83153739	0.7850654	2
2021-04-26-10-48-CSLIBp	C	Dry	10	1.00673087	0.58704	0.157964	13.0740783	0.80861351	0.98231571	0.8231707	0.12451843	4
2021-05-01-16-06-CSLIBp	C	Wet	16	0.99142719	1.695691	0.451316	12.9420272	0.8566998	0.97941668	0.87470412	0.19976219	2
2021-05-02-11-12-CSLIBp	C	Wet	11	0.95881012	1.926205	0.539847	11.8703873	0.86849716	0.9698402	0.89550543	0.16480381	3
2021-05-09-10-24-CSLIBp	C	Wet	10	0.983756	1.775979	0.500971	20.7324806	0.79308112	0.98005253	0.80922307	0.63315101	1
2021-05-11-09-30-CSLIBp	C	Wet	09	0.99940411	1.899183	0.537531	17.1132189	0.84762007	0.98078115	0.86422956	0.62425684	3

2021-05-11-10-42-CSLIBp	C	Wet	10	0.97841157	2.025034	0.630509	14.5049513	0.81036907	0.98253316	0.82477529	0.77117717	4
2021-05-15-13-00-CSLIBp	C	Wet	13	0.98374138	1.639656	0.427428	12.9730668	0.78655063	0.96823555	0.81235463	0.32551724	3
2021-05-19-15-48-CSLIBp	C	Wet	15	0.98137646	1.527626	0.356274	9.57307826	0.77288067	0.96140763	0.80390528	0.17303211	2
2021-05-21-15-42-CSLIBp	C	Wet	15	1.02282961	1.418294	0.32827	12.0055743	0.84775991	0.97707298	0.8676526	0.13969084	2
2021-05-22-09-12-CSLIBp	C	Wet	09	0.99309071	2.015889	0.621626	19.5303432	0.80900514	0.97538477	0.82942154	0.54982164	3
2021-05-22-14-54-CSLIBp	C	Wet	14	0.99569258	2.243244	0.844891	12.3727553	0.82565569	0.94990271	0.86920027	0.09036861	3
2021-05-23-05-06-CSLIBp	C	Wet	05	0.99784937	2.094074	0.69225	18.9911196	0.80278346	0.98580862	0.81434006	0.93831153	3
2021-05-28-12-54-CSLIBp	C	Wet	12	0.99377736	1.705028	0.462444	14.8795858	0.81805103	0.98241896	0.8326906	0.75338882	2
2021-05-29-17-00-CSLIBp	C	Wet	17	0.98926448	1.340848	0.298841	12.7870243	0.76172563	0.98465881	0.77359347	0.69060642	1
2021-06-01-09-00-CSLIBp	C	Wet	09	0.99074167	2.047151	0.655521	19.2020955	0.79935705	0.97787466	0.81744327	0.59714625	3
2021-06-05-05-42-CSLIBp	C	Wet	05	0.99844072	1.926189	0.573546	13.9842223	0.8219833	0.98578521	0.83383611	0.8991201	5
2021-06-06-05-06-CSLIBp	C	Wet	05	1.0138766	1.876136	0.561533	18.2010396	0.81873316	0.9855507	0.83073672	0.91819263	3
2021-06-06-05-48-CSLIBp	C	Wet	05	1.02225902	1.99417	0.619942	12.2015494	0.81834606	0.98277381	0.83269015	0.800761	3
2021-06-06-12-00-CSLIBp	C	Wet	12	0.9885277	1.853847	0.549995	17.920085	0.80707164	0.98436385	0.81989159	0.79148633	4
2021-06-08-18-12-CSLIBp	C	Wet	18	1.01755254	1.123352	0.244654	11.6633656	0.74024545	0.97963626	0.75563296	0.57807372	0
2021-06-09-16-24-CSLIBp	C	Wet	16	1.00566843	1.464349	0.335267	13.1718114	0.84934181	0.98094205	0.865843	0.53436385	1
2021-06-16-06-24-CSLIBp	C	Wet	06	0.99340735	1.782286	0.493971	18.5693171	0.81522564	0.98448086	0.82807667	0.82868014	3
2021-06-18-08-30-CSLIBp	C	Wet	08	0.95374192	2.298718	0.949975	4.16946612	0.86207839	0.98294854	0.87703309	0.83234245	2
2021-06-23-15-00-CSLIBp	C	Wet	15	1.01277356	1.934047	0.547859	7.519977	0.88238289	0.96182844	0.91740153	0.04623068	3
2021-06-30-07-48-CSLIBp	C	Wet	07	0.98440172	1.731553	0.434432	7.36393211	0.8177323	0.95481251	0.85643232	0.08684899	2
2021-07-01-08-48-CSLIBp	C	Wet	08	0.98000455	1.682589	0.464963	10.5951478	0.82990717	0.96727598	0.85798386	0.18644471	3
2021-07-04-14-06-CSLIBp	C	Wet	14	1.01651066	1.145245	0.274251	7.31336028	0.79813683	0.96644169	0.82585099	0.24984542	1
2021-07-06-07-42-CSLIBp	C	Wet	07	0.97107316	2.090372	0.65532	9.76767887	0.82284161	0.97438562	0.84447224	0.45845422	3
2021-07-06-15-36-CSLIBp	C	Wet	15	1.01249051	1.725114	0.43536	9.66651354	0.84301923	0.9749613	0.86466944	0.28366231	2
2021-07-07-10-00-CSLIBp	C	Wet	10	0.99559346	1.568666	0.368168	8.38215785	0.8373476	0.97825853	0.85595738	0.33788347	4
2021-07-11-11-12-CSLIBp	C	Wet	11	0.96526177	1.710376	0.454479	13.2699588	0.79788865	0.96374201	0.82790689	0.23595719	4
2021-07-12-08-54-CSLIBp	C	Wet	08	1.0035885	0.90637	0.200634	9.63623078	0.7809747	0.98146818	0.79572085	0.29241379	2
2021-07-14-14-12-CSLIBp	C	Wet	14	1.00199855	0.765401	0.183379	12.3340009	0.78169033	0.97569861	0.80115963	0.15134364	4

2021-07-15-05-12-CSLIBp	C	Wet	05	0.97495786	2.037406	0.612243	11.3590132	0.8299538	0.97167079	0.85415123	0.37821641	5
2021-07-15-06-36-CSLIBp	C	Wet	06	0.98939148	2.097655	0.688514	13.3087419	0.81922099	0.98290339	0.8334705	0.83747919	2
2021-07-15-12-48-CSLIBp	C	Wet	12	0.97307706	1.933796	0.547874	11.6696791	0.83806299	0.97950318	0.85560007	0.52394768	2
2021-07-18-13-30-CSLIBp	C	Wet	13	0.99903925	1.626986	0.403327	13.5752648	0.85337583	0.96020129	0.8887468	0.04851367	4
2021-07-19-08-00-CSLIBp	C	Wet	08	1.0037775	1.80203	0.469718	8.57875169	0.83567512	0.98287993	0.85023114	0.2983591	5
2021-07-22-08-24-CSLIBp	C	Wet	08	0.99916295	2.277833	0.884406	7.15684445	0.85181495	0.97812602	0.87086421	0.55124851	3
2021-07-22-10-12-CSLIBp	C	Wet	10	1.01162782	1.383475	0.306958	9.54447661	0.85508195	0.97707282	0.87514659	0.19671819	4
2021-07-22-18-48-CSLIBp	C	Wet	18	1.00279567	2.271777	0.877659	7.32784242	0.87863328	0.98834558	0.88899398	0.78216409	0
2021-07-25-07-00-CSLIBp	C	Wet	07	0.99089131	2.292874	0.931323	6.40982885	0.88140954	0.98633438	0.89362143	0.77350773	1
2021-07-26-18-48-CSLIBp	C	Wet	18	1.0089625	2.002558	0.601392	6.87131902	0.87368321	0.98314097	0.88866524	0.06644471	0
2021-07-27-18-00-CSLIBp	C	Wet	18	1.00875232	0.432502	0.141196	5.20931665	0.78534068	0.94747029	0.82888158	0.00242568	0
2021-07-29-10-54-CSLIBp	C	Wet	10	0.99722722	2.036351	0.639403	18.5756365	0.78698929	0.98844178	0.79619185	0.99310345	1
2021-07-29-12-00-CSLIBp	C	Wet	12	1.0373924	1.204291	0.257202	12.7517194	0.80691009	0.97782282	0.82521094	0.27129608	1
2021-07-30-12-36-CSLIBp	C	Wet	12	0.98823179	2.103636	0.684512	16.0489008	0.80234462	0.98798008	0.81210607	0.87800238	0
2021-08-03-15-24-CSLIBp	C	Wet	15	1.0179814	2.199577	0.747101	6.97326159	0.86047593	0.97329769	0.88408298	0.32532699	0
2021-08-04-11-30-CSLIBp	C	Wet	11	0.99735605	2.196159	0.747924	7.72553712	0.85898331	0.97041007	0.8851756	0.34216409	1
2021-08-05-09-42-CSLIBp	C	Wet	09	0.99772711	1.794284	0.462208	6.12072554	0.85384408	0.97060993	0.87969848	0.28137931	4
2021-08-06-12-48-CSLIBp	C	Wet	12	0.99943426	1.92206	0.53984	8.19581195	0.85405697	0.95466948	0.89461012	0.08889417	1
2021-08-16-08-54-CSLIBp	C	Wet	08	1.00511058	0.248819	0.119687	6.89629823	0.77282585	0.9706931	0.7961588	0.00656362	3
2021-08-22-11-54-CSLIBp	C	Wet	11	0.99158619	1.186831	0.251311	17.8111889	0.77713879	0.98792447	0.78663786	0.85170036	2
2021-08-26-17-00-CSLIBp	C	Wet	17	1.00167397	1.355189	0.298287	11.5369823	0.81006198	0.98622912	0.821373	0.24941736	0
2021-08-27-10-00-CSLIBp	C	Wet	10	1.00697825	1.814376	0.485706	7.71895841	0.86210129	0.97408376	0.88503815	0.24732461	3
2021-08-27-13-30-CSLIBp	C	Wet	13	1.00308037	1.689754	0.422647	9.82241155	0.8129044	0.94487048	0.86033421	0.07001189	2
2021-08-30-11-42-CSLIBp	C	Wet	11	1.01981177	1.2629	0.275435	9.624731	0.59691731	0.91805875	0.65019512	0.01170036	1
2021-09-03-17-48-CSLIBp	C	Wet	17	1.00309972	1.784923	0.458424	9.24006942	0.83585006	0.95575498	0.87454428	0.11871582	0
2021-04-22-09-00-CSLIBp	C	Dry	09	0.97508435	1.918303	0.55405	16.9203789	0.82299305	0.96936613	0.84900125	0.34027778	5
2021-09-05-09-00-CSLIBp	C	Wet	09	1.00411282	0.491348	0.158632	6.21579319	0.77936917	0.96310478	0.80922573	0.00180737	2
2021-09-06-12-54-CSLIBp	C	Wet	12	1.01259193	0.806791	0.220696	9.45693539	0.72441598	0.96997476	0.74684003	0.24351962	3

2021-09-07-06-18-CSLIBp	C	Wet	06	0.99580094	2.053775	0.658172	7.76253127	0.84791147	0.9852818	0.86057762	0.78439952	0
2021-09-12-07-30-CSLIBp	C	Wet	07	1.00563299	2.223903	0.794237	12.7179689	0.81407119	0.92174127	0.88318839	0.0134126	3
2021-09-18-18-12-CSLIBp	C	Wet	18	1.03880268	0.947484	0.205691	12.0863117	0.76819048	0.96975889	0.79214585	0.17693222	0
2021-09-19-18-30-CSLIBp	C	Wet	18	1.00594981	0.954239	0.204741	11.6844385	0.83498597	0.97949565	0.85246522	0.06725327	0
2021-09-20-18-12-CSLIBp	C	Wet	18	0.99416454	1.003123	0.216766	11.0424468	0.71875128	0.95991756	0.74876356	0.11300832	0
2021-09-23-06-18-CSLIBp	C	Wet	06	1.0052724	0.792284	0.206418	10.0786259	0.77658105	0.96422575	0.8053934	0.10787158	7
2021-09-23-17-06-CSLIBp	C	Wet	17	1.02354166	0.968019	0.210656	8.82643126	0.73001308	0.96701468	0.75491417	0.14368609	1
2021-09-25-13-48-CSLIBp	C	Wet	13	0.98881197	2.162835	0.757966	12.0059708	0.81191862	0.98881644	0.82110145	0.97241379	1
2021-09-25-15-00-CSLIBp	C	Wet	15	1.00706884	1.30125	0.311335	16.7365679	0.76908136	0.9744669	0.78923292	0.3705113	0
2021-09-26-13-48-CSLIBp	C	Wet	13	1.03332757	1.177299	0.26819	9.57885553	0.74618943	0.9723538	0.76740527	0.23286564	0
2021-10-01-06-54-CSLIBp	C	Wet	06	1.00612996	1.349088	0.325789	13.3557465	0.83391634	0.98193247	0.84926038	0.23010702	3
2021-10-08-16-00-CSLIBp	C	Wet	16	1.01466471	0.9247	0.205745	10.7507298	0.77845037	0.97053917	0.80208032	0.13702735	1
2021-10-21-17-54-CSLIBp	C	Wet	17	1.00989271	1.243945	0.267595	10.9528813	0.79838694	0.97938241	0.81519428	0.1412604	0
2021-11-29-13-54-CSLIBp	C	Dry	13	1.00884518	0.019218	0.100607	6.42725198	0.69725067	0.96508491	0.72247599	0.08370987	1
2021-12-10-07-42-CSLIBp	C	Dry	07	1.00881978	1.347152	0.335753	10.7293433	0.82551657	0.97320426	0.84824595	0.35548157	3
2021-12-31-16-00-CSLIBp	C	Dry	16	1.03211253	0.51916	0.149007	10.191926	0.66213269	0.96573916	0.6856227	0.24813318	0
2022-01-05-09-00-CSLIBp	C	Dry	09	1.02034454	0.632043	0.155692	7.24220624	0.63833366	0.96605858	0.66076082	0.26568371	1
2022-01-06-07-48-CSLIBp	C	Dry	07	1.01336653	0.494244	0.150996	13.5751881	0.75104277	0.96701805	0.77665848	0.11300832	3
2022-01-13-17-18-CSLIBp	C	Dry	17	1.00770974	1.793708	0.468384	16.8126913	0.8552377	0.9854445	0.86786999	0.1465874	0
2022-01-14-16-00-CSLIBp	C	Dry	16	1.00879407	1.226207	0.26375	12.7404881	0.7588098	0.97490964	0.77833859	0.24870392	0
2022-01-15-11-24-CSLIBp	C	Dry	11	1.0129746	0.366496	0.126756	8.49118643	0.73646759	0.96009494	0.76707788	0.07724138	1
2022-01-17-18-12-CSLIBp	C	Dry	18	1.00995606	1.277503	0.292422	13.8393781	0.79251046	0.96652791	0.81995611	0.07667063	0
2022-01-18-11-54-CSLIBp	C	Dry	11	1.01450064	0.776892	0.177636	10.1078011	0.76226192	0.97380129	0.78276947	0.24799049	1
2022-01-21-08-42-CSLIBp	C	Dry	08	1.01706037	0.422717	0.136168	18.095932	0.66457232	0.96079066	0.69169315	0.15067777	2
2022-01-24-07-30-CSLIBp	C	Dry	07	1.01110488	0.320939	0.11888	6.48655477	0.68903759	0.95517453	0.72137349	0.03828775	3
2022-01-25-08-36-CSLIBp	C	Dry	08	1.01070554	1.116124	0.246085	10.057205	0.78720236	0.98385872	0.80011728	0.35919144	4
2022-01-25-14-00-CSLIBp	C	Dry	14	1.0087176	1.506622	0.355207	14.6982112	0.8280533	0.97328549	0.85078151	0.11414982	0
2022-01-28-16-00-CSLIBp	C	Dry	16	1.02673492	0.655035	0.154503	11.9359429	0.63687134	0.92413546	0.68915367	0.01640904	0

2022-01-29-10-48-CSLIBp	C	Dry	10	1.00456228	0.663684	0.155311	14.8885229	0.83009397	0.98139036	0.84583466	0.13674197	0
2022-01-30-17-54-CSLIBp	C	Dry	17	1.02444194	0.851502	0.187363	15.2807508	0.81811305	0.97290386	0.84089815	0.04513674	0
2022-01-31-18-00-CSLIBp	C	Dry	18	1.02611808	1.935663	0.577499	14.0067244	0.87748561	0.9812316	0.89426962	0.03447087	0

The filename of each *.wav* file based on the YYYY-MM-DD-HH-mm timestamp (WAV), Normalized Acoustic Complexity index (nACI), Acoustic Diversity index (ADI), Inverse Acoustic Evenness index (1-AEI), Bioacoustic index (BI), Acoustic Entropy index (H), Temporal Entropy Index (Ht), Spectral Entropy Index (Hf), Acoustic Richness Index (AR), and Number of species heard (SH).
