# Supplementary material for: Acoustic indices as proxies for bird species richness in an urban green space in Metro Manila
Source: PLoS One. 2023 Jul 28;18(7):e0289001. doi: 10.1371/journal.pone.0289001 (PMC10381043; doi:10.1371/journal.pone.0289001)
Supplement: Attachment — Submitted filename: Responses to Reviewers V2.docx [file pone.0289001.s007.docx]

**Manuscript Title:** Acoustic indices as proxies for bird species richness in an urban green space in Metro Manila

**Reviewer Comments & Author Rebuttals**

**Reviewer Reports on the Initial Version:**

Reviewers’ comments:

Reviewer #1 (Remarks to the Author):

The authors have satisfactorily responded to all points raised. As such, I can recommend the publication of this paper in its present form.

Reviewer #3 (Remarks to the Author):

The authors mostly addressed the reviewer’s suggestions. Still there are some other minor points to be clarified and stressed.

Fig 2: remove gradient from the caption and specify min and max values in dB.

The frequency response that you obtained reveals that there could be significant differences among the used sensors. This may imply that also the indices calculation could be affected by it. This limitation needs to appear in the Conclusions.

**Author Rebuttals to Initial Comments:**

**Reviewer #1**

*The authors have satisfactorily responded to all points raised. As such, I can recommend the publication of this paper in its present form.*

We thank the reviewer for their comments and suggestions, which greatly improved this manuscript.

**Reviewer #3**

*The authors mostly addressed the reviewer’s suggestions. Still there are some other minor points to be clarified and stressed.

Fig 2: remove gradient from the caption and specify min and max values in dB.*

Thank you for the suggestion. We have removed the gradient from the caption and specified the minimum and maximum values in dB. The amplitude ranges from -60 dB to -44 dB (raw) and -60 dB to -29 dB (pre-processed).

*The frequency response that you obtained reveals that there could be significant differences among the used sensors. This may imply that also the indices calculation could be affected by it. This limitation needs to appear in the Conclusions.*

We have included this limitation of low-cost recorders in the Conclusions and it reads as follows “As the frequency response of our recorders was not flat over the entire frequency range, which impacts the calculation of acoustic indices, low-cost recorders should be calibrated before use in ecoacoustic studies to offset this limitation. We also recognize the importance of pre-processing sound data, such as applying high-pass filters, noise reduction, and signal amplification, to account for differences among recorders, remove unwanted ambient sounds, and improve the detection of bird species.”
